# Investigating genetic-and-epigenetic networks, and the cellular mechanisms occurring in Epstein–Barr virus-infected human B lymphocytes via big data mining and genome-wide two-sided NGS data identification

**DOI:** 10.1371/journal.pone.0202537

**Published:** 2018-08-22

**Authors:** Cheng-Wei Li, Bo-Ren Jheng, Bor-Sen Chen

**Affiliations:** Laboratory of Control and Systems Biology, National Tsing Hua University, Hsinchu, Taiwan; University of Nebraska-Lincoln, UNITED STATES

## Abstract

Epstein–Barr virus (EBV), also known as human herpesvirus 4, is prevalent in all human populations. EBV mainly infects human B lymphocytes and epithelial cells, and is therefore associated with their various malignancies. To unravel the cellular mechanisms during the infection, we constructed interspecies networks to investigate the molecular cross-talk mechanisms between human B cells and EBV at the first (0–24 hours) and second (8–72 hours) stages of EBV infection. We first constructed a candidate genome-wide interspecies genetic-and-epigenetic network (the candidate GIGEN) by big database mining. We then pruned false positives in the candidate GIGEN to obtain the real GIGENs at the first and second infection stages in the lytic phase by their corresponding next-generation sequencing data through dynamic interaction models, the system identification approach, and the system order detection method. The real GIGENs are very complex and comprise protein–protein interaction networks, gene/microRNA (miRNA)/long non-coding RNA regulation networks, and host–virus cross-talk networks. To understand the molecular cross-talk mechanisms underlying EBV infection, we extracted the core GIGENs including host–virus core networks and host–virus core pathways from the real GIGENs using the principal network projection method. According to the results, we found that the activities of epigenetics-associated human proteins or genes were initially inhibited by viral proteins and miRNAs, and human immune responses were then dysregulated by epigenetic modification. We suggested that EBV exploits viral proteins and miRNAs, such as EBNA1, BPLF1, BALF3, BVRF1 and miR-BART14, to develop its defensive mechanism to defeat multiple immune attacks by the human immune system, promotes virion production, and facilitates the transportation of viral particles by activating the human genes *NRP1* and *CLIC5*. Ultimately, we propose a therapeutic intervention comprising thymoquinone, valpromide, and zebularine to act as inhibitors of EBV-associated malignancies.

## Introduction

The Epstein–Barr virus (EBV), also known as human herpesvirus 4 (HHV-4), was first identified in 1964 by Michael Epstein and Yvonne Barr [1]. Epstein and Barr investigated Burkitt’s lymphoma (BL), and demonstrated that the malignant cells contained viral particles with characteristic herpesvirus morphology; they subsequently reported the first evidence of a tumor-associated virus in humans. EBV is a ubiquitous virus that seriously infects more than 90% of the global population. It mainly infects human B lymphocytes and epithelial cells, and is therefore associated with a variety of their malignancies, including BL, Hodgkin’s lymphoma (HL), gastric cancer (GC), nasopharyngeal carcinoma (NPC), T/NK cell lymphoma, and AIDS- or transplantation-associated lymphoma [2]. Like other herpesviruses, EBV exists in both latent and lytic phases with respect to viral gene expression [3, 4]. Upon infection, EBV establishes a lifelong latency in the infected cells, predominantly in human B cells.

In the latent phase, the genomic DNA of EBV transforms into the episome of the memory B cells, but only a limited subset of the viral latent genes is expressed. Thus, the human immune system cannot target those genes easily, which allows EBV to evade the human immune response; this latent mode of infection is beneficial to EBV because it allows it to persist. In contrast, during the lytic phase of infection, nearly all the viral lytic genes of EBV are transcribed[2]. It is essential that the lytic cycle produces infectious viral particles, enabling the spread of the virus from cell to cell and form host to host. *In vitro* assays indicate that hypoxia, B cell receptor (BCR) stimulation, and transforming growth factor-beta (TGF-β) can also induce a lytic replication cycle under some circumstances[5]. Lytic reactivation causes a cascade of viral lytic genes expressed in a temporally regulated manner in three stages: immediate–early (IE), early, and late, which are accompanied by the replication of viral genomes and the production of viral particles. Following EBV genome encapsidation, DNA packaging, and virion release, new infectious virions can infect new cells in the same host and new hosts[6].

EBV, a double-stranded DNA virus, latent genomes can assemble into chromatin structures with different histone and epigenetic modification patterns that can regulate viral gene expression. These epigenetic regulators include ubiquitin proteins, histone acetyltransferases, deacetylases, and methyltransferases as well as DNA methyltransferases. They also influence EBV, an oncogenic herpesvirus, pathogenesis by evading human immune detection, resisting apoptosis, and driving human cell carcinogenesis.

Because a study by Tina O'Grady et al. has reported the two-sided next-generation sequencing (NGS)-based genome-wide time-course expression data of human B cells and EBV during EBV infection [7], dynamic system modelling, applied to the molecular characterization of transcription regulations, microRNA (miRNA) repressions, long non-coding RNA (lncRNA) regulations and protein–protein interactions (PPIs) (including their interactions with epigenetic enzymes), can be solved to identify the genome-wide interspecies genetic-and-epigenetic network (GIGEN) in this study. According to the gene expression profiles of the viral immediate–early (IE) lytic genes *BZLF1* and *BRLF1*, the early lytic genes *BMRF1*, *BBLF3*, *BBLF4*, *BGLF5*, *BNLF2A* and *BSLF1* and the late viral genes *BCRF1*, *BVRF2*, *BDLF1*, *BLLF1* and *BCLF1* during the infection[7], we identified the GIGEN at first stage, where the IE lytic genes and the early lytic genes are highly expressed, and the GIGEN at second stage, where the early lytic genes and the late viral genes are highly expressed. Furthermore, we determined more specific interactions, regulations, and gene/protein functions between humans and EBV by extracting the host–virus core networks (HVCNs) from the GIGENs to provide more information on drug targets for multi-molecule drug design. We then extracted the host–virus core pathways (HVCPs) from the HVCNs to investigate the relationship between the defensive and offensive human immune mechanisms and the antagonism strategies of EBV from the perspective of the core signaling pathways in the GIGENs. The HVCPs helped us understand in detail the significant events and their corresponding molecular mechanisms, such as genetic-and-epigenetic regulations and miRNA repressions, at the first and second stages of infection. Finally, we discussed the potential viral drug target proteins and miRNAs that are inferred from the HVCNs and HVCPs, supported by the EBV-related literature review.

According to the results, the proposed potential multi-molecule drugs can inhibit the switch from the latent phase to the lytic phase during viral reactivation, and can suppress the expression of some critical EBV lytic genes/proteins during EBV infection. This interrupts the production of virions, interferes with the transportation of viral particles, and destroys the viral defensive mechanisms.

## Results

### GIGENs of the first and second stages of the lytic phase of infection in EBV-infected B cells

**Fig 1:**
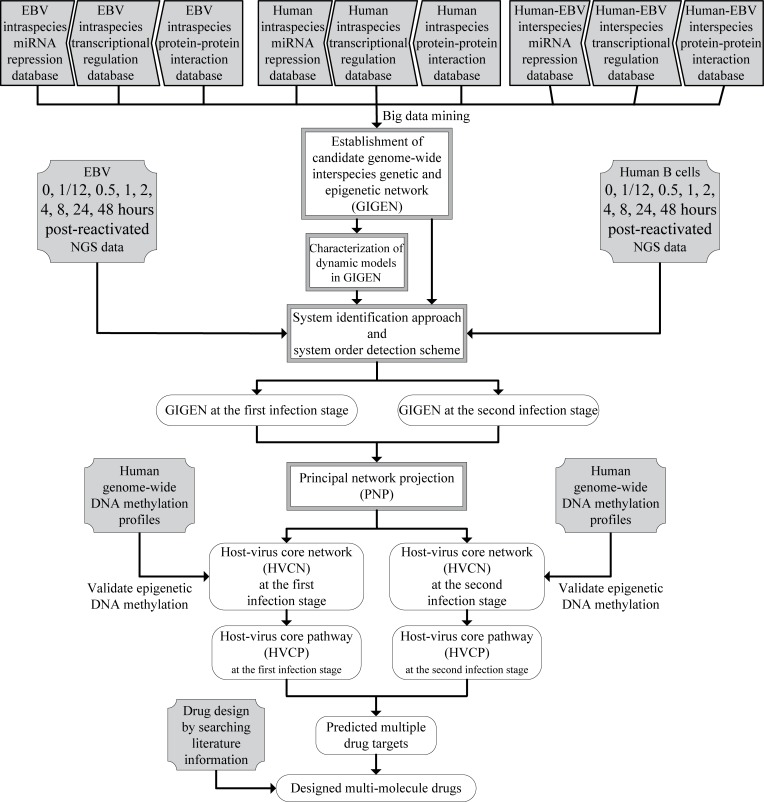
Flow chart describing the constructing of the interspecies GIGEN network and HVCP for the multiple drug targets and potential multi-molecule drugs via the systems biology approach. The blocks filled with gray indicate the input information exploited in this process, obtained by big data mining to establish the candidate GIGEN, NGS data to obtain the gene expression of human and EBV during the lytic phase, genome-wide DNA methylation profiles to verify the epigenetic regulation of DNA methylation of human genomes, and literature information on multi-molecule drugs for multi-molecule drug design based on the predicted drug targets. The blocks with gray frames represent the systems biology approach exploited to provide the identified information in our results; and the white blocks with solid line frames contain the results obtained from these processes.

**Fig 2:**
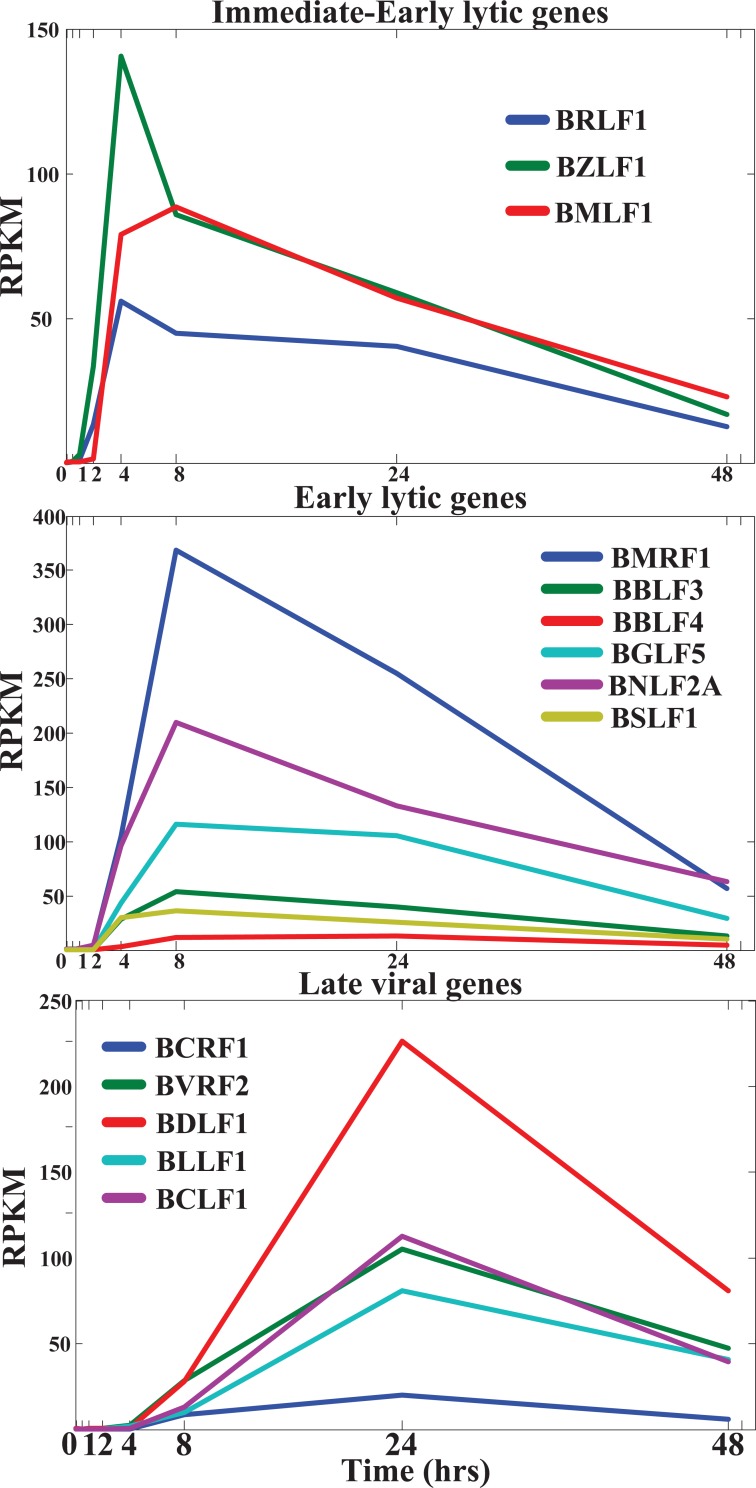
Changes of gene expression levels of typical lytic genes based on classification from the literature review. The NGS data of these typical lytic genes were sequenced by the Reads Per Kilobase per Million mapped reads (RPKM) procedure at every time-point, and are classified as immediate–early (IE), early, and late stages during the lytic phase on the basis of the classification from the literature review.

**Fig 3:**
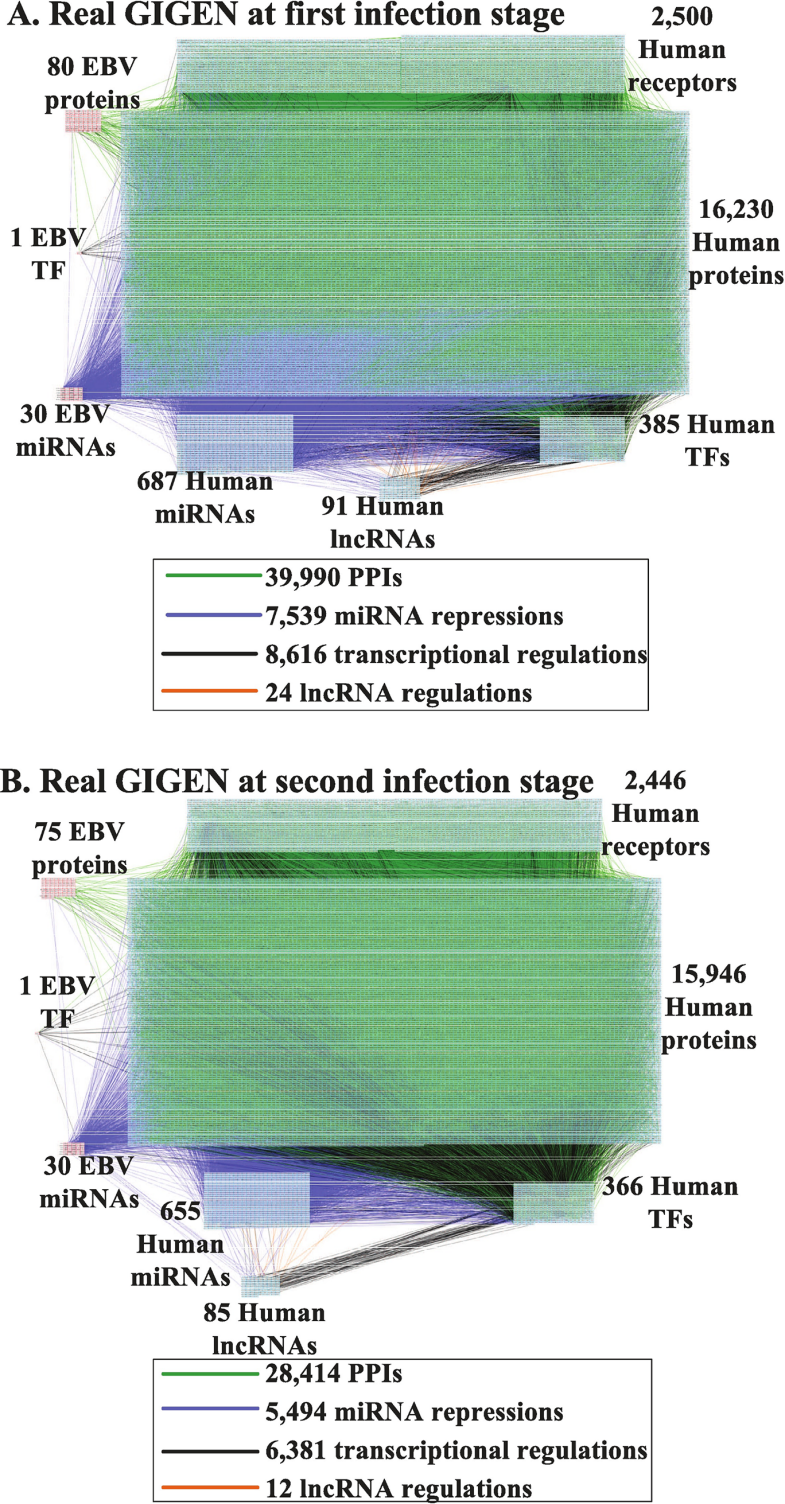
**Real interspecies GIGENs of the first (A) and second (B) infection stages in the lytic phase.** The nodes with red frames correspond to the EBV proteins/TF/miRNAs; the nodes with blue frames indicate the human receptors/proteins/TFs/miRNAs/lncRNAs; the edges in green denote the PPIs of humans, EBV, and human-EBV; the edges in purple represent the miRNA repressions of miRNAs on intraspecies and interspecies genes; the edges in black represent the transcriptional regulations of TFs on intraspecies and interspecies genes; the edges in orange signify the lncRNA regulations of lncRNAs on human genes.

A flow chart of the strategy for constructing the GIGENs, HVCNs and HVCPs in human B cells infected with EBV lytic infection from 0 to 72 hours post reactivation is shown in Fig 1. According to the gene expression profiles of the viral IE lytic genes, the early lytic genes and the late viral genes in Fig 2, we classified the lytic phase into the first infection stage from 0 to 24 hours, where the IE lytic genes and the early lytic genes are highly expressed, and the second infection stage from 8 to 72 hours, where the early lytic genes and the late viral genes are highly expressed. The GIGENs of the first and second infection stages are shown in Fig 3A and 3B, respectively. The numbers of nodes and edges are recorded in Table 1A and 1B, respectively. Among these edges, three human TF complexes were identified in the real GIGENs. The first was ARNT::AHR, which had 31 human TF-gene pairs at the first infection stage and 15 pairs at the second infection stage; the second was HIF1A::ARNT, which had 16 human TF-gene pairs at the first infection stage and 3 pairs at the second infection stage; and the third was NFE2L1::MAFG, which had 38 human TF-gene pairs at the first infection stage and 54 pairs at the second infection stage. There were no remarkable differences in the number of nodes between the first and second infection stages during the lytic phase. Nevertheless, the edges of the real GIGENs between both infection stages in Table 1B revealed significant differences in the human PPIs (first: 39,846/second: 28,325), interspecies PPIs (first: 86/second: 45), and EBV-miRNAs to human-genes (first: 914/second: 620). Each edge at the first and second infection stages in the real GIGENs is shown in S1A Table. The results indicate that there are more interactions in B cells, and between B cells and EBV, which contribute to the enhancement of the transcriptional replication of viral particles in B cells. EBV protects itself from silencing through EBV miRNA, and inhibits some human biological processes, such as the immune response, apoptosis, autophagy, and metabolism. We then carried out DAVID analyses of target genes in GIGENs to evaluate the specific functions between the first and second infection stages (Table 2A and 2B, respectively) [8].

**Table 1:** Numbers of nodes (A) and edges (B) in candidate GIGEN and the real GIGENs at the first and second infection stages.

A. The number of nodes			
Nodes	Candidate GIGEN	First infection stage	Second infection stage
V_T	1	1	1
V_M	43	30	30
V_P	85	80	75
H_T	2,688	385	366
H_M	1,326	687	655
H_L	186	91	85
H_P	18,227	16,230	15,946
H_R	2,880	2,500	2,446
total	43,689	20,004	19,604
B. The number of edges			
Edges	Candidate GIGEN	First infection stage	Second infection stage
V_P ↹ V_P	301	58	44
V_T → V_M	5	0	1
V_M ┥V_G	67	1	2
H_P ↹ H_P	23,570,918	39,846	28,325
H_T → H_G	897,805	8,424	6,222
H_T → H_M	7,471	86	71
H_T → H_L	1,335	79	65
H_M ┥H_G	815,889	6,582	4,837
H_M ┥H_M	215	6	3
H_M ┥H_L	1,796	26	16
H_L→H_G	1,948	24	12
V_P ↹ H_P	5,135	86	45
V_T → H_G	1,252	15	14
V_M ┥H_G	39,558	914	620
V_M ┥H_M	39	2	4
V_M ┥H_L	175	6	7
H_T → V_G	680	6	4
H_T → V_M	675	6	4
H_M ┥V_G	1,708	1	4
H_M ┥V_M	10	1	1
total	25,346,982	56,169	40,301

V_T: TFs of EBV, V_M: miRNAs of EBV, V_P: proteins of EBV, V_G: genes of EBV, H_T: TFs of human B cells, H_M: miRNAs of human B cells, H_L: lncRNAs of human B cells, H_P: proteins of human B cells, H_G: genes of human B cells, H_R: receptors of human B cells, ↹: PPIs, →: transcriptional regulations, ┥: miRNA repressions.

**Table 2:** Specific functional annotations of target genes in the real GIGENs at the first infection stage (A) and at the second infection stage (B) obtained by DAVID analysis.

A.Real GIGEN at the first infection stage	
Functional annotation	*p*-value
GO:0000082~G1/S transition of mitotic cell cycle	6.36E-12
GO:0019058~viral life cycle	3.3E-10
GO:0045815~positive regulation of gene expression, epigenetic	4.58E-10
GO:0006414~translational elongation	7.18E-10
GO:0006996~organelle organization	7.93E-10
GO:0043488~regulation of mRNA stability	4.5E-9
A.Real GIGEN at the second infection stage	
Functional annotation	*p*-value
GO:0006351~transcription, DNA-templated	7.22E-11
GO:0006614~SRP-dependent cotranslational protein targeting to membrane	1.23E-6
GO:0006334~nucleosome assembly	1.48E-5
GO:0042787~protein ubiquitination involved in ubiquitin-dependent protein catabolic process	1.87E-5
GO:0097193~intrinsic apoptotic signaling pathway	3.85E-5
GO:0018279~protein N-linked glycosylation via asparagine	5.17E-5

As indicated in Table 2A, during the first stage of infection, EBV begins lytic replication at the *ori-Lyt* site, the initial site for viral lytic replication, and initiates the viral life cycle, which involves decoding of genomic information, translation of viral mRNA by human ribosomes, genome replication, and the assembly and release of viral particles. The protein Zta encoded by *BZLF1*, an immediate–early gene of EBV in the lytic phase, easily binds to the response elements of hypermethylation; furthermore, there is an early viral protein (SM/M) that enhances the posttranscriptional modification of EBV genes [9]. Moreover, the overexpression of an EBV protein kinase (BGLF4) causes phosphorylation of the translational elongation factor, which strengthens the output and stability of the nuclear mRNA [10–12]. At this stage, human B cells enter the G1/S transition of the cell cycle, in which DNA replication is initiated, and prepare to undergo mitotic processing and, simultaneously, the formation of some organelles, such as autophagosomes, ribosomes, and the cytoskeleton.

During the second stage of infection, new virions are released from the B cells to infect other uninfected B cells or epithelial cells. Table 2B indicates that after replication, nucleosomes are formed to protect the integrity of the viral genome; they are then assembled, packaged, and exported. Subsequently, the virions are dependent on signal recognition particle (SRP) protein to help them target the membrane. There are advanced glycation end product (AGE) receptors on the cell surface; the complexes formed by products and receptors can activate intracellular signaling pathways, such as the intrinsic apoptotic signaling pathway or the ubiquitin-dependent protein catabolic signaling pathway, to initiate reactions within the cells and degrade target proteins.

### HVCNs of the first and second stages of the lytic phase of infection in EBV-infected B cells

#### Significant cellular processes of the HVCNs in the lytic replication cycle

**Fig 4:**
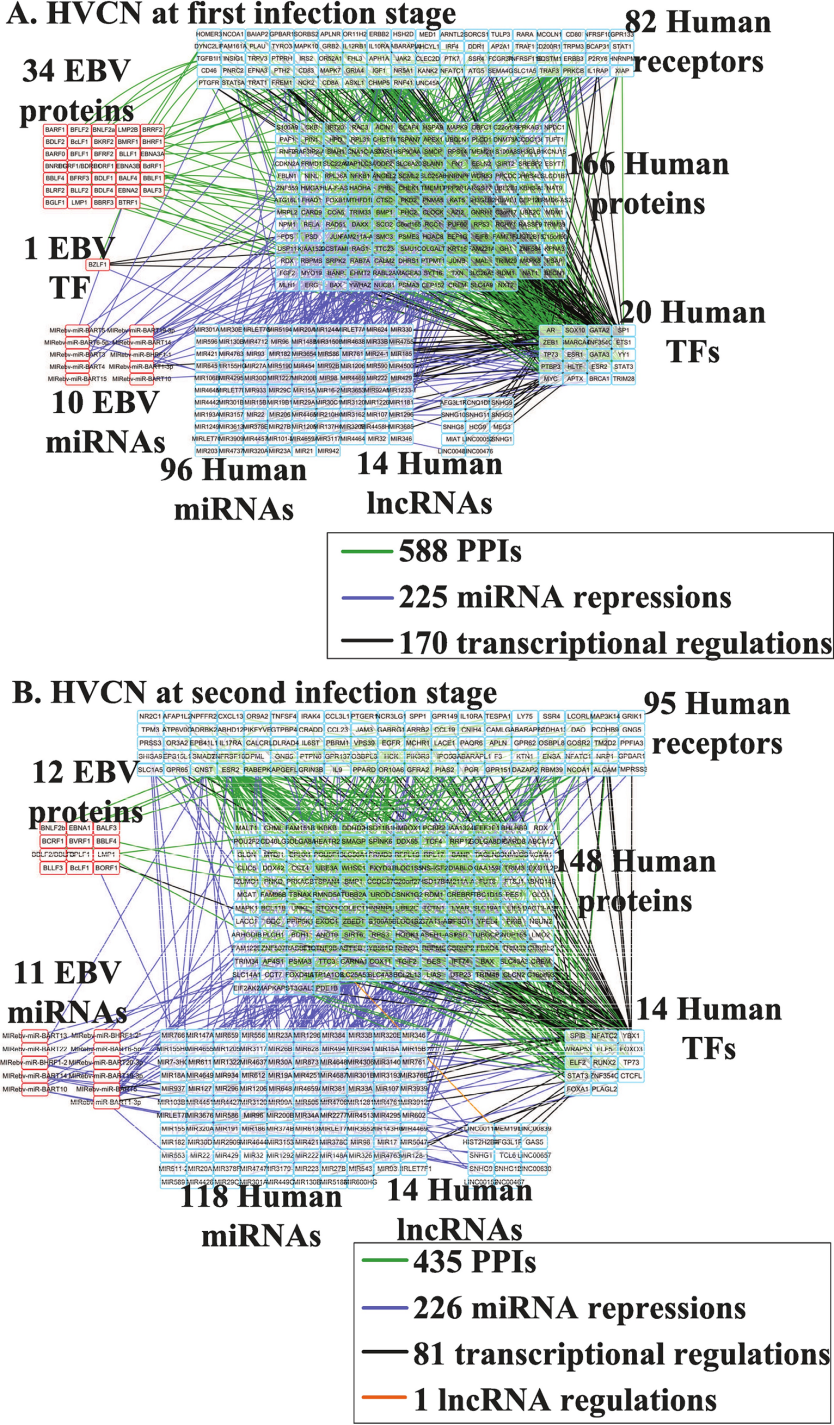
**HVCNs at the first (A) and second (B) infection stages in the lytic phase.** The nodes with red frames correspond to the EBV proteins/TF/miRNAs; the nodes with blue frames indicate the human receptors/proteins/TFs/miRNAs/lncRNAs; the edges in green denote the PPIs of humans, EBV, and human-EBV; the edges in purple represent the miRNA repressions of miRNAs on intraspecies and interspecies genes; the edges in black represent the transcriptional regulations of TFs on intraspecies and interspecies genes.

Furthermore, in order to obtain the core cellular functions in humans and EBV during lytic infection, we extracted HVCNs from the real GIGENs at both stages of infection using the PNP method from the perspective of the major network structure, as shown in Fig 4A and 4B, respectively; the numbers of nodes and edges are recorded in Table 3A and 3B, respectively. Each edge of the HVCNs at the first and second infection stages is shown in S1B Table. Based on the identified interactive parameters of the core PPINs in the HVCNs in Eq (12) at the first and second infection stages, the calculated statistical significance (*p* value) of the edges in the core PPINs, is shown in S1C Table (See Materials and Methods). The results reveal 98.33% and 99.68% statistically significant edges (*p* value≤ 0.05) at the first and second infection stages, respectively. Moreover, in contrast to the real GIGENs, we found no edges for human TF complexes regulating the target genes in the HVCNs (Fig 4). There was a marked difference in the number of nodes among the EBV proteins (the first infection stage: 34/the second infection stage: 12) during the lytic phase in Table 3A. This may account for the importance of the early-expressed EBV genes, which are required for the cellular functions of replication to help produce new viral particles, and the simultaneous prevention of premature death in the human cells. However, owing to the viral life cycle of EBV, late-expressed EBV genes may be expressed less than early-expressed genes in preparation for entering the latent phase, in which nearly all viral genes are silenced to evade the human immune system. Furthermore, as shown in Table 3B, there were significant differences between the edges in both infection stages in intraspecies PPIs (human, first: 510/second: 419; EBV, first: 42/second: 8), interspecies PPIs (first: 36/second: 8), human-TFs to human-genes (first: 126/second: 67), human-TFs to human-miRNAs (first: 24/second: 7), and human-TFs to human-lncRNAs (first: 16/second: 4). These data indicate that EBV mainly affects human PPIs through protein–protein interaction with humans, and human-TFs further transcriptionally regulate genes, miRNAs, and lncRNAs. To evaluate the specific functions for the human genes during EBV infection, we also analyzed target genes in HVCNs at both infection stages using DAVID, and the results are presented in Table 4A and 4B.

**Table 3:** Numbers of nodes (A) and edges (B) in HVCN at the first and second stages of infection.

A. The number of nodes		
Nodes	First infection stage	Second infection stage
V_T	1	0
V_M	10	11
V_P	34	12
H_T	20	14
H_M	96	118
H_L	14	14
H_P	166	148
H_R	82	95
total	423	412
B. The number of edges		
Edges	First infection stage	Second infection stage
V_P ↹ V_P	42	8
V_T → V_M	0	0
V_M ┥V_G	1	0
H_P ↹ H_P	510	419
H_T → H_G	126	67
H_T → H_M	24	7
H_T → H_L	16	4
H_M ┥H_G	190	185
H_M ┥H_M	4	1
H_M ┥H_L	4	5
H_L→H_G	0	1
V_P ↹ H_P	36	8
V_T → H_G	3	0
V_M ┥H_G	22	23
V_M ┥H_M	1	4
V_M ┥H_L	1	6
H_T → V_G	0	1
H_T → V_M	1	2
H_M ┥V_G	1	1
H_M ┥V_M	1	1
total	983	743

V_T: TFs of EBV, V_M: miRNAs of EBV, V_P: proteins of EBV, V_G: genes of EBV, H_T: TFs of human, H_M: miRNAs of human, H_L: lncRNAs of human, H_P: proteins of human, H_G: genes of human, H_R: receptors of human, ↹: PPIs, →: transcriptional regulations, ┥: miRNA repressions.

**Table 4:** Specific functional annotations of target genes in HVCNs at the first infection stage (A) and at the second infection stage (B) obtained by applying the DAVID analysis.

A.HVCN at the first infection stage	
Functional annotation	*p*-value
GO:0042493~response to drug	1.79E-08
GO:0043388~positive regulation of DNA binding	1.37E-05
GO:1902895~positive regulation of pri-miRNA transcription from RNA polymerase II promoter	6.55E-05
GO:2000378~negative regulation of reactive oxygen species metabolic process	1.17E-04
GO:0051090~regulation of sequence-specific DNA binding transcription factor activity	1.64E-04
GO:0016236~macroautophagy	2.14E-04
B.HVCN at the second infection stage	
Functional annotation	*p*-value
GO:0006919~activation of cysteine-type endopeptidase activity involved in apoptotic process	2.37E-04
GO:0032212~positive regulation of telomere maintenance via telomerase	3.20E-04
GO:0051897~positive regulation of protein kinase B signaling	0.001802
GO:0006954~inflammatory response	0.006715
GO:0070374~positive regulation of ERK1 and ERK2 cascade	0.011796
GO:0015031~protein transport	0.02314

Table 4A shows two crucial cellular functions at the first infection stage in response to drugs and macroautophagy. Drugs change the activity of lytic genes and stimulate or induce reactivation of EBV from the latent phase to the lytic phase. Faggioni et al. suggested that autophagy is blocked in the late stage of degrading microbiological infections during EBV replication [13]. This block enables EBV to hijack the autophagic vesicles for intracellular transportation, thereby enhancing viral production[14]. Table 4B shows that protein transport and inflammatory response are two specific cellular functions at the second stage of EBV infection. New virions may be conveyed in the autophagic vesicles mentioned above and transported to the plasma membrane. Upon membrane lysis, these virions are released from the cell to infect other cells. During this process, the human immediate defense system detects xenobiotics and elicits the inflammatory response, which triggers the immune system against infection by the new virions. For the purpose of adapting to EBV infection, it would be useful to identify post-translation epigenetic modifications in HVCNs that regulate certain intracellular signaling pathways.

## Discussion

### HVCPs at the first and second stages of infection during the lytic replication cycle

#### New virion production through host–virus cross-talk interactions at the first stage of infection

**Fig 5:**
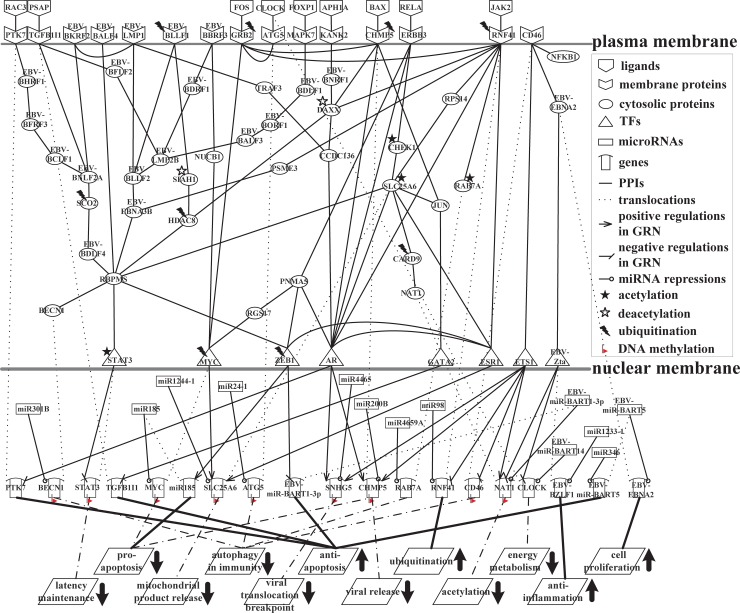
HVCP in B cells infected with EBV at the first infection stage in the lytic phase. The solid lines indicate the protein–protein interactions; the dotted lines denote the translocations, including protein translations and miRNA transcriptions; the solid lines that end in arrows, bars, or circles stand for positively transcriptional regulations, negatively transcriptional regulations, and miRNA repressions, respectively; the dash-dot lines represent the gene functions that are inhibited; the bold lines mean the gene functions that are promoted; the short arrows beside the gene functions signify susceptibility to repression or enhancement.

**Fig 6:**
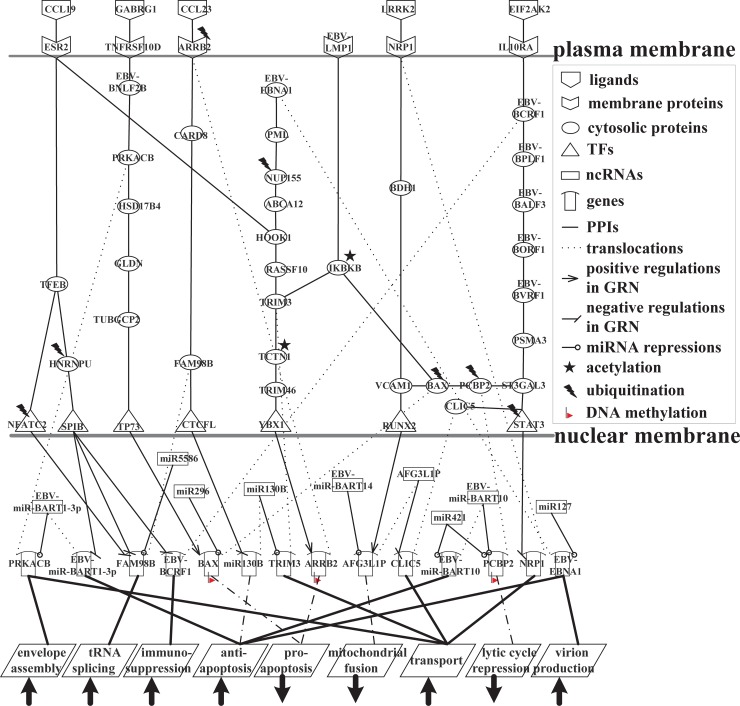
HVCP in B cells infected with EBV at the second infection stage of the lytic phase. The solid lines indicate protein–protein interactions; the dotted lines denote the translocations, including protein translations and miRNA transcriptions; the solid lines that end in arrows, bars, or circles stand for positively transcriptional regulations, negatively transcriptional regulations, and miRNA repressions, respectively; the dash-dot lines represent the gene functions that are inhibited; the bold lines mean the gene functions that are promoted; the short arrows beside the gene functions signify susceptibility to repression or enhancement.

Although extensive research has been carried out on the cross-talk between virus and host, little is known about how cellular functions are performed through host–virus PPIs and real genetic-and-epigenetic network from the perspective of intracellular signaling transduction pathways. Thus, by further using the significant changes in gene expression between the first and second infection stages determined by *p*-values and the PNP method, we extracted HVCPs during the lytic phase, which is divided into the first and second infection stages as shown in Figs 5 and 6, respectively. The EBV intraspecies connections, including {LMP1, BKRF2}, {LMP1, BLLF2, EBNA3B}, {BCLF1, BFRF3}, and {EBNA2, Zta}, and the interspecies connections, including {MAPK7, BDLF1} and {PSMA3, BVRF1} in Figs 5 and 6 can be supported by the literature [15]. Human cells can affect the behavior of proteins to accommodate rapidly varying circumstances, whereas EBV genes, including EBV miRNA (also called miR-BARTs) have ways to manipulate the operation of viral proteins and even human proteins for the purposes of survival and the propagation of the progeny in this microenvironment. EBV microRNAs in particular can evade the human immune response through the anti-apoptosis strategies in the HVCPs [16]. These are commonly referred to as epigenetics or post-translational modifications, and they include DNA methylation, ubiquitination, acetylation, and deacetylation. Following lytic reactivation of EBV from latent infection to lytic infection, initially the viral IE lytic genes *BZLF1* and *BRLF1* are expressed. They then collaboratively activate the promotors of the early lytic genes, which encode the viral replication proteins. Next, viral genome replication occurs in which the late viral genes are transcribed. The late EBV genes encode certain structural proteins required for viral genome encapsidation into infectious viral particles [5].

**Fig 7:**
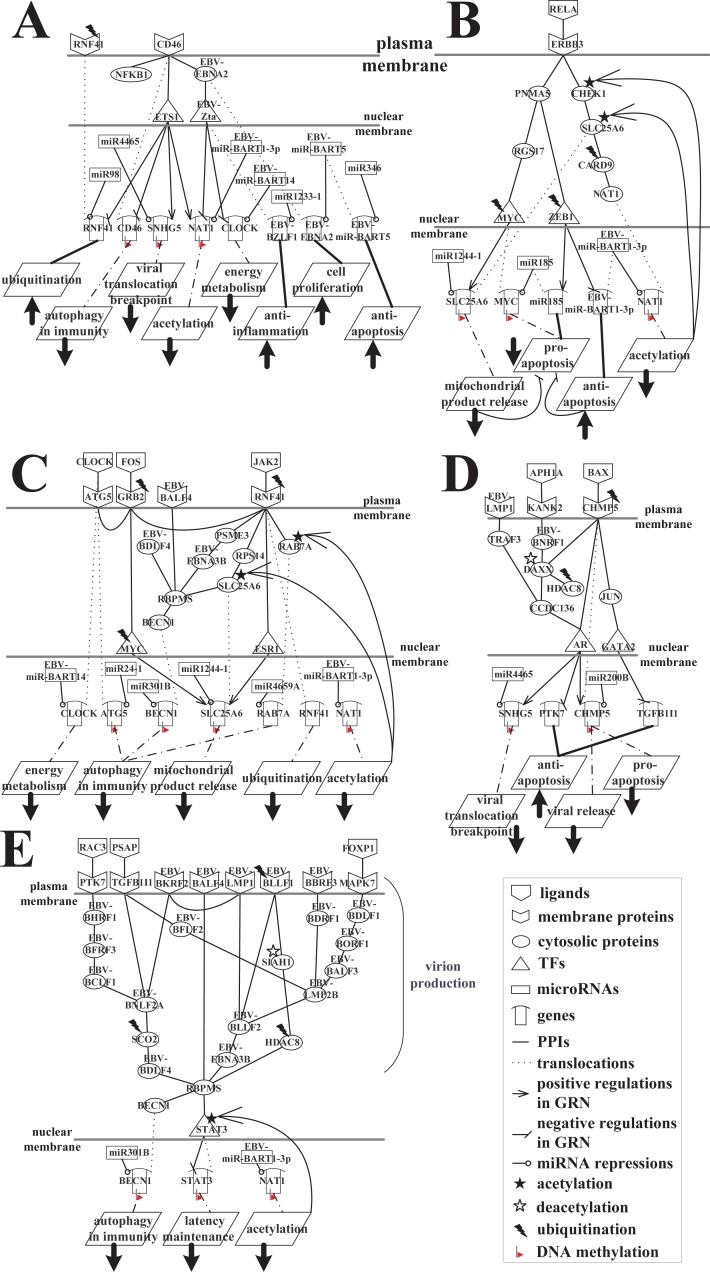
Signaling pathways of the interspecies molecular mechanisms based on the HVCP in Fig 5 at the first infection stage during EBV infection. (A) The core pathways promoting cell proliferation and the impairment of immune information by the EBNA2-mediated pathway with receptor CD46; (B) the pro-apoptotic human pathway blocked by EBV through ubiquitination and acetylation; (C) the autophagy mechanism blocked by EBV through the involvement of viral BALF4, BDLF4, EBNA3B, miR-BART14, and miR-BART1-3p; (D) the complete progression of lytic production through the impairment of pro-apoptosis and the promotion of viral translocation and anti-apoptosis; (E) the promotion of the integrated production of infectious virions by silencing autophagy and inhibiting the expression of STAT3.

We further investigated the cellular mechanisms of the first infection stage by dividing the HVCP in Fig 5 into five parts, as shown in Fig 7A–7E. In Fig 7A, human cells send a cell proliferation signal via NFKB1 to induce the production of more immune cells, and an immune-related signal to antagonize EBV lytic reactivation. In Fig 7A, receptor CD46 receives the immunity and cell growth signals via NFKB1 binding. It can be supported that the interaction between NFKB1 and CD46 in human B cells promotes immune responses [17]. CD46 is a co-stimulatory factor for the development of T-helper cells, and works through IL-10 release, suppressing immune responses to prevent autoimmunity. It has been proposed that the immune-evasive strategy of EBV appears to rely strongly on IL-10, and EBV itself also codes an IL-10 homologue, which is expressed during the lytic phase [18]. In Fig 7A, the viral protein EBNA2 interacts with CD46 to exploit its immune regulation property and directly induce an immunosuppressive phenotype. This results in CD46 being significantly downregulated (*p*-value = 5.73 × 10^−16^) by NFKB1-mediated apoptosis, so the downstream expression of the human transcription factor ETS1 is downregulated (*p*-value = 3.76 × 10^−4^). In Fig 7A, the human genes *NAT1* and *SNHG5* are positively regulated by ETS1, whereas the human genes *CD46* and *RNF41* are negatively regulated by ETS1. The significantly low expression of NAT1 (*p*-value = 2.93 × 10^−37^) could be due to the low activity of ETS1, the inhibition of viral IE protein, Zta, the repression of viral miRNA, miR-BART1-3p, and DNA methylation (*p*-value = 4.23 × 10^−5^). The main function of NAT1, an acetyltransferase protein that functions as a xenobiotic metabolizing enzyme, is to impair xenobiotic substances by acetylation. The significantly low expression of SNHG5 (*p*-value = 1.32 × 10^−13^) could also be due to the low activity of ETS1, the repression of human miRNA, miR4465, and DNA methylation. *SNHG5* is a long non-coding RNA that suppresses the proliferation, migration, and invasion of infected cells. It also prevents the translocation of cell growth factors from the cytoplasm to the nucleus and interrupts viral translocation from the nucleus to the cytoplasm. It can be supported that ETS1 was thought to be involved in regulating chromosomal translocations in Human B cell non-Hodgkin lymphoma [19]. Therefore, it functions as a translocation breakpoint in B cell lymphoma [20, 21]. As mentioned above, the gene of human receptor CD46 is downregulated by the impairment of NFKB1, and DNA methylation (*p*-value = 3.89 × 10^−5^), so its effect on autophagy in immunity is reduced. Thus, the human body naturally attempts to exploit the functions of genes (*NAT1*, *SNHG5*, *CD46*) to defeat EBV during lytic reactivation, but EBV successfully evades attack by the human immune system. Furthermore, the markedly high expression of RNF41 (*p*-value = 8.21 × 10^−8^) is due to the low transcriptional inhibition of ETS1 and the low repression of miR98 (*p*-value = 4.84 × 10^−19^). In Fig 7A, RNF41 is degraded by ubiquitination when it functions as a receptor in the autophagy mechanism pathway blocked by EBV. It can be supported that RNF41 is a RING finger-containing protein, and has been investigated for its involvement in TLR-mediated responses, growth regulation, and inflammatory responses by promoting the ubiquitination of target proteins [22, 23]. Thus, RNF41 could cause the degradation of several proteins by ubiquitination during EBV infection.

Furthermore, owing to interaction with CD46 in Fig 7A, EBV evades immune attack by NFKB1. The cell growth signal then induces an immediate–early viral protein, Zta, for the transcriptional activation of early lytic genes via the viral protein EBNA2, which appears to be more efficient at upregulating genes that are involved in the proliferation and survival of infected cells [24]. The elevated activity of EBNA2 (*p*-value = 2.01 × 10^−24^) enables the proliferation of infected cells. *EBNA2* is repressed by the viral microRNA miR-BART5, which collaborates with EBNA2 to control the transcriptional regulation of lytic replicate genes during infected cell proliferation. However, the immunity signal induces the EBV immune-evasive strategy. EBV develops resistance to apoptosis by counteracting the pro-apoptotic function of p53 with miR-BART5 [25]. EBV-*miR-BART5* has a role in anti-apoptosis, and highly expressed EBV-*BZLF1* (*p*-value = 0.023) reduces inflammation, resulting in an innate immune response. In Fig 7A, EBV-*miR-BART5* and EBV-*BZLF1* are silenced by MIR346 and MIR1233-1, respectively, but the low expression of MIR346 and MIR1233-1 demonstrates that B cells still require other proteins to inhibit the progression of the lytic replication cycle. It can be supported that the upregulated MIR346 promoted apoptosis [26]. In Fig 7A, the human genes *CLOCK* and *NAT1* are transcriptionally inhibited by Zta, and repressed by miR-BART14 and miR-BART1-3p, respectively. CLOCK can cause energy metabolism and induce the progression of apoptosis and autophagy, but low CLOCK activity does not destroy viral proteins. The following is a brief overview of Fig 7A. In the first stage of EBV infection, the human proteins CLOCK, NAT1, SNHG5, CD46, and RNF41 are inhibited by epigenetic modifications, EBV proteins, miRNAs, or the low activity of TFs, and the human body is unable to defeat EBV. At the same time, EBV activates its defense mechanism to antagonize immune attacks through anti-inflammation and anti-apoptosis responses, thereby enhancing the proliferation of infected cells via EBV EBNA2.

Fig 7B shows RELA, which is a subunit of NF-κB and is involved in many biological processes such as inflammation, immunity, differentiation, cell growth, tumorigenesis, and apoptosis. In Fig 7B, the human receptor ERBB3 can transmit signals from RELA via PNMA5 to the human TFs ZEB1 and MYC, which induce pro-apoptosis and trigger apoptosis by releasing mitochondrial products and inhibiting viral anti-apoptosis. The result also shows that the human TFs MYC and ZEB1 are subjected to proteolysis via ubiquitination by the ubiquitin-proteasome pathway related proteins MUL1 and UBE2E1, respectively, which decreases the transcriptional regulation of the target genes. Thus, at the first stage of infection, the low activity of genes *miR185* and *MYC* results in a lack of pro-apoptotic activity. Human gene *MYC* is also controlled by DNA methylation (*p*-value = 7.95 × 10^−5^). Furthermore, the low activity of MYC at the first infection stage affects the low expression of SLC25A6, which suffers from the repression of human miR1244-1, the transcriptional silence of DNA methylation (*p*-value = 1.14 × 10^−2^), and the acetylation by acetyltransferase protein KAT5. Therefore, SLC25A6 cannot trigger apoptosis through the release of mitochondrial products, which indirectly suppresses the progression of pro-apoptosis. Additionally, the low expression of ZEB1 (*p*-value = 1.72 × 10^−13^) is unable to transcriptionally inhibit EBV-miR-BART1-3p. This gives rise to elevated EBV-miR-BART1-3p expression, promoting anti-apoptosis against innate immune responses and pro-apoptosis, and successfully repressing the expression of the target gene *NAT1*. This protects EBV from acetylation by NAT1 during lytic replication. ERBB3 has been reported to be involved in mediating the regulations of acetylation and cell apoptosis through its signaling pathway [27, 28].

It has been reported that CHEK1 mediates cell cycle arrest in response to DNA damage and suppresses the proliferation of infected cells [29]. SLC25A6 is ubiquitously expressed in all tissues, and is involved in the regulation of cell viability and apoptosis triggering [30]. CARD9 plays an important regulatory role in cell apoptosis and the innate immune response to a number of intracellular virus [31]. Thus, we suggested that the human proteins CHEK1, SLC25A6, CARD9, and NAT1 form a signaling transduction pathway that mediates cell apoptosis to promote the destruction of EBV. However, NAT1 is repressed by EBV miR-BART1-3p, and the inactivation of NAT1 by acetylation results in the activation of CHEK1 and SLC25A6, which are subjected to acetylation by acetyltransferase proteins MGAT4B and KAT5, respectively; MIB2 ubiquitinates CARD9. It is thought these epigenetic modifications have the following three characteristics: CHEK1 cannot suppress the expression of viral proteins in infected cells; SLC25A6 cannot translocate ADP into mitochondria and ATP into the cytoplasm, decreasing the release of mitochondrial products and triggering apoptosis; and CARD9 is unable to induce an immune response to defeat EBV. The cellular conditions mentioned above indicate that EBV miR-BART1-3p mediates the apoptotic dysfunction of human B cells for the purpose of survival. The result can be supported that miR-BART1 directly targets cellular tumour suppressor to dysregulate cell apoptosis. There are growing evidence supporting the pro-viral role of caspase/apoptotic pathway in viral replication [32–34].

In Fig 7C, human receptors RNF41, GRB2, and ATG5 interact with each other and signal immune or apoptotic information using ligands (JAK2, FOS, and CLOCK, respectively) to induce SLC25A6 via TFs, ESR1, and MYC. SLC25A6 can then trigger apoptosis by releasing mitochondrial products to counteract the invasion of EBV.

Human proteins ATG5 [35], RAB7A [36], RPS14 [37], and BECN1 [38] have been reported to be involved in autophagic response. Therefore, Fig 7C showed that the human body exploits ligands (JAK2, FOS, and CLOCK) to transmit autophagy mechanism signals through receptors to induce autophagy-related proteins (ATG5, RAB7A, and BECN1). However, EBV-miR-BART1-3p suppresses xenobiotic acetylation by repressing the human gene *NAT1* in Fig 7C. The inactive acetylation caused by NAT1 results in the activation of SLC25A6 and autophagy-related RAB7A, which are subjected to acetylation by acetyltransferase proteins KAT5 and CSGALNACT1, respectively (Fig 7C). Furthermore, human proteins (RNF41, GRB2, and MYC) are degraded by ubiquitination through the binding of ubiquitin-proteasome pathway related proteins UBE2K, USP46, and MUL1, respectively. Therefore, we suggested that the low expression of these human proteins leads to a reduction in the transmission of apoptotic and autophagic signals.

Fig 7C shows that EBV miR-BART14 represses the gene that encodes the CLOCK ligand, which reduces its expression (*p*-value = 6.16 × 10^−6^); CLOCK decreases energy metabolism and prevents the signal to autophagic-related ATG5. However, the low activity of ATG5 (*p*-value = 6.75 × 10^−32^) is not only due to the low expression of its ligand, CLOCK, but is also due to transcriptional inhibition by DNA methylation and repression by high human miR24-1 activity (*p*-value = 9.67 × 10^−7^). This affects the formation and elongation of autophagosomes via ATG5, which leads to a reduction in autophagy. MiR24-1 has been associated with autophagic response [39]. At the first stage of infection, SLC25A6, which has low acetylation activity and DNA methylation activity (*p*-value = 1.14 × 10^−2^), is repressed by human miR1244-1 and reduces transcriptional regulation via TFs, ESR1 and MYC. This leads to an inability to induce apoptosis by releasing mitochondrial products, and affects the autophagic pathway (RNF41, RPS14, SLC25A6, RBPMS, and BECN1); therefore, it cannot fulfil the functions of BECN1 and mediate the nucleation and maturation of autophagosomes.

In Fig 7C, BECN1 is repressed by the elevated expression of miR301B (*p*-value = 0.012), and is subjected to DNA methylation, which reduces its effects on autophagy and immunity. The low expression of autophagy-related RAB7A (*p*-value = 4.46 × 10^−8^) is due to acetylation and the repression of human miR4659A (*p*-value = 0.036). Downregulation of RAB7A further weakens lysosomal degradation by reducing the number of lysosomes and the fusion between autophagosomes and lysosomes. It has been supported that miR4659A directly regulated genes involved in autophagic response [40]. Therefore, the reduction of RAB7A indicates that the autophagy mechanism is blocked, as observed during lytic EBV replication.

In Fig 7C, upon EBV blocking of the progression of autophagy, viral protein EBNA3B negatively interacts with human protein PSME3, which promotes ubiquitination and proteasomal degradation, thereby inhibiting apoptosis. We speculated that viral EBNA3B hijacks the ubiquitination of RNF41 by negatively interacting with PSME3, so that EBV induces other ubiquitin proteins to act at the first and second infection stages in the lytic phase. EBNA3B positively interacts with human protein RBPMS, which plays a role as a coactivator of transcriptional activity; therefore, RBPMS helps EBV to employ autophagy-related BECN1. Furthermore, viral receptor BALF4 is an envelope glycoprotein that forms spikes at the surface of the virion envelope, so BALF4 is essential for attachment to the autophagosome surface. Therefore, we suggest that BALF4 is involved in the fusion of EBV virions and autophagosome membranes leading to EBV transportation to B cell membranes for lysis. Another viral protein, BDLF4, is important for the EBV lytic replication cycle, but is currently uncharacterized. We speculate that BDLF4 collaborates with BALF4 and EBNA3B to block autophagy and hijack the autophagic vesicles, thereby enhancing viral production and the intracellular transportation of virions. Viral proteins BALF4, BDLF4, and EBNA3B, and viral miRNAs miR-BART14 and miR-BART1-3p block autophagy in B cells and interfere with viral antigen presentation to prevent their degradation (see Fig 7C). It has been reported that BALF4 [41] and EBNA3B [42] are involved in autophagic response. Hence, blocking mediated by EBV can occur at different stages of the autophagy mechanism pathway, from the formation of autophagosomes to the degradation of lysosomes. Therefore, an understanding of the relationship between EBV and autophagy may aid the discovery of new approaches to manipulate EBV infection and lytic replication through autophagy control.

Fig 7D shows that BAX is a ligand that binds to human receptor CHMP5, which is involved in the degradation of surface receptor proteins and the formation of endocytic multivesicular bodies (MVBs). BAX assigns endosomal cargo proteins for incorporation into MVBs, and sometimes functions in membrane fission, such as the lysis of enveloped viruses. Thus, when triggered by BAX, CHMP5 exports pro-apoptotic signal molecules from B cells into the cytoplasm via endocytic MVBs and endosomal trafficking. CHMP5 then signals directly to the transcription factor AR, and indirectly to the TF GATA2 via JUN, which is involved in the TLR pathway. CHMP5 therefore induces pro-apoptosis and the interruption of translocation, and inhibits anti-apoptosis (as shown in Fig 7D). However, CHMP5 has low activity at the first infection stage because it is degraded following ubiquitination by the ubiquitin ligase protein HUWE1, which reduces the expression of CHMP5 so that the low activity of TFs (GATA2 and AR) causes a decrease in the transcriptional silencing of human genes *TGFB1I1* and *PTK7*, which are thereby highly expressed (*p*-value = 5.37 × 10^−3^ and *p*-value = 2.96 × 10^−13^, respectively). It can be supported that ubiquitination of CHMP5 could be associated with induction of anti-apoptosis [43]. This promotes the anti-apoptosis of B cells infected with EBV, and protects EBV from cell death.

In Fig 7D, another pathway mediated by EBV induces anti-apoptosis and reduces the interruption of viral translocation and pro-apoptosis. APH1A, an endoprotease that catalyzes the intramembrane cleavage of integral proteins and membrane protein ectodomain proteolysis [44], binds to the receptor KANK2, which controls cytoskeletal formation by regulating actin polymerization and promoting cell proliferation. However, the elevated expression of viral protein BNRF1 (*p*-value = 5.15 × 10^−4^), a tegument protein that plays a role in the suppression of human intrinsic defenses to enhance the activation and transcription of early viral genes, negatively interacts with receptor KANK2 to evade caspase-independent apoptosis. BNRF1 then interacts with human DAXX, and may regulate apoptosis in the cytoplasm, thereby disrupting the complex formed between DAXX and ATRX. Suppressing the DAXX–ATRX-dependent deposition of histone H3.3 on the viral chromatin allows viral transcription. The low expression of DAXX (*p*-value = 2.67 × 10^−3^) is due to the disruption of viral BNRF1, the deacetylation by a histone deacetylase protein (HDAC8), and the low activity of CHMP5.

In contrast, DAXX interacts negatively with CCDC136. The elevated expression of CCDC136 (*p*-value = 3.64 × 10^−60^) causes the inactivation of AR, as mentioned above and depicted in Fig 7D. Moreover, LMP1 can activate the NF-κB signaling pathway and induce anti-apoptosis [45] through a pathway involving TRAF3 and CCDC136 to reduce the activity of AR. This induces the downstream target gene *PTK7* to initiate anti-apoptosis. The human body naturally enables the pro-apoptotic influence of EBV proteins by CHMP5, which receives the BAX pro-apoptotic signal via endocytosis, but the degradation of CHMP5 by ubiquitination and the viral protein BNRF1-mediated anti-apoptotic pathway may cause the inactivation of human TFs (GATA2 and AR). Consequently, pro-apoptosis and interruption of viral translocation are impaired, promoting anti-apoptosis and the complete progression of lytic replication at the first stage of EBV infection.

As shown in Fig 7E, RAC3, a GTPase that belongs to the RAS superfamily of small GTP-binding proteins, regulates a wide variety of processes, including the control of cell growth, cytoskeletal reorganization, the activation of protein kinases, differentiation, movement, and lipid vesicle transport. PSAP is a mitochondrial pro-apoptotic protein that forms a complex with BAX when apoptosis is induced [46]. RAC3 conveys signals, including those inducing cell growth factor and the activation of protein kinases to PTK7; it plays a role in anti-apoptosis, and is involved with the receptor for viral protein, BHRF1. PSAP, a pro-apoptotic protein ligand, binds to a receptor, TGFB1I1, and plays a role in anti-apoptosis against the pro-apoptotic signal from PSAP. Therefore, the diminished expression of PSAP (*p*-value = 2.73 × 10^−7^) has a negative impact on highly expressed TGFB1I1 (*p*-value = 5.37 × 10^−3^). Thus, the pro-apoptotic signal induces the anti-apoptotic function of TGFB1I1, which is also the receptor for viral proteins BFLF2 and BNLF2A (as shown in Fig 7E). The cell growth signal from receptor PTK7 is first received by a viral protein, BHRF1, which prevents the premature death of human cells during virus production. The viral protein BFRF3 then participates in the assembly of infectious particles by localizing on the outer surface of the capsid shell, thereby forming a layer between the capsid and the tegument. BFRF3 interacts with BCLF1 [15], which self-assembles to form an icosahedral capsid. The elevated expression of BCLF1 (*p*-value = 5.69 × 10^−4^) promotes the protection of the viral genome by the capsid. The anti-apoptotic signal induces the viral protein BNLF2A to evade HLA class I-restricted T cell immunity, and prevent TAP-mediated peptide transportation and subsequent loading. The function of viral BNLF2A is to activate SCO2, a copper chaperone that transports copper to the Cu site on cytochrome C oxidase subunit II (COX2), which assists the inner mitochondrial membrane in aerobic ATP production. Although SCO2 may trigger pro-apoptosis, it is degraded via ubiquitination by the ubiquitin ligase protein, G2E3. Thus, the pro-apoptotic function of SCO2 is inhibited so that EBV evades the immune response and apoptosis. It can be supported that BNLF2A contributes to cell survival through an immune evasion mechanism [47].

In Fig 7E, viral receptor BKRF2 is required for the fusion between viral and plasma membranes leading to EBV entry into the human B cell. Membrane fusion is mediated by the fusion machinery comprising *gB* (also called BALF4), and the heterodimer *gH* (also called BXLF2) /*gL* (also called BKRF2) may also be involved in the fusion between the virion envelope and the outer nuclear membrane during virion morphogenesis. Viral BKRF2 also interacts with viral BNLF2A, which can encode an inhibitor of transporter associated with antigen processing (TAP) to assist immune evasion. Additionally, viral BKRF2 interacts with a viral membrane protein, LMP1, [15] which acts as a CD40 functional homolog to prevent the apoptosis of the infected B-lymphocytes and drive their proliferation. LMP1 signaling leads to the upregulation of anti-apoptotic proteins and provides cell growth signals in the infected cells. LMP1 helps viral EBNA3B in immune evasion and hijacks the autophagy mechanism via viral BLLF2, an uncharacterized viral protein. It has been reported that LMP1 can regulate autophagy in EBV-infected B cells [48].

Furthermore, the host receptor TGFB1I1 interacts with viral BFLF2 and plays a fundamental role in virion nuclear egress. NEC1 (also called BFLF2) interacts with the newly formed capsid within the human nucleus via the vertexes, and NEC2 (also called BFRF1) directs it to the inner nuclear membrane. NEC1 then induces the budding of the capsid at the inner nuclear membrane and its envelopment into the perinuclear space. The NEC1/NEC2 complex has been reported to promote fusion between the enveloped capsid and the outer nuclear membrane, and subsequently release the viral capsid into the cytoplasm, where it binds to the secondary budding sites in the human Golgi network [49]. Therefore, the anti-apoptotic signal from human TGFB1I1 promotes the egress of the nuclear EBV virion.

In Fig 7E, BDRF1 receives the egress signal from its viral receptor, BBRF3. BDRF1 is a component of the molecular motor that can translocate viral genomic DNA into the empty capsid during DNA packaging. BDRF1 forms a tripartite terminase complex together with TRM1 (also called BALF3) and TRM2 in the human cytoplasm. Once the complex reaches the human nucleus, it interacts with the vertex of the capsid portal. This portal forms a ring in which genomic DNA is translocated into the capsid. BDRF1 has RNase activity, which plays an important role in the cleavage of concatemeric viral DNA into unit-length genomes. It can be supported that BBRF3 is involved in capsid budding via the inner nuclear membrane during egress, and participates in penetration through the plasma membrane during lytic infection [50].

As shown in Fig 7E, the human ligand FOXP transmits the anti-apoptotic signals to MAPK7, and MAPK7, which is an extracellular-signal-regulated kinase, promotes signaling transmission in the downstream signaling processes. Viral BDLF1 is a structural component of the icosahedral capsid. The capsid is composed of pentamers and hexamers of major capsid protein (MCP), which are linked together by heterotrimers called triplexes. These triplexes consist of a single molecule of triplex protein 1 (TRX1, also called BORF1) and two copies of triplex protein 2 (TRX2, also called BDLF1). Furthermore, BORF1 is required for the efficient transportation of BDLF1 to the nucleus, which is the site of capsid assembly. Thus, the signal promotes highly expressed BDLF1 (*p*-value = 9.65 × 10^−4^) to activate the structural molecule, and BDLF1 positively interacts with BORF1 to induce viral capsid assembly by transporting BDLF1 to the nucleus via BORF1. BORF1 then interacts with highly expressed viral BALF3 (*p*-value = 0.0486), a component of the molecular motor. BALF3 (also called TRM1) functions with BDRF1 (also called TRM3) and TRM2, and they collaboratively translocate EBV genomic DNA into the empty capsid during DNA packaging. It has been reported that BALF3 has endonuclease activity, and plays an essential role in the cleavage of concatemeric viral DNA into unit-length genomes [51].

As shown in Fig 7E, viral proteins BALF3, BDRF1, and BFLF2 interact with another viral membrane protein, LMP2B. LMP2B downregulates the functionality of LMP2A, which is able to block B cell activation. It is possible that LMP2B works in cooperation with LMP1 via viral BLLF2. The elevated expression of LMP2B (*p*-value = 0.0142) downregulates the LMP2A-mediated interruption of B cell signaling, whereas LMP1 activates B cells through the NF-κB, AP-1, and JAK/STAT pathways. Therefore, EBV can exploit LMP2B and LMP1, which collaboratively interact with BLLF2 to work in concert with EBNA3B [15], so that it contributes to the immune-evasive transport of complete virions via autophagic vesicles hijacked by EBV.

Furthermore, highly active BLLF2 (*p*-value = 1.89 × 10^−3^) interacts with a viral receptor, BLLF1, which initiates virion attachment to human B cells. This attachment triggers the fusion of the virion and the human membrane for the invasion of the human cell. However, in Fig 7E, BLLF1 is degraded via ubiquitination by the ubiquitin ligase protein SIAH1, and BLLF1 prevents the progression of viral replication from the aggression and interruption of uninfected human B cell. Moreover, viral BLLF1 negatively interacts with human ubiquitin ligase protein SIAH1, which decreases the degradation of ubiquitination via SIAH1 and reduces the activity of SIAH1 (*p*-value = 8.42 × 10^−4^). SIAH1 negatively interacts with human histone deacetylase HDAC8, so highly expressed HDAC8 (*p*-value = 6.39 × 10^−14^) can deactivate viral protein degradation. The mechanism has been exploited for antiviral strategies [52].

In Fig 7E, highly expressed human protein RBPMS (*p*-value = 2.35 × 10^−11^) interacts with viral proteins EBNA3B, BALF4, and BDLF4, and acts as a latent-lytic switch in EBV by negatively interacting with the human TF, STAT3. STAT3 can transcriptionally activate cellular PCBP2, which represses the expression of EBV lytic genes [53]. Consequently, EBV not only utilizes viral proteins to control RBPMS and further manipulate the activity of STAT3, but also uses viral miR-BART1-3p to repress the expression of human NAT1, which carries out acetylation, so that the reduction of acetylation via NAT1 induces the inaction of STAT3. Owing to the negative regulation of human RBPMS, acetylation by acetyltransferase LFNG, and DNA methylation (*p*-value = 8.62 × 10^−3^), low activity STAT3 (*p*-value = 1.35 × 10^−23^) is less able to maintain latency, which contributes to the complete progression of the EBV lytic replication cycle. STAT3 acetylation has also been reported in EBV-infected B cells [54]. The main perspective of Fig 7E is that EBV reduces autophagy in immunity and silences human STAT3 through viral proteins and viral miRNA. This maintains the integrated replication of infectious virions and also promotes anti-apoptosis against immune responses, the cleavage of viral DNA into unit-length genomes, viral DNA packaging, capsid and tegument assembly, and the transportation of virions via autophagic vesicles.

#### Transportation of viral particles through host–virus cross-talk interactions at the second stage of infection

**Fig 8:**
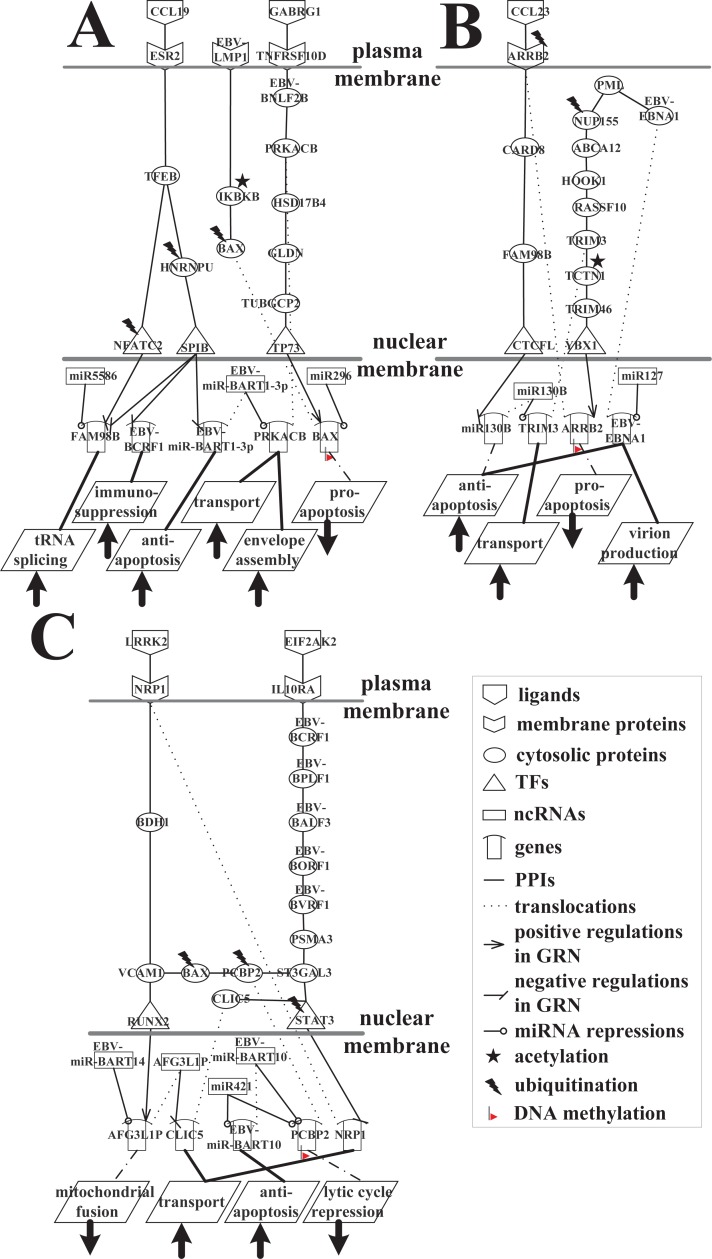
Signaling pathways of the interspecies molecular mechanisms based on the HVCP in Fig 6 at the second infection stage of EBV infection. (A) The core pathways of the enhancement of anti-apoptosis, immunosuppression, and genetic diversity pathways by EBV for the packaging, assembly, and transport of viral particles; (B) the promotion of virion production, vesicle trafficking, release, and anti-apoptosis pathways as a result of EBNA1-mediated PML disruption; (C) the maintenance of virion transportation by decreasing the repression of the lytic cycle and increasing anti-apoptosis activities.

The purpose of this study was to elucidate the pathogenesis mechanism of EBV-infected human B cells at the second stage of the lytic phase of infection (Fig 6). This is split into three parts in Fig 8A–8C to facilitate the investigation of the virion transportation process and the lysis of viral particles for further detailed analysis of cellular function.

Fig 8A showed that CCL19 transmits the immunoregulatory signal to TFEB via a human receptor, ESR2. TFEB activates the expression of CD40L in T cells, thereby participating in T cell-dependent antibody responses in the activated CD4(+) T cells. High activity TFEB (*p*-value = 1.34 × 10^−9^) can activate the expression of many lysosomal genes, and enables the positive regulation of autophagy and pro-apoptosis against EBV lytic infection. TFEB is able to directly induce FAM98B via the human TF NFATC2 to suppress tRNA splicing, but human NFATC2 is degraded via ubiquitination by a ubiquitin ligase protein, SIAH1, which leads to the reduced expression of NFATC2 (*p*-value = 0.002). This reduces the transcriptional inhibition of FAM98B. Thus, FAM98B enhances the ability of tRNA splicing to assist some viral late lytic genes in translation, and increases genetic diversity in packaging, assembly, and transportation. However, TFEB indirectly interacts with the human TF SPIB via HNRNPU, which is associated with pre-mRNA processing in the nucleus. HNRNPU affects pre-mRNA metabolism and triggers the apoptotic process, but the low activity of HNRNPU (*p*-value = 7.73 × 10^−63^) is due to ubiquitination by the ubiquitin ligase protein WWP1, which reduces the apoptotic function and transcriptional regulation of SPIB. The low activity of SPIB (*p*-value = 3.91 × 10^−24^) reduces the transcriptional inhibition of FAM98B. This causes FAM98B to counteract the repression of human miR5586 and to enhance the ability of tRNA splicing to increase viral and human genetic diversity in packaging, assembly, and transport, which can assist virion production in EBV infection. When SPIB expression is low, the transcriptional inhibition of EBV protein BCRF1 and viral miR-BART1-3p is reduced. Highly expressed viral BCRF1 (*p*-value = 1.97 × 10^−5^) encodes the viral homologue of IL-10 (vIL-10), which suppresses the immune response in the EBV lytic phase [55], whereas viral miR-BART1-3p has an anti-apoptotic function against the innate immune response and pro-apoptotic signals. Viral miR-BART1-3p reduces the repression of human PRKACB, regulating various cellular processes such as cell proliferation, the regulation of microtubule dynamics, envelope disassembly and reassembly, and the regulation of intracellular transport mechanisms and ion flux. Viral miR-BART1-3p increases the expression of PRKACB (*p*-value = 4.08 × 10^−4^), which normally carries out envelope assembly and intracellular transport, and promotes viral progeny production and subsequent lysis. The repressed SPIB has also been reported to inhibit apoptosis in late lytic cycle of the EBV-infect B cells [56].

In Fig 8A, GABRG1, which belongs to the ligand-gated ionic channel family [57], regulates the activity of ionic channels to transmit the anti-apoptotic signal to its receptor, TNFRSF10D, which is also a receptor for viral BNLF2B. TNFRSF10D, a member of the TNF-receptor superfamily, contains a truncated cytoplasmic death domain, which is incapable of inducing apoptosis but acts as an inhibitor to protect EBV proteins from TRAIL-mediated apoptosis [58]. TNFRSF10D interacts with the viral protein BNLF2B, and is very similar to the EBV protein BCRF1, which may evade the immune system and protect EBV. Viral BNLF2B positively interacts with human PRKACB, and viral miR-BART1-3p reduces the repression of human PRKACB, so EBV exploits PRKACB to promote envelope assembly and the intracellular transport of virions. Thus, viral BNLF2B transmits an anti-apoptotic signal that promotes viral particle production and affects the transcriptional activity of the human TF TP73 through human proteins HSD17B4, GLDN, and TUBGCP2. BAX is upregulated by the transcriptional regulation of TP73, but it is also subjected to the repression of human miR296, degradation via ubiquitination by the ubiquitin-conjugating enzyme UBE2C, and DNA methylation (*p*-value = 1.93 × 10^−3^); these factors reduce the pro-apoptotic function of human BAX. TNFRSF10D has been discovered to contribute disease progression in EBV-infected cells [59].

As shown in Fig 8A, LMP1, functions as a CD40 homolog [60], negatively interacts with human IKBKB, phosphorylating the inhibitor in the inhibitor-NF-κB complex, which causes the dissociation of the inhibitor and the activation of NF-κB. However, the pro-apoptotic function of human IKBKB is suppressed as a result of the negative interaction with LMP1 and inactivation via acetylation by the acetyltransferase GALNT7, so the expression of IKBKB is therefore low (*p*-value = 3.54 × 10^−20^). This directly affects the behavior of the human pro-apoptotic protein BAX, which belongs to the BCL2 protein family and acts as a pro-apoptotic regulator. During stress, BAX experiences a conformational change that results in translocation to the mitochondrion membrane, subsequently leading to the release of cytochrome C, which triggers pro-apoptosis. However, owing to the positive interaction with IKBKB (expressed at a low level), the transcriptional regulation of TP73, repression by human miR296, ubiquitination by the ubiquitin-conjugating enzyme UBE2C, and DNA methylation (*p*-value = 1.93 × 10^−3^), pro-apoptotic BAX is inhibited by EBV proteins BNLF2B and LMP1. It can be supported that inhibition of BAX leads to the repressed apoptosis in EBV-infected B cells [61]. This means that the human body loses one of the defensive mechanism that can antagonize EBV lytic infection. Fig 8A indicates that EBV not only evades immune suppression by viral BCRF1 and inhibits pro-apoptosis by viral BNLF2B and LMP1, but also exploits the anti-apoptotic function to promote the propagation of EBV progeny via viral miR-BART1-3p.

In Fig 8B, highly expressed EBNA1 (*p*-value = 8.81 × 10^−3^) is not repressed by human miR127 in this system, and impairs DNA repair, decreasing the activation of p53 and anti-apoptosis in response to DNA damage. This leads to increased cell survival by the silencing of PML proteins, and EBNA1 thereby protects virion production from apoptosis and promotes lytic infection. The interaction between viral EBNA1 and human CK2 kinase is important for EBNA1 to disrupt PML nuclear bodies and degrade PML. EBNA1 increases the association of CK2 with PML, thereby increasing the ability of CK2 to phosphorylate PML. Phosphorylation is a modification that is well known to trigger the polyubiquitylation and degradation of PML. It can be supported that PML disruption and silencing by EBNA1 are defensive mechanisms by which EBV may contribute to the advance of EBV-associated cancer [62].

In Fig 8B, disrupted PML can positively interact with NUP155, a nucleoporin protein that plays an important role in the assembly and function of the nuclear pore complex (NPC), which regulates the movement of molecules across the nuclear envelope (NE) [63]. NUP155 participates in the formation of the double membrane NE. However, it positively interacts with human-disrupted PML and is degraded by the ubiquitin protein USP3 via ubiquitination, leading to the low expression of NUP155 (*p*-value = 9.79 × 10^−12^). This affects transporter activity, the structural constitution of nuclear pores, and the binding and translocating ability of proteins during nucleocytoplasmic transport. Therefore, the damaged DNA signal is not successfully transported from the nucleus into the cytoplasm to induce DNA repair and trigger pro-apoptosis to block EBV. NUP155 negatively interacts with ABCA12, so the low expression of NUP155 results in the elevated expression of ABCA12 (*p*-value = 5.57 × 10^−5^). ABCA12 is a member of the superfamily of ATP-binding cassette (ABC) transporters, which transport various molecules across extra- and intracellular membranes. ABCA12 interacts with HOOK1, a member of the hook family of coiled-coil proteins, which bind to microtubules and organelles. HOOK1 links endocytic membrane trafficking to the microtubule cytoskeleton, and its complex can promote vesicle trafficking. HOOK1 interacts with TRIM3, a member of the cytoskeleton-associated recycling or transport (CART) complex, through RASSF10 to collaboratively mediate vesicular trafficking via TRIM3’s association with the CART complex and cooperatively promote the transport of virions. TCTN1 is a member of the family of secreted and transmembrane proteins that act as a barrier preventing the diffusion of transmembrane proteins. However, TCTN1 is inactivated via acetylation by the acetyltransferase NAA16, rendering it incapable of controlling the diffusion of the transmembrane proteins that allow EBV to hijack this function to assist virion transport. TCTN1 expressed at a low level (*p*-value = 1.19 × 10^−14^) causes the reduction of signal transmission to the human TF YBX1 via TRIM46, and therefore the low expression of YBX1 (*p*-value = 1.8 × 10^−19^) leads to a reduction of the transcriptional regulation of the human target gene, *ARRB2*. The pro-apoptotic function of ARRB2 is inhibited by the reduction of the transcriptional regulation of YBX1, ubiquitination by the ubiquitin protein HERPUD1, and DNA methylation (*p*-value = 3.05 × 10^−2^). It can be supported that the decreased YBX1 contributes to promote anti-apoptosis in EBV-infected cells [64].

As shown in Fig 8B, when ARRB2 is translocated to the plasma membrane as a human receptor, it receives the signal from a ligand, CCL23, which participates in immunoregulatory and inflammatory processes. CCL23 transmits the signal in response to inflammation to induce ARRB2 to carry out its pro-apoptotic function, but the activity of ARRB2 is suppressed. It subsequently induces the operation of human CARD8, which belongs to the caspase recruitment domain (CARD)-containing family of proteins. CARD8 enables the activation and expression of caspases or the NF-κB pathway, and may be a component of the inflammasome, a protein complex that participates in the activation of pro-inflammatory caspases. However, ARRB2 positively interacts with CARD8, reducing the expression of CARD8 and inactivating it (*p*-value = 7.67 × 10^−19^), and influences the inactivation of pro-inflammatory responses so that it promotes viral immune evasion. Inactivated CARD8 causes the activation of human FAM98B. FAM98B can increase the ability of tRNA splicing to assist the EBV lytic phase in the production of viral particles. FAM98B drives the highly expressed human TF CTCFL (*p*-value = 1.49 × 10^−54^) to transcriptionally inhibit human miR130B. Human miR130B promotes cell growth and self-renewal, and its normal expression increases cell viability, reducing cell death and decreasing the expression of apoptosis-related proteins [65]. Nevertheless, in spite of the fact that the inhibited miR130B may reduce cell viability, and increase cell death and the expression of apoptosis-related proteins, it also reduces the repression of TRIM3 by miR130B. Thus, it also results in the elevated expression of TRIM3 (*p*-value = 7.92 × 10^−40^), which helps EBV transport virions. MiR130B has been associated with apoptosis in viral-infected cells [66]. A brief overview of Fig 8B indicates that EBNA1-mediated PML silencing and disruption are responsible for inducing the successful progression of the EBV lytic cycle, which can promote virion production, vesicle trafficking, intracellular transport, and anti-apoptosis during lytic infection.

Fig 8C features EIF2AK2 plays essential roles in the innate immune response to viral infection, signal transduction regulation, apoptosis, and cell proliferation; it exerts its influence on anti-viral activity in a wide range of DNA and RNA viruses including EBV.

EIF2AK2, a serine/threonine protein kinase, is activated by autophosphorylation [67]. In Fig 8C, EIF2AK2 binds to the human receptor IL10RA, a receptor for interleukin 10 that is structurally associated with interferon receptors. IL10RA has been shown to mediate the immunosuppressive signal of interleukin 10, thereby suppressing the biosynthesis of pro-inflammatory cytokines. Hence, EIF2AK2 binds to IL10RA as a ligand. IL10RA is also a receptor of viral BCRF1, and induces anti-inflammatory factors to protect EBV proteins subjected to apoptotic attack by the immune system. *BCRF1*, a late gene of the lytic phase, encodes viral interleukin-10 (vIL-10), which is a human homolog of interleukin-10 (hIL-10). vIL-10 is expressed in both the early and late phases of viral lytic production when human B cells are infected with EBV. BCRF1 has certain cellular functions that are similar to those of hIL-10 proteins, which generally participate in immunosuppression. Highly expressed viral BCRF1 (*p*-value = 1.97 × 10^−5^) can suppress the cytokine synthesis of interleukins and interferon, and weaken the human natural killer cell and cytotoxic T-cell (CTL) responses so that EBV can subsequently establish latent infection. It can be supported that BCRF1 may have a role in the interaction between EBV and the human immune system [68, 69].

As shown in Fig 8C, BCRF1 interacts with viral BPLF1, a large tegument protein (LTP) that plays numerous roles in the EBV viral cycle. During EBV lysis, highly active BPLF1 (*p*-value = 3.5 × 10^−101^) remains associated with the capsid—whereas most of the tegument is detached—and has a role in the transport of the capsid toward the human membrane. As mentioned above, BPLF1 interacts with BALF3 and then BORF1 at the first infection stage. BALF3 interacts with BORF1 at the second infection stage, which mainly helps EBV terminate some final steps of viral replication during lytic infection, including DNA cleavage, DNA packaging, assembly of the capsid and tegument, and intracellular transport at the human membrane in preparation for virion lysis. Viral BVRF1 acts as a checkpoint in the formation between the viral capsid and the tegument. BVRF is a capsid vertex-specific component that is involved in EBV DNA encapsidation, ensuring an accurate DNA genome cleavage and stabilizing capsids. Moreover, viral BVRF1 can negatively interact with human PSMA3 to avoid the degradation of EBV proteins, so PSMA3 expresses with low activity (*p*-value = 1.28 × 10^−20^). The interaction has also been proposed in HIV-infected cells [15].

In Fig 8C, PSMA3 that is expressed at a low level positively interacts with the human TF STAT3 (also at a low level of expression) (*p*-value = 8.98 × 10^−13^) via ST3GAL3, and STAT3 is subjected to ubiquitination by ubiquitin protein USP33 so that human STAT3 has low activity (*p*-value = 1.35 × 10^−23^) with regard to reducing the suppression of lytic infection and decreasing the transcriptional inhibition of the human target gene *NRP1*. Human NRP1 is highly active (*p*-value = 1 × 10^−80^) and is able to transport and assist in EBV virion translocation. STAT3 has also been associated with transportation in viral-infected cells [70].

LRRK2 acts as a ligand and is largely present in the cytoplasm, but is also associated with the mitochondrial outer membrane, and positively regulates autophagy through a calcium-dependent signaling pathway [71]. In Fig 8C, it binds to a human receptor, NRP1, which contains some specific protein domains that allows it to participate in various types of signaling pathways that control cell migration, cell survival, transport, and attraction. The blocking of autophagy by EBV at the first infection stage causes the inactivation of LRRK2. The inactivated LRRK2 negatively interacts with its receptor, NRP1, and activates the transport function of NRP1. NRP1 transmits a signal to the human TF RUNX2 through positive interaction with BDH1, and subsequently VCAM1, both of which have high activity (*p*-value = 8.83 × 10^−70^ and *p*-value = 1.04 × 10^−39^, respectively). However, human VCAM1 negatively interacts with RUNX2, which leads to the low expression of RUNX2 (*p*-value = 5.62 × 10^−41^), thereby decreasing the transcriptional regulation of the human target gene *AFG3L1P*. AFG3L1P is a long non-coding RNA, and is associated with mitochondrial fusion and the import of proteins into the mitochondrial intermembrane space, which may trigger apoptosis. Thus, the low expression of AFG3L1P (*p*-value = 3.34 × 10^−8^) is due to the reduction of transcriptional regulation by RUNX2 and the repression of EBV miR-BART14, so that human AFG3L1P is unable to trigger apoptosis to defeat viral proteins owing to the indirect influence of blocked autophagy by EBV and the direct effect of the inhibition by EBV miR-BART14. RUNX2 has also been associated with apoptosis in HBV-infected B cells [37].

In Fig 8C, the low-level expression of lncRNA *AFG3L1P* results in a decrease of transcriptional inhibition of the human target gene *CLIC5*, a member of the chloride intracellular channel (CLIC) family of chloride ion channels. CLIC5, encoded by a human target gene, is related to actin-based cytoskeletal structures; it is inserted into membranes and forms poorly selective ion channels that may also transport chloride ions. Hence, high activity CLIC5 (*p*-value = 7.32 × 10^−5^) influences the activity and formation of chloride channels to enhance transport.

As shown in Fig 8C, human proteins BAX and PCBP2 interact with VCAM1 and ST3GAL3, respectively. Normally, the activation, conformational change, and relocation of BAX from the cytosol to the mitochondria cause the release of cytochrome C to the cytosol and trigger apoptosis, which can function as an offensive mechanism against EBV lytic infection [72]. BAX interacts with PCBP2; PCBP2 can regulate the susceptibility to lytic cycle activation signals and interact with STAT3 via ST3GAL3, which can mediate the repression of the EBV lytic cycle and maintain EBV latency [73]. However, BAX is degraded via ubiquitination by the ubiquitin-conjugating enzyme UBE2C, which results in a reduction of the apoptotic effect on EBV proteins. PCBP2 is also subjected to ubiquitination by UBE2C, repression of human miR421 and EBV miR-BART10, and DNA methylation (*p*-value = 1.07 × 10^−4^), all of which reduce PCBP2 expression (*p*-value = 5.58 × 10^−70^) and decrease lytic cycle repression, contributing to EBV lytic production and transportation. EBV miR-BART10 counteracts the expression of low activity human miR421 (*p*-value = 2.59 × 10^−5^), and has an effect on the inhibition of apoptosis. It can be supported that Bax has been associated with EBV lytic cycle gene, such as *BALF1* [74]. To summarize Fig 8C, viral BCRF1 performs the immunosuppressive mechanism that directly assists EBV in the final processes of virion production, and indirectly influences the transport of viral particles, the performance of anti-apoptotic function via EBV miR-BART14 and miR-BART10, the persistence of the lytic production cycle, and the progression of transportation by EBV miR-BART10. EBV maintains the transportation of viral particles by decreasing the repression of the lytic cycle and increasing anti-apoptosis activities.

### Overview of the molecular mechanism of lytic infection from the first to the second infection stages in human B cells infected with EBV

The suppressed expression of EBV latent antigens is important because it allows EBV-related tumors to evade immune surveillance. EBV lytic production is triggered by a variety of inducer treatments, and the induced lytic genes lead to cytotoxic T lymphocyte (CTL) responses [75]. Once the resting memory B cells latently infected with EBV are reactivated and enter the lytic phase, most of the EBV lytic genes and a few of the latent genes are transcribed and expressed in the lytic phase, and the human immune system simultaneously detects EBV antigens. The human immune response is thereby triggered. The response involves antigen-presenting cells, cytotoxic T cells, pro-inflammatory cytokines, reactive oxygen species, and certain pro-apoptotic signals that mediate immune mechanisms, including pro-apoptosis, autophagy, the inflammatory response, the human intrinsic immune pathway, and the extrinsic immune pathway in response to human B cells infected with EBV in the lytic phase.

**Fig 9:**
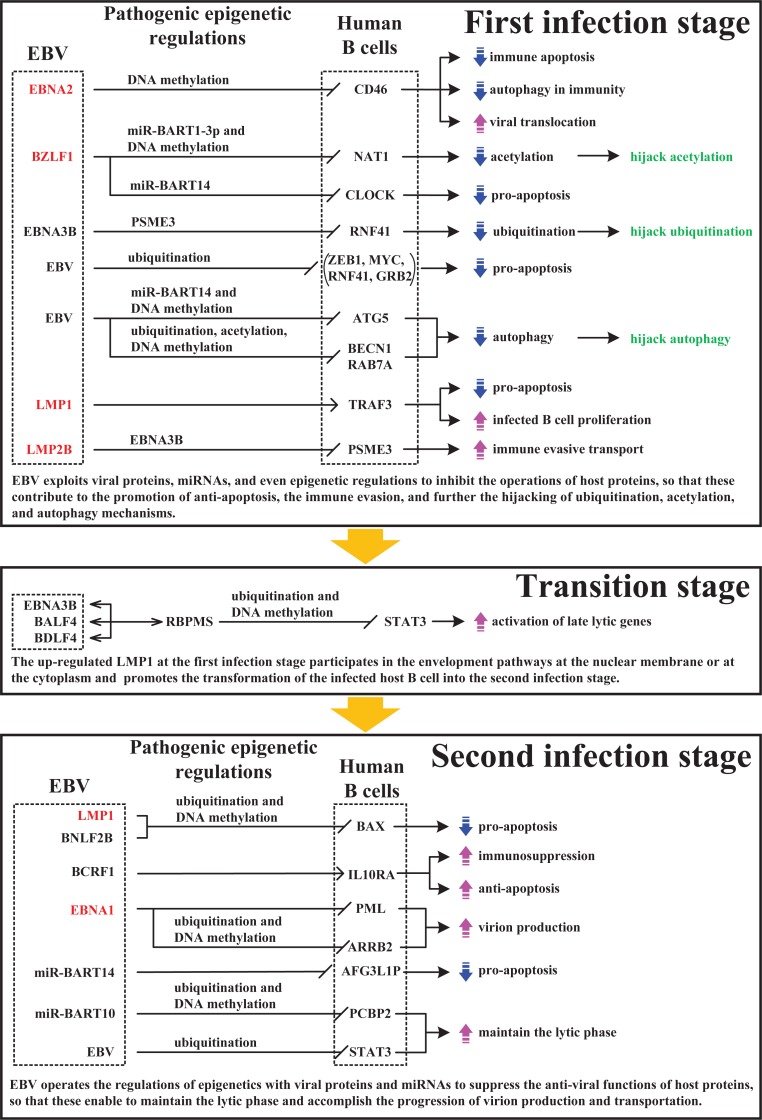
Overview of molecular mechanisms in EBV lytic infection and the significant network marker for potential multi-molecule drug design. The red words indicate the potential drug target proteins for multi-molecule drug design; the green words represent the molecular mechanisms being hijacked by EBV; the blue and pink arrows denote the cellular functions being inhibited or promoted, respectively; the yellow arrows represent the progression from the first into the second infection stage in the lytic phase. Upon EBV reactivation into the lytic phase, the human immune system can detect EBV antigens, trigger the human immune responses, and inhibit the progression of EBV lytic replication. However, EBV mediates some defensive mechanisms against human immune response, and thereby protects the complete virion production and transportation from human immune interference at both infection stages. Additionally, the transition stage of EBV is dependent on the activation of late lytic genes and LMP1 function.

As shown in Fig 9, EBV mediates certain defensive mechanisms against the human immune response at the first infection stage. It has been reported that EBNA2 and Notch intracellular domain (NICD) are partially interchangeable [76]. The nuclear-cytoplasmic transport of EBNA2 [77] could enable its association with Notch extracellular domain (NECD) to mediate the interaction between EBNA2 and CD46. In Fig 9, the viral protein EBNA2 evades immune apoptosis by interacting with human receptor CD46 with its immune inhibition property to induce an immunosuppressive phenotype, and then reduces the operation of human autophagy in the immunity of CD46 and the interruption of viral translocation of SNHG5. Viral IE protein Zta interacts with EBNA2 [15], transcriptionally activates EBV early lytic genes, inhibits human acetylation of NAT1 with viral miR-BART1-3p, and suppresses the human energy metabolism of CLOCK, which may trigger apoptosis, with viral miR-BART14. Furthermore, *BZLF1* itself has an anti-inflammatory response to the human immune system. Viral *miR-BART5* has an anti-apoptotic response that protects EBV from human immune attacks. Moreover, the viral miRNA miR-BART1-3p plays a role in anti-apoptosis, and may hijack the acetylation function of NAT1 by repressing the *NAT1* gene, so that EBV exploits other acetyltransferases to perform the acetylation at both infection stages. In addition, viral protein EBNA3B may hijack the ubiquitination function of RNF41 by negatively interacting with human protein PSME3, so that EBV exploits other ubiquitin proteins to carry out ubiquitination at both infection stages. EBV decreases the transcriptional regulation of human TFs (ZEB1 and MYC) and the expression of human receptors (RNF41 and GRB2) via ubiquitination to indirectly inhibit the human pro-apoptotic function of MYC and reduce the release of mitochondrial products of SLC25A6 that can trigger pro-apoptosis. Thus, EBV can block the autophagy mechanism through the indirect inhibition of autophagy-associated protein ATG5 with viral miR-BART14, and through acetylation and ubiquitination, to directly and indirectly restrict the other autophagy-related proteins (BECN1 and RAB7A). The viral proteins EBNA3B, BALF4, and BDLF4 hijack the autophagy-related autophagosomes, which facilitate intracellular vesicle trafficking. Moreover, EBV reduces the pro-apoptosis and viral release of CHMP5 and the translocational disruption of SNHG5, and increases the anti-apoptotic function of TGFB1I1 and PTK7 through the ubiquitination of CHMP5 and the suppression of human intrinsic defenses by viral protein BNRF1, which also enhances the activation and transcription of early viral genes. The anti-apoptotic signal induces viral protein BNLF2A to evade HLA class I-restricted T cell immunity and prevent TAP-mediated peptide transport and the subsequent loading. In addition, the degradation of viral protein BLLF1 via ubiquitination can protect viral replication from the aggression and interference of uninfected human B cells.

Under the protection of the defensive mechanism of EBV proteins and miRNAs mentioned above, EBV can securely produce viral particles without disturbance at the first infection stage. Viral protein EBNA2 can also promote infected cell proliferation. Viral protein BHRF1 prevents the premature death of the human cells during virus production. Viral protein BFRF3 then participates in the assembly of the infectious particles by decorating the outer surface of the capsid shell, thereby forming a layer between the capsid and the tegument. BFRF3 interacts with BCLF1, which self-assembles to form an icosahedral capsid. Viral protein BDLF4 is important for the EBV lytic replication cycle, and collaborates with BALF4 and EBNA3B so that they promote viral production and the intracellular transportation of virions. Membrane fusion is mediated by BALF4, and the heterodimer BXLF2/BKRF2 may also be involved in the fusion between the virion envelope and the outer nuclear membrane during virion morphogenesis. LMP1 acts as a CD40 functional homolog to prevent the apoptosis of the infected B cells to drive their proliferation. LMP1 signaling leads to the upregulation of anti-apoptotic proteins and provides cell growth signals in the infected cells. BFLF2 plays a fundamental role in EBV virion nuclear egress. Viral receptor BBRF3, an essential lytic replication protein, is an envelope glycoprotein and is crucial for virion assembly and egress. BDRF1 can translocate viral genomic DNA into the empty capsid during DNA packaging, and BDRF1 forms a tripartite terminase complex together with BALF3 and TRM2 in the human cytoplasm. The viral icosahedral capsid is composed of pentamers and hexamers of the major capsid protein, which are linked together by triplexes. These triplexes consist of BORF1 and BDLF1. Additionally, BORF1 is required for the efficient transport of BDLF1 to the nucleus, which is the site of capsid assembly. Viral BALF3 functions with BDRF1 and TRM2, and they collaboratively translocate EBV genomic DNA into the empty capsid during DNA packaging. Viral membrane proteins LMP2B and LMP1 collaboratively activate human B cells by interacting with BLLF2, and both of them work in concert with EBNA3B so that EBNA3B can mediate the immune evasive transport of complete virions via autophagic vesicles.

RBPMS, interacting with viral proteins EBNA3B, BALF4, and BDLF4, acts as the latent–lytic switch in EBV by negatively interacting with STAT3. Inactivated STAT3 cannot transcriptionally activate cellular PCBP2, and PCBP2 cannot repress the expression of EBV lytic genes. This increases the transcriptional activation of EBV late lytic genes, which may contribute to the complete progression of the EBV lytic production cycle into the late stage. A viral membrane protein existing in both infection stages may play a crucial role in transformation during the lytic phase of viral production. EBV utilizes the significantly different expression levels of LMP1 for two purposes during its life cycle. First, in the latent infection of B cells, the steady-state expression of LMP1 is important for maintaining the transformational state. Second, in the EBV lytic production cycle, the induced LMP1 efficiently facilitates the release of the virions from the B cells. Once EBV lytic replication has been induced, the expression of LMP1 is upregulated. Here, we indicated that the gene products of LMP1 are responsible for the efficient release of the virus from the B cells. It is feasible that viral LMP1 enhances the envelopment, de-envelopment, and re-envelopment pathways either at the nuclear membrane or in the cytoplasm during the lytic phase [78].

Viral membrane protein LMP1 promotes the transformation of human B cells infected with EBV from the first into second infection stage, and protects the infected B cells from apoptosis. EBV maintains the defensive mechanism to protect the complete virion production and transportation from human immune interference. Viral proteins (LMP1 and BNLF2B) indirectly reduce the pro-apoptotic function of BAX. BNLF2B is very similar to BCRF1, which may facilitate immunosuppression to protect EBV. EBV increases the anti-apoptotic performance of viral miR-BART1-3p and the immunosuppression of viral protein BCRF1 as an immune evasive mechanism through the degradation of HNRNPU via ubiquitination. Viral antigen EBNA1 can induce anti-apoptosis to mediate the disruption and silencing of PML and block the pro-apoptotic signal. The pro-apoptosis of ARRB2 is inhibited by the ubiquitination and indirect suppression of EBNA1. Viral miR-BART14 can repress the expression of AFG3L1P, which triggers pro-apoptosis. Viral miR-BART10 exploits the anti-apoptotic response to antagonize human pro-apoptosis, and represses the expression of PCBP2 to protect virion production in the lytic cycle from restriction. EBV also exploits the effects of ubiquitination to degrade BAX, which has a role in pro-apoptosis, and to reduce the expression of proteins STAT3 and PCBP2, which participate in the transformation from the lytic phase to the latent phase.

EBV promotes transport to maintain the production of viral particles. Viral BNLF2B and miR-BART1-3p exploit PRKACB to promote envelope assembly and the intracellular transport of virions. EBV promotes tRNA splicing of FAM98B through the degradation of TF NFATC2 via ubiquitination to increase the expression of late lytic genes and the genetic diversity in cell packaging, assembly, and cell transport. The activated EBNA1 can assist virion production and indirectly promote cell transportation of TRIM3. Viral miR-BART14 can assist CLIC5 in cell transport by repressing the expression of AFG3L1P, and EBV can help NRP1 activate cell transport through the degradation of STAT3 via ubiquitination. Viral BPLF1 remains associated with the capsid—whereas most of the tegument is detached—and has a role in the transport of the capsid toward the plasma membrane. BALF3 interacts with BORF1 at the second infection stage, which mainly helps EBV terminate some final steps of viral production during lytic infection. Viral BVRF1 acts as a checkpoint at the formation between the viral capsid and the tegument, and is involved in EBV DNA encapsidation, maintaining an accurate DNA genome cleavage to stabilize the capsids. These factors suggest that EBV exploits viral proteins and miRNAs to develop its defensive mechanism to defeat multiple immune attacks by the human immune system, promotes virion production via viral proteins, and facilitates the transportation of viral particles by activating the expression of certain human genes. A better understanding of host–virus cross-talk interactions could help in the design of new therapeutic drugs against EBV-associated malignancies.

### Network parameter-based pathway enrichment analysis and validation of HVCNs

In order to validate our results, we applied the well-proposed analysis method to the two-sided time-course expression data to discover the top lytic-cycle genes at the first and second infection stages. In 2012, coexpression analysis has been applied to the expressions of EBV lytic genes and human host genes across 201 RNA-seq experiments to identify the potential human genes, which were upregulated by EBV lytic genes during lytic reactivation [79]. In this study, we applied coexpression analysis to the two-sided time-course expression data during the EBV infection to identify the top human genes (top 500 genes), positively related with first stage lytic cycle genes (upper two panels in Fig 2) or second stage lytic cycle genes (lower panel in Fig 2). By comparing top genes at first and second infection stages with the identified 378 and 389 human core genes/proteins /receptors/TFs in HVCNs (in Figs 5 and 6, respectively) at the first and second infection stages during the lytic phase, respectively, the result shows 3.6% and 2.2% consistency at the two stages, respectively. It can be supported that only about 2% of the coexpressed genes have related cellular functions [80]. Recently, there is still no genome-wide approach to discover the potential first and second stage lytic cycle genes during the lytic phase.

In order to validate the network functions of the identified HVCNs based on the network parameters, we applied network parameter-based pathway enrichment analysis [81] to the interaction parameters $\alpha_{in}^{\left(h\right)}$ in (1) of the core human PPIs. The top five pathways and their corresponding genes at the first and second infection stages are shown in Table 5 (full table shown in S1D Table).

**Table 5:** Network parameter-based pathway enrichment analysis of human core PPIs in HVCPs. The result is sorted by the number of genes involved in the pathways. The full table is shown in S1D Table.

First infection stage			Second infection stage		
Enriched Gene Sets of HVCP interacting with EBV proteins	z-score	Genes	Enriched Gene Sets of HVCP interacting with EBV proteins	z-score	Genes
NABA_MATRISOME_ASSOCIATED	1.887	BMP1, CLEC2D, CTSC, FGF2, FREM1, GH1, IGF1, PLAU, S100A8, S100A9, SEMA4G	REACTOME_IMMUNE_SYSTEM	5.172	CD40LG, EIF2AK2, FBXO4, FOXO3, HCK, IKBKB, IL6ST, IRAK4, MALT1, MAP3K14, MAPK1, MAPKAP1, NUP155, PCBP2, PIK3R3, PML, PRKACB, PSMA3, PTPN6, STAT3, UBE2C, UBE3A, VCAM1
KEGG_P53_SIGNALING_PATHWAY	2.318	BAX, CDKN2A, CHEK1, IGF1, RCHY1, SIAH1, TNFRSF10B, TP73	REACTOME_SIGNALING_BY_GPCR	5.028	CCL19, CCL3L1, CXCL13, EGFR, GNB5, GNG5, GPBAR1, GPR65, MAPK1, MCHR1, NPFFR2, OR10A6, OR3A2, OR9A2, PDE1B, PIK3R3, PRKACB, PTGER1
BIOCARTA_HIVNEF_PATHWAY	3.010	DAXX, MAPK8, NFKB1, RELA, TRAF1, XIAP	REACTOME_ADAPTIVE_IMMUNE_SYSTEM	5.497	CD40LG, FBXO4, FOXO3, IKBKB, MALT1, MAP3K14, MAPKAP1, PIK3R3, PRKACB, PSMA3, PTPN6, UBE2C, UBE3A, VCAM1
BIOCARTA_CBL_PATHWAY	1.789	GRB2, PRKCB, SH3GLB1, SH3GLB2	REACTOME_DEVELOPMENTAL_BIOLOGY	5.497	ALCAM, EGFR, FOXO3, GFRA2, KIAA1598, MAPK1, NCOA1, NRP1, RDX, SMAD2, TCF4
PID_ER_NONGENOMIC_PATHWAY	1.768	ESR1, ESR2, GRB2, IGF1	KEGG_NEUROTROPHIN_SIGNALING_PATHWAY	3.643	ARHGDIB, BAX, FOXO3, IKBKB, IRAK4, MAPK1, PIK3R3, TP73

At the first infection stage, it has been reported that the tightly controlled ECM homeostasis is essential for life-threatening pathological conditions in B cells, resulted from the sustained dysregulation [82]. p53 has been known to be essential for the regulation of miRNAs and activation of histone deacetylase (HDAC) inhibitor (HDACi)-induced early EBV lytic infection in B cell lines [83, 84]. Like HIV Nef protein, EBV proteins BHRF1 and BZLF1 have been reported to protect EBV-infected cells from cell death by inhibiting the expression of *TNFR1* or *FAS* at the first infection stage during the lytic phase [85, 86]. Since it has been previously shown that Cbl is a negative regulator of early EBV lytic induction and promotes the degradation of LMP2A and LMP2A associated proteins [87], we suggested that the induced CBL pathway ensures the progression of the first lytic infection to the second infection stage in EBV-infected B cells. The activation of early lytic cycle is associated with the dynamic interaction between human autoimmunity and EBV proteins through estrogen signaling pathway [88].

At the second infection stage, it has been proposed that late lytic gene products, such as *BCRF1* [89], and G-protein-coupled receptor (GPCR) pathway [90] are involved in immune evasion and adaptive immune evasion mechanisms, which lead to high-level resistance of late-lytically infected B cells to nature killer cell killing [91]. In EBV-infected B cells, late lytic proteins, such as BGLF2, are found to activate AP1-p38 MAPK signaling pathway [92, 93], which is associated with cell growth, apoptosis, and differentiation. Neurotrophin and NGF signaling pathways are thought to contribute to non-Hodgkin [94] and Hodgkin lymphomas [95], respectively, which are highly associated with the patients with EBV infection. Late lytic genes, such as *BPLF1*, were found to help drive B cell immortalization and increase the incidence of lymphomagenesis [96]. Therefore, the results in HVCNs at the first and second infection stages were supported.

### Drug target proteins and multi-molecule drug design

In this section we will explore potential drug target proteins and multi-molecule drug design, aimed at human B cells infected with the EBV lytic phase. EBV is a γ- herpesvirus that has dual roles in its life cycle. One is its latent infestation of human cells with only some latent genes being expressed, and the other is reactivated lytic infection, which contributes to new virion production and transportation. Because most viral proteins are expressed during the lytic phase, EBV has developed defensive mechanisms to antagonize the human immune system. Therefore, our therapeutic strategies are aimed at applying multi-molecule drugs to the cells infected with EBV for the purpose of blocking reactivation to the lytic phase, interrupting the viral production of virions, interfering with the transportation of viral particles, and destroying viral defensive mechanisms.

The reactivation of EBV to the lytic phase provides an opportunity to promote EBV-dependent viral cell killing; the method, called induced lytic therapy, requires drugs and other agents that can induce EBV reactivation without causing unacceptable cytotoxic substances to form in normal cells. Current induced lytic strategies use a protein kinase (PK) encoded by the EBV early lytic gene *BGLF4*, which can transform the nucleoside analog ganciclovir (GCV) into cytotoxic drugs that can promote apoptosis and kill viral cells. Phosphorylated GCV can also be transferred to the adjacent cells via gap junctions, so the activation of ganciclovir phosphorylation could result in the “bystander killing” of a number of viral cells and even normal cells. Ganciclovir, the classic anti-viral drug, can suppress the activity of viral cell DNA polymerase and eradicate the virus-infected cells. However, ganciclovir in the phosphorylation form can lead to much greater cell death due to bystander killing, and can also inhibit the activity of human cell DNA polymerase. Moreover, ganciclovir is only effective against EBV lytic infected cells. Because EBV-positive tumor cells are mainly in the EBV latent infection phase, ganciclovir is not useful for treating EBV-positive tumors by itself [97, 98].

We considered viral proteins EBNA2 and Zta to have the primary role in the initiation of the EBV lytic phase. We forecast EBNA2 and Zta as the potential drug targets in the progression of the EBV lytic phase. EBNA2 can efficiently upregulate the genes involved in infected cell proliferation and survival, and it can evade human immune attacks by interacting with CD46, as shown in Fig 8A. Moreover, the switch between the latent phase and the lytic phase of EBV infection can be induced by the expression of the immediate–early gene product Zta, which is a transcription factor and is capable of inducing the entire program of EBV lytic gene expression [99, 100]. We also suggested that EBV membrane proteins LMP1 and LMP2B, and EBV nuclear antigen EBNA1 are potential drug targets, because they not only participate in reactivation from the latent phase to the lytic phase, but also in the defensive mechanisms of virion production at both infection stages, as shown in Figs 5 and 6, respectively. LMP2B works in cooperation with LMP1 via viral BLLF2 to enhance the activity of human B cells and facilitate the production and transportation of viral particles. In addition, among all EBV-encoded proteins, LMP1 performs the function of anti-apoptosis at both infection stages during the lytic phase, and plays a central role in the propagation of EBV-associated lymphoma. The conventional treatment for EBV-associated malignancies cannot prevent tumor metastasis, recurrence, and disease progression, so we considered that therapeutic strategies targeting EBV-encoded proteins may increase the cure rate and provide a clinical benefit [101]. Viral protein EBNA1, another prime target for therapeutic intervention, acts as the major switch that regulates EBV gene activity and activates EBV dormancy in humans. EBNA1 is essential for the virus to reproduce via anti-apoptosis. Knocking out *EBNA1* could therefore destroy EBV and manipulate the growth of EBV-associated cancer [102].

**Fig 10:**
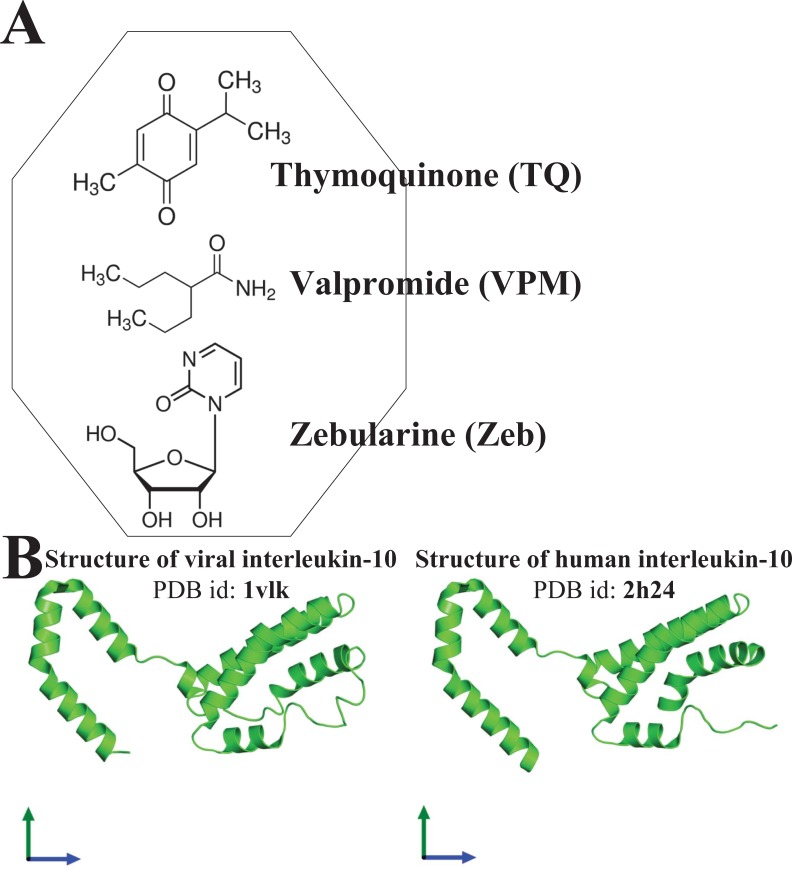
**(A) Multi-molecule drug design for the predicted drug targets; (B) Protein structures of EBV interleukin-10 and human interleukin-10.** Thymoquinone (TQ) inhibits the RNA expression of viral *EBNA2*, *LMP1*, and *EBNA1*; valpromide (VPM) can prevent the gene expression of viral *BZLF1*; zebularine (Zeb) can decrease the upregulation of viral *LMP2A*, *LMP2B*, and *EBNA2*. These drugs can block reactivation, interrupt the viral production of virions, interfere with the transportation of viral particles, and destroy viral defensive mechanisms.

To develop anti-EBV drugs and construct the drug databases for drugs to target EBV proteins, we began a complex screening process to discover a small molecule that could chemically bind to viral proteins and inhibit their abilities to perform. Thus, we carried out drug mining of the literature to design multi-molecule drugs that were appropriate for the potential drug targets. Ismail et al. carried out an *in vitro* investigation of the activity of the potent herbal extract drug thymoquinone in EBV [103]. Thymoquinone (TQ) was tested for cytotoxicity in human Burkitt’s lymphoma cells and certain other EBV-related lymphomas. Thymoquinone was found to efficiently inhibit the RNA expression of viral genes *EBNA2*, *LMP1*, and *EBNA1*. In particular, the optimal EBNA2 expression level indicated that EBNA2 might make the main contribution to thymoquinone potency against EBV-infected cells [103]. The researcher’s results suggest that thymoquinone has the potential to efficiently inhibit the growth of EBV-infected B cells. Valpromide (VPM), which is not an HDAC inhibitor, is another drug that can block EBV reactivation. VPM can prevent the gene expression of viral *BZLF1*, which mediates lytic reactivation. VPM cannot activate the expression of some cellular immediate–early genes including *FOS* and *EGR1*, which are upstream of the EBV lytic cycle, but it can reduce their activities. Thus, VPM can selectively suppress both viral and cellular gene expression. VPM represents a new class of antiviral agents that prevent the initiation of the EBV lytic phase. VPM will be useful in exploring the mechanism of EBV lytic reactivation and may have therapeutic potential [104]. Zebularine (Zeb) is a DNA methyltransferase inhibitor (DNMTi) that induces the expression of E-cadherin, which is encoded by a cellular gene that is frequently silenced by hypermethylation in cancers. Zebularine can decrease the upregulation of viral genes *LMP2A*, *LMP2B*, and *EBNA2* to prevent the switch from the latent phase to the lytic phase upon cross-linking with lytic inducers such as B-cell receptors. Zebularine could also be used to treat EBV-associated tumors, because it does not induce the switch from the latent phase to the lytic phase that may cause secondary EBV-related malignancies [105]. Consequently, we integrated these drugs as the potential multi-molecule drugs, as shown in Fig 10A, for the predicted drug targets. We considered that the multi-molecule drugs can inhibit the activities of viral proteins and thus play a potent role in counteracting the reactivation from latency to the lytic phase during the EBV lytic infection of human B cells. They are therefore suitable candidates for further development as inhibitors of EBV-associated malignancies.

Moreover, the hijack of epigenetic regulation by EBV is essential for the establishment of life-long latency and the facilitation of malignant B cell transformation. Understanding how specific epigenetic regulations promote the progression of lymphomas, autoimmunity, and EBV-associated cancers is fundamental to the design of novel therapeutic interventions for the cure of certain often fatal symptoms [106]. Thus, from the HVCPs in Figs 7 and 8, we can predict other drug targets comprising EBV proteins and the four viral miRNAs. Certain viral proteins (BALF4, EBNA3B, and BDLF4) may block the autophagy mechanism and hijack the autophagic vesicles for virion transportation; BNRF1 can inhibit human intrinsic defenses to enhance the activation and transcription of early viral lytic genes; BNLF2A can reduce the effect of antigen-presenting cells, and thereby evade T cell immune responses; BLLF2 mediates the interaction between EBV membrane proteins LMP1 and LMP2B, and may participate in the hijack of the autophagy mechanism; BNLF2B is similar to viral BCRF1, and BNLF2B and BCRF1 can perform the function of immune evasion to protect EBV from human immune surveillance. Therefore, novel multi-molecule drugs based on targets including EBV proteins and, particularly, EBV miRNAs, will interrupt the development of the EBV lytic phase and facilitate the rapid detection of viral lytic proteins.

Furthermore, EBV can exploit certain viral-encoded proteins that are homologues of human proteins to interact with human receptors with the objective of evading and even inhibiting the human immune response. We carried out analyses to determine the structural similarities between viral BCRF1 and human cellular IL10 using the Protein Data Bank in Europe (PDBe) database and its tool, PDBeFold. The structure alignment results show 94% amino acid sequence identity and 71% secondary structure identity between vIL-10 and hIL-10. As shown in Fig 10B, viral BCRF1 is a homologue of human IL10. It is a functionally critical cytokine regulator of immune tolerance, and interacts with human receptor IL10RA in the second stage of infection shown in Fig 6. This potentially crucial form of EBV immune modulation and immune suppression is due to EBV-encoded proteins called virokines, which are similar to human cytokines [107]. It may become very important to discover more interactions between EBV homologues and human proteins.

Another strategy is to induce the transformation from the latent form of infection to the lytic phase, thereby causing EBV-associated cell death. However, the period of latency in the EBV life cycle is much longer than the period of lytic reactivation and production, and the expression of latent EBV proteins is limited; these two aspects of latency may affect the accuracy and effectiveness of multi-molecule drugs. Drugs that affect the EBV latent phase can prevent the mutations in EBV-infected cells that cause EBV-associated malignancies, such as EBV-positive tumor cells, which are primarily initiated in the latent form of EBV infection.

## Conclusion

The World Health Organization (WHO) has defined EBV as a Class I carcinogen, and it is estimated to result in a small but significant portion of all human cancers. EBV may persist in the human body for decades and cause infected cells to become cancerous. It is estimated that EBV leads to nearly 400,000 cases of cancer each year, including Burkitt’s lymphoma, Hodgkin’s lymphoma, gastric carcinoma, and nasopharyngeal carcinoma. To persist in human B cells, EBV has evolved various strategies to manipulate the human immune response, including restricting immune cell functions, blocking apoptotic pathways, and interfering with antigen presentation functions. In this study, we investigated the pathogenic mechanisms by which dysregulations and dysfunctions of human B cells and immune modulation by EBV can contribute to the development of EBV lytic replication and production.

The reactivation of EBV-infected human B cells into the lytic phase initiates viral replication and infectious virion production. At the same time, a number of EBV lytic genes are successively activated and expressed, so the human immune system can detect and respond to infection-associated viral proteins. Viral nuclear antigen EBNA2 can impair human immune information by interacting with receptor CD46, which then promotes the proliferation of infected cells and increases the transcription of the viral immediate–early lytic gene *BZLF1*. Zta can activate early lytic genes and induce viral anti-apoptosis. EBV can exploit the epigenetic changes due to ubiquitination and acetylation to block the pro-apoptotic pathway via viral miR-BART1-3p, which can inhibit the expression of NAT1 and mediate human proteins and genes relevant to pro-apoptosis that are subjected to DNA methylation, acetylation, and ubiquitination. The human body’s natural response is to exploit the autophagy mechanism to eliminate EBV proteins. However, EBV blocks autophagy and hijacks the autophagic vesicles to facilitate viral transport. The human body uses CHMP5 and lncRNA SNHG5 to induce pro-apoptosis and interrupt viral translocation, respectively. The epigenetic effects of ubiquitination, deacetylation, and DNA methylation influence these pathways, and viral BNRF1 destroys the DAXX complex in the pro-apoptotic pathway. Thus, these host/virus mechanisms contribute to the complete progression from lytic production through to the impairment of pro-apoptosis and the promotion of viral translocation and anti-apoptosis. In the first stage of infection, EBV protects the infected human B cells from elimination by autophagy and keeps the infected B cells in the lytic phase until the integrated production and transportation of new infectious virions is complete. Thus, EBV inhibits and hijacks the function of autophagy via BECN1, and silences the expression of STAT3, which can repress the expression and activity of lytic genes upon STAT3 in the activated status. These defensive mechanisms of EBV result in the persistence of lytic infection and the promotion of viral production. Viral membrane proteins LMP1, BNLF2B, and miR-BART1-3p can perform the function of anti-apoptosis, and viral BCRF1 enables immunosuppression by releasing viral IL10 to escape immune surveillance. Furthermore, EBV exploits transcriptionally activated FAM98B, which can increase tRNA splicing, thereby creating more genetic diversity in the proteins involved in the packaging, assembly, and transportation of new viral particles. The disruption of PML by viral EBNA1 contributes to the inhibition of pro-apoptosis. EBNA1 has the ability to promote virion production, and indirectly influences vesicle trafficking and virion release by TRIM3 and viral membrane protein LMP1. It is necessary to maintain virion transportation by decreasing the repression of the lytic cycle and increasing anti-apoptosis. The epigenetic modifications of ubiquitination and DNA methylation, and the repression of PCBP2 by viral miR-BART10 decrease the ability of PCBP2 to inhibit the lytic phase, and viral miR-BART14 represses the expression of lncRNA AFG3L1P, which can trigger pro-apoptosis. EBV indirectly exploits the expressed NRP1 and CLIC5 to promote the transportation of new virions.

Real GIGENs, HVCNs, and HVCPs obtained through dynamic system models, big database mining, and NGS data provide a new perspective. Compared with literature reviews, the approach reported here provides more information on interspecies and intraspecies protein–protein interactions and signaling transduction pathways from receptors to TFs. It also provides information on the transcription regulation of target genes in the context of epigenetic miRNAs and lncRNAs regulation. Here, we propose a multi-molecule drug design based on our exploration of the EBV drug target proteins from HVCNs. These multi-molecule drugs (Fig 10A) can inhibit reactivation from the latent form to the lytic form in the EBV life cycle. They can also reduce the abilities of some critical EBV lytic genes/proteins, which can reactivate the lytic phase, the viral production of virions, the transportation of viral particles, and EBV defensive mechanisms during lytic infection. In the future, we hope to discover and collect more information about lncRNAs and open reading frames (ORF), and predict links between humans and EBV to improve on the models and results reported in this study. Thus, it may be possible to discover other novel cross-talk mechanisms between humans and EBV, as well as further EBV pathogenic mechanisms occurring during lytic reactivation, lytic infection, and even the latent phase and its associated carcinogenesis.

## Materials and methods

### Overview of the construction for interspecies GIGENs in human B cells infected with EBV during the lytic production phase

A flow chart of the progression for constructing the GIGENs, the HVCNs, and the HVCPs in human B cells infected with EBV at the first and second infection stages in the lytic phase is shown in Fig 1. The GIGENs were composed of human/EBV gene/miRNA/lncRNA regulatory networks (GRNs), human/EBV protein–protein interaction networks (PPINs), the interspecies PPINs, and the interspecies GRNs. The GIGENs, the HVCNs, and the HVCPs were constructed in the following steps: (1) big data mining and data preprocessing to establish the candidate GIGEN; (2) identification of the real GIGENs by pruning false positives from the candidate GIGEN using the system identification approach and the system order detection scheme with the genome-wide NGS data for human B cells and EBV at the first and second stages during the EBV lytic reactivated infection; and (3) extraction of the HVCNs by applying the principal network projection (PNP) method to the real GIGENs at the first and second infection stages. These procedures were used to identify the crucial and specific interspecies mechanisms at both infection stages during the EBV lytic phase.

### Big data mining and data preprocessing of NGS data for humans and EBV, and DNA methylation profiles for humans

NGS datasets were obtained from the Gene Expression Omnibus (GEO) at the National Center for Biotechnology Information (NCBI). A study by Tina O'Grady et al. demonstrated that EBV reactivation includes the ordered induction of approximately 90 viral genes that are involved in the production of infectious virions[7]. They found extensive bidirectional transcription stretching across nearly the entire genome, and estimated that probably hundreds more EBV genes are expressed during EBV reactivation than was previously thought. They also suggested that the viral genome during EBV reactivation might be much more complex than had been suspected, and changed our view of the virion production process during the EBV lytic phase. We obtained the NGS data from this study containing both the human (hg19 assembly) and the Akata EBV genomes with the time course through the GEO series with accession number GSE52490[7]. The raw data of the NGS dataset comprises two parts. One part involves the gene expression profiles of human B cells with EBV lytic infection at 0, 1/12, 0.5, 1, 2, 4, 8, 24, and 48 hours post reactivation. The other part involves the gene expression profiles of EBV in the lytic phase at 0, 1/12, 0.5, 1, 2, 4, 8, 24, and 48 hours post reactivation. It contains 44,446 human probes and 134 EBV probes. According to the gene expression profiles of the viral IE lytic genes *BZLF1* and *BRLF1* (Fig 2, top panel), the early lytic genes *BMRF1*, *BBLF3*, *BBLF4*, *BGLF5*, *BNLF2A* and *BSLF1* (Fig 2, middle panel) and the late viral genes *BCRF1*, *BVRF2*, *BDLF1*, *BLLF1* and *BCLF1* (Fig 2, bottom panel) during the infection, we classified the lytic phase into the first infection stage from 0 to 24 hours, where the IE lytic genes and the early lytic genes are highly expressed, and the second infection stage from 8 to 72 hours, where the early lytic genes and the late viral genes are highly expressed. We subsequently applied analysis of variance (ANOVA) to the NGS data for human and EBV mRNA expression to evaluate the *p*-value for the differential expression data for the first and second infection stages.

The candidate GIGEN was constructed through big data mining from numerous databases that contain many experimental data and bioinformatic (computational) predictions. The human candidate PPIN was obtained from BioGRID[108], DIP[109], BIND[110], IntAct[111], and VirusMINT[112]. The human candidate GRN comprised transcription factors (TFs)/ TF complex-regulating genes, lncRNA-regulating genes, and miRNA-repressing genes, which were available at HTRIdb[113], ITFP[114], TargetScan (http://www.targetscan.org/), and CircuitsDB 2[115]. The interspecies candidate PPIN and EBV candidate PPIN required interspecies and intraspecies interactions, which were obtained from VirusMentha[116], CDFD, (http://www.cdfd.org.in/labpages/computational_biology.html), Virhostome[117], IMEx[118], and PSICQUIC[119]. The interspecies candidate GRN and EBV candidate GRN involved interspecies and intraspecies TF-regulating genes and miRNA-repressing genes that were collected from VIRmiRNA[120], ViRBase[121], miRecords[122], starBase v2.0[123], and miRTarBase[124].

To support the inference of human target genes that are subjected to epigenetic regulation of DNA methylation based on the results of system identification, we exploited the genome-wide DNA methylation profiles of B cells and immortalized B cells (GSE41957)[125] that were uninfected and infected with EBV, respectively (with a sample size of 6). We applied the ANOVA statistics to these DNA methylation data.

As a result, in the intraspecies candidate PPIN, we obtained 301 EBV PPI pairs and 23,570,918 human PPI pairs; in the interspecies candidate PPIN, we obtained 5,135 human-EBV PPI pairs. In the intraspecies candidate GRN, we obtained 5 EBV TF-gene pairs, 67 EBV miRNA-gene pairs, 906,611 human TF-gene pairs, 817,900 human miRNA-gene pairs, and 1,948 human lncRNA-gene pairs; in the interspecies candidate GRN, we obtained 1,252 EBV TF-human gene pairs, 39,772 EBV miRNA-human gene pairs, 1,355 human TF-EBV gene pairs, and 1,718 human miRNA-EBV gene pairs. Among the intraspecies human candidate GRNs there are three human TF complexes. The first is ARNT::AHR, which has 6,368 human TF-gene pairs; the second is HIF1A::ARNT, which has 1,011 human TF-gene pairs; and the third is NFE2L1::MAFG, which has 5,787 human TF-gene pairs. In conclusion, we built a candidate GIGEN comprising the various candidate pairs mentioned above, and we then detected the real GIGENs by pruning the false positives from the corresponding candidate GIGEN via the system identification approach and the system order detection scheme using the genome-wide NGS data for human B cells and EBV at both stages of infection during the EBV lytic phase.

### Dynamic models of the GIGEN for human B cells and EBV during the lytic infection process

The candidate GIGEN comprised the experimental and computational predictions, which would have resulted in a number of false-positive interactions and regulations. It was, therefore, necessary to prune the false positives of the candidate GIGEN to construct real GIGENs using the genome-wide NGS data at the first and second infection stages for human B cells and EBV through the system identification approach and the system order detection scheme. We then extracted the core GIGENs using the PNP scheme to characterize the principal biological mechanisms of the GIGENs.

The PPI of human-protein *i* in the candidate PPIN can be described by the following stochastic dynamic equation:

$$\begin{array}{l} p_{i}^{\left(h\right)}(t+1)=p_{i}^{\left(h\right)}(t)+\sum_{n=1}^{N_{i}}\alpha_{in}^{\left(h\right)}p_{n}^{\left(h\right)}(t)p_{i}^{\left(h\right)}(t)+\sum_{j=1}^{J_{i}}\gamma_{ij}^{\left(h\right)}p_{j}^{\left(v\right)}(t)p_{i}^{\left(h\right)}(t)\\ \;-\sigma_{i}^{\left(h\right)}p_{i}^{\left(h\right)}(t)+\lambda_{i}^{\left(h\right)}g_{i}^{\left(h\right)}(t)+\beta_{i}^{\left(h\right)}+\varepsilon_{i}^{\left(h\right)}(t),\\ \;\mathrm{for}\;i=1,2,\ldots ,I,-\sigma_{i}^{\left(h\right)}\leq 0,\;\mathrm{and}\;\lambda_{i}^{\left(h\right)}\geq 0 \end{array}$$ (1)

where $p_{i}^{\left(h\right)}\;(t)$, $p_{n}^{\left(h\right)}\;(t)$, $g_{i}^{\left(h\right)}\;(t)$, and $p_{j}^{\left(v\right)}\;(t)$ indicate the expression levels of human-protein *i*, human-protein *n*, human-gene *i*, and EBV-protein *j* at time *t*, respectively; $\alpha_{in}^{\left(h\right)}$ and $\gamma_{ij}^{\left(h\right)}$ represent the interactive abilities between human-protein *n* and human-protein *i*, and between EBV-protein *j* and human-protein *i*, respectively; $‑\sigma_{i}^{\left(h\right)}$, $\lambda_{i}^{\left(h\right)}$, and $\beta_{i}^{\left(h\right)}$ denote the degradation rate, the translation effect, and the basal level of human-protein *i*, respectively. The basal level $\beta_{i}^{\left(h\right)}$ denotes interactions with unknown factors, for example, acetylation and ubiquitination. *N*_*i*_ and *J*_*i*_ represent the numbers of human proteins and EBV proteins interacting with human-protein *i* in the candidate GIGEN, respectively; and $\varepsilon_{i}^{\left(h\right)}(t)$ is the stochastic noise of human-protein *i* owing to model uncertainty or other uncertain factors at time *t*. Note that the biological interaction mechanism of human proteins in (1) involves the intraspecies human PPIs represented by $\sum_{n=1}^{N_{i}}\alpha_{in}^{\left(h\right)}p_{n}^{\left(h\right)}(t)p_{i}^{\left(h\right)}(t)$, and the interspecies PPIs represented by $\sum_{j=1}^{J_{i}}\gamma_{ij}^{\left(h\right)}p_{j}^{\left(v\right)}(t)p_{i}^{\left(h\right)}(t)$.

The PPI of EBV-protein *j* in the candidate PPIN can be described by the following stochastic dynamic equation:

$$\begin{array}{l} p_{j}^{\left(v\right)}(t+1)=p_{j}^{\left(v\right)}(t)+\sum_{m=1}^{M_{j}}\alpha_{jm}^{\left(v\right)}p_{m}^{\left(v\right)}(t)p_{j}^{\left(v\right)}(t)+\sum_{i=1}^{I_{j}}\gamma_{ji}^{\left(v\right)}p_{i}^{\left(h\right)}(t)p_{j}^{\left(v\right)}(t)\\ \;-\sigma_{j}^{\left(v\right)}p_{j}^{\left(v\right)}(t)+\lambda_{j}^{\left(v\right)}g_{j}^{\left(v\right)}(t)+\beta_{j}^{\left(v\right)}+\varepsilon_{j}^{\left(v\right)}(t),\\ \;\mathrm{for}\;j=1,2,\ldots ,J,\;-\sigma_{j}^{\left(v\right)}\leq 0,\;\mathrm{and}\;\lambda_{j}^{\left(v\right)}\geq 0 \end{array}$$ (2)

where $p_{j}^{\left(v\right)}\;(t)$, $p_{m}^{\left(v\right)}\;(t)$, $g_{j}^{\left(v\right)}\;(t)$, and $p_{i}^{\left(h\right)}\;(t)$ represent the expression levels of EBV-protein *j*, EBV-protein *m*, EBV-gene *j*, and human-protein *i* at time *t*, respectively; $\alpha_{jm}^{\left(v\right)}$ and $\gamma_{ji}^{\left(v\right)}$ show the interactive abilities between EBV-protein *m* and EBV-protein *j*, and between human-protein *i* and EBV-protein *j*, respectively; $‑\sigma_{j}^{\left(v\right)}$, $\lambda_{j}^{\left(v\right)}$, and $\beta_{j}^{\left(v\right)}$ correspond to the degradation rate, the translation effect, and the basal level of EBV-protein *j*, respectively; *M*_*j*_ and *I*_*j*_ represent the numbers of EBV proteins and human proteins interacting with EBV-protein *j* in the candidate GIGEN, respectively; and $\varepsilon_{j}^{\left(v\right)}(t)$ is the stochastic noise of EBV-protein *j* owing to model uncertainty or other uncertain factors at time *t*. Note that the biological interaction mechanism of EBV proteins in (2) involves the intraspecies EBV PPIs represented by $\sum_{m=1}^{M_{i}}\alpha_{jm}^{\left(v\right)}p_{m}^{\left(v\right)}(t)p_{j}^{\left(v\right)}(t)$, and the interspecies PPIs represented by $\sum_{i=1}^{I_{i}}\gamma_{ji}^{\left(v\right)}p_{i}^{\left(h\right)}(t)p_{j}^{\left(v\right)}(t)$.

The GRN of human-gene *k* in the candidate GRN can be described by the following stochastic dynamic equation:

$$\begin{array}{l} g_{k}^{\left(h\right)}(t+1)=g_{k}^{\left(h\right)}(t)+\sum_{i=1}^{I_{k}}a_{ki}^{\left(h\right)}p_{i}^{\left(h\right)}(t)+\sum_{i'=1}^{I_{k}^{'}}\sum_{i''=1}^{I_{k}^{''}}\zeta_{k(I_{k}^{''}(i'-1)+i'')}^{\left(h\right)}p_{i'}^{\left(h\right)}(t)p_{i''}^{\left(h\right)}(t)\\ \;-\sum_{r=1}^{R_{k}}b_{kr}^{\left(h\right)}w_{r}^{\left(h\right)}(t)g_{k}^{\left(h\right)}(t)+\sum_{l=1}^{L_{k}}c_{kl}^{\left(h\right)}o_{l}^{\left(h\right)}(t)+\sum_{j=1}^{J_{k}}d_{kj}^{\left(h\right)}p_{j}^{\left(v\right)}(t)\\ \;-\sum_{q=1}^{Q_{k}}e_{kq}^{\left(h\right)}w_{q}^{\left(v\right)}(t)g_{k}^{\left(h\right)}(t)-\mu_{k}^{\left(h\right)}g_{k}^{\left(h\right)}(t)+\delta_{k}^{\left(h\right)}+\omega_{k}^{\left(h\right)}(t),\\ \;\mathrm{for}\;k=1,2,\ldots ,K,\;-b_{kr}^{\left(h\right)}\leq 0,\;-e_{kq}^{\left(h\right)}\leq 0,\;\mathrm{and}\;-\mu_{k}^{\left(h\right)}\leq 0 \end{array}$$ (3)

where $g_{k}^{\left(h\right)}(t)$, $p_{i}^{\left(h\right)}(t)$, $p_{i'}^{\left(h\right)}(t)p_{i''}^{\left(h\right)}(t)$, $w_{r}^{\left(h\right)}(t)$, $o_{l}^{\left(h\right)}(t)$, $p_{j}^{\left(v\right)}(t)$, and $w_{q}^{\left(v\right)}(t)$ indicate the expression levels of human-gene *k*, human-TF *i*, human-TF complex *i*'::*i*'', human-miRNA *r*, human-lncRNA *l*, EBV-TF *j*, and EBV-miRNA *q* at time *t*, respectively; human-TF complex *i*'::*i*'' is composed of human-TF *i*' and human-TF *i*''; $a_{ki}^{\left(h\right)}$, $\zeta_{k(I_{k}^{''}(i'-1)+i'')}^{\left(h\right)}$, $‑b_{kr}^{\left(h\right)}$, $c_{kl}^{\left(h\right)}$, $d_{kj}^{\left(h\right)}$, and $‑e_{kq}^{\left(h\right)}$ represent the regulatory abilities of human-TF *i* regulation, human-TF complex *i*'::*i*'' regulation, human-miRNA *r* repression, human-lncRNA *l* regulation, EBV-TF *j* regulation, and EBV-miRNA *q* repression on human-gene *k*, respectively; and $‑\mu_{k}^{\left(h\right)}$ and $\delta_{k}^{\left(h\right)}$ denote the degradation rate and the basal level of human-gene *k*, respectively. Remarkably, regarding the regulation ability $\zeta_{k(I_{k}^{''}(i'-1)+i'')}^{\left(h\right)}$ of the human TF complex on the human gene *k*, the index $I_{k}^{‘’}(i'-1)+i''$ assures the appropriate coordinate of the regulation ability $\zeta_{k(I_{k}^{''}(i'-1)+i'')}^{\left(h\right)}$ of the human TF complex $p_{i’}^{\left(h\right)}(t)p_{i’’}^{\left(h\right)}(t)$ in the human GRN of the system matrix of the human gene *k*, i.e., the regulation abilities of human TF complexes on the human gene *k* can be arranged as a one-row matrix as follows: $\zeta_{k1}^{\left(h\right)},\;\zeta_{k2}^{\left(h\right)},\;\ldots ,\;\zeta_{k(I_{k}^{''})}^{\left(h\right)},\;\zeta_{k(I_{k}^{''}+1)}^{\left(h\right)},\;\zeta_{k(I_{k}^{''}+2)}^{\left(h\right)},\;\ldots ,\;\zeta_{k(2I_{k}^{''})}^{\left(h\right)},\;\zeta_{k(2I_{k}^{''}+1)}^{\left(h\right)},\;\zeta_{k(2I_{k}^{''}+2)}^{\left(h\right)},\;\ldots ,\;\zeta_{k(3I_{k}^{''})}^{\left(h\right)},\;\ldots ,\;\zeta_{k(I_{k}^{''}(i'-1)+i'')}^{\left(h\right)},\;\ldots ,\;\zeta_{k(I_{k}^{''}I_{k}^{'})}^{\left(h\right)}$ The basal level $\delta_{k}^{\left(h\right)}$ denotes regulations from other unknown regulators. *I*_*k*_, $I_{k}^{’}$, $I_{k}^{’’}$, *R*_*k*_, *L*_*k*_, *J*_*k*_, and *Q*_*k*_ represent the numbers of human TFs, human TF complex subunit *i*', human TF complex subunit *i*'', human miRNAs, human lncRNAs, EBV TFs, and EBV miRNAs regulating human-gene *k* in the candidate GIGEN, respectively; and $\omega_{k}^{\left(h\right)}(t)$ is the stochastic noise of human-gene *k* owing to model uncertainty or other uncertain factors at time *t*—for example, methylation and histone modification. Note that the biological regulatory mechanism of human genes in (3) involves human-TF transcription regulations represented by $\sum_{i=1}^{I}a_{ki}^{\left(h\right)}\;p_{i}^{\left(h\right)}\;(t)$, human-TF complex transcription regulations represented by $\sum_{i’=1}^{I’}\sum_{i’’=1}^{I’’}$ $\zeta_{k(I_{k}^{''}(i'-1)+i'')}^{\left(h\right)}$ $p_{i’}^{\left(h\right)}\;(t)p_{i’’}^{\left(h\right)}\;(t)$, human-miRNA repressions represented by $‑\sum_{r=1}^{R}b_{kr}^{\left(h\right)}w_{r}^{\left(h\right)}(t)g_{k}^{\left(h\right)}(t)$, human-lncRNA regulations represented by $\sum_{l=1}^{L}c_{kl}^{\left(h\right)}o_{l}^{\left(h\right)}\;(t)$, EBV-TF transcription regulations represented by $\sum_{j=1}^{J}d_{kl}^{\left(h\right)}\;p_{j}^{\left(v\right)}\;(t)$, and EBV-miRNA repressions represented by $‑\sum_{q=1}^{Q}e_{kq}^{\left(h\right)}\;w_{q}^{\left(v\right)}\;(t)g_{k}^{\left(h\right)}\;(t)$. It seems reasonable to suppose that DNA methylation may have a robust connection with gene expression changes and be associated with the transcriptional activity of human genes. DNA methylation influences the dynamics and stability of RNA polymerase II elongation, so that intragenic DNA methylation coordinates differential gene expression via alternative promoters or splicing. Thus, we supposed that the differential changes of basal level $\delta_{k}^{\left(h\right)}$ of human-gene *k* between the first and second infection stage in Eq (3) were mainly due to DNA methylation during the EBV lytic infection.

The GRN of EBV-gene *s* in the candidate GRN can be described by the following stochastic dynamic equation:

$$\begin{array}{l} g_{s}^{\left(v\right)}(t+1)=g_{s}^{\left(v\right)}(t)+\sum_{i=1}^{I_{s}}a_{si}^{\left(v\right)}p_{i}^{\left(h\right)}(t)+\sum_{i'=1}^{I_{s}^{'}}\sum_{i''=1}^{I_{s}^{''}}\zeta_{s(I_{s}^{''}(i'-1)+i'')}^{\left(v\right)}p_{i'}^{\left(h\right)}(t)p_{i''}^{\left(h\right)}(t)\\ \;-\sum_{r=1}^{R_{s}}b_{sr}^{\left(v\right)}w_{r}^{\left(h\right)}(t)g_{s}^{\left(v\right)}(t)+\sum_{l=1}^{L_{s}}c_{sl}^{\left(v\right)}o_{l}^{\left(h\right)}(t)+\sum_{j=1}^{J_{s}}d_{sj}^{\left(v\right)}p_{j}^{\left(v\right)}(t)\\ \;-\sum_{q=1}^{Q_{s}}e_{sq}^{\left(v\right)}w_{q}^{\left(v\right)}(t)g_{s}^{\left(v\right)}(t)-\mu_{s}^{\left(v\right)}g_{s}^{\left(v\right)}(t)+\delta_{s}^{\left(v\right)}+\omega_{s}^{\left(v\right)}(t),\\ \;\mathrm{for}\;s=1,2,\ldots ,S,\;-b_{sr}^{\left(v\right)}\leq 0,\;-e_{sq}^{\left(v\right)}\leq 0,\;\mathrm{and}\;-\mu_{s}^{\left(v\right)}\leq 0 \end{array}$$ (4)

where $g_{s}^{\left(v\right)}(t)$ signifies the expression level of EBV-gene *s* at time *t*; $a_{si}^{\left(v\right)}$, $\zeta_{s(I_{s}^{''}(i'-1)+i'')}^{\left(v\right)}$, $‑b_{sr}^{\left(v\right)}$, $c_{sl}^{\left(v\right)}$, $d_{sj}^{\left(v\right)}$, and $‑e_{sq}^{\left(v\right)}$ show the regulatory abilities of human-TF *i* regulation, human-TF complex *i*'::*i*'' regulation, human-miRNA *r* repression, human-lncRNA *l* regulation, EBV-TF *j* regulation, and EBV-miRNA *q* repression on EBV-gene *s*, respectively; $‑\mu_{s}^{\left(v\right)}$ and $\delta_{s}^{\left(v\right)}$ correspond to the degradation rate and the basal level of EBV-gene *s*, respectively; and $\omega_{s}^{\left(v\right)}(t)$ is the stochastic noise of EBV-gene *s* owing to model uncertainty or other uncertain factors at time *t*. Note that the biological regulatory mechanism of EBV genes in (4) involves human-TF transcription regulations represented by $‑\sum_{i=1}^{I_{s}}a_{si}^{\left(v\right)}p_{i}^{\left(h\right)}(t)$, human-TF complex transcription regulations represented by $\sum_{i’=1}^{I_{s}’}\sum_{i’’-1}^{I_{s}’’}$ $\zeta_{s(I_{s}^{''}(i'-1)+i'')}^{\left(v\right)}$ $p_{i’}^{\left(h\right)}(t)p_{i’’}^{\left(h\right)}(t)$, human-miRNA repressions represented by $‑\sum_{r=1}^{R_{s}}b_{sr}^{\left(v\right)}w_{r}^{\left(h\right)}(t)g_{s}^{\left(v\right)}(t)$, human-lncRNA regulations represented by $\sum_{l=1}^{L_{s}}c_{sl}^{\left(v\right)}o_{l}^{\left(h\right)}\;(t)$, EBV-TF transcription regulations represented by $\sum_{j=1}^{J_{s}}d_{sj}^{\left(v\right)}p_{j}^{\left(v\right)}(t)$, and EBV-miRNA repressions represented by $‑\sum_{q=1}^{Q_{s}}e_{sq}^{\left(v\right)}w_{q}^{\left(v\right)}(t)g_{s}^{\left(v\right)}(t)$.

The GRN of human-lncRNA *z* in the candidate GRN can be described by the following stochastic dynamic equation:

$$\begin{array}{l} g_{z}^{\left(L\right)}(t+1)=g_{z}^{\left(L\right)}(t)+\sum_{i=1}^{I_{z}}a_{zi}^{\left(L\right)}p_{i}^{\left(h\right)}(t)+\sum_{i'=1}^{I_{z}^{'}}\sum_{i''=1}^{I_{z}^{''}}\zeta_{z(I_{z}^{''}(i'-1)+i'')}^{\left(L\right)}p_{i'}^{\left(h\right)}(t)p_{i''}^{\left(h\right)}(t)\\ \;-\sum_{r=1}^{R_{z}}b_{zr}^{\left(L\right)}w_{r}^{\left(h\right)}(t)g_{z}^{\left(L\right)}(t)+\sum_{l=1}^{L_{z}}c_{zl}^{\left(L\right)}o_{l}^{\left(h\right)}(t)+\sum_{j=1}^{J_{z}}d_{zj}^{\left(L\right)}p_{j}^{\left(v\right)}(t)\\ \;-\sum_{q=1}^{Q_{z}}e_{zq}^{\left(L\right)}w_{q}^{\left(v\right)}(t)g_{z}^{\left(L\right)}(t)-\mu_{z}^{\left(L\right)}g_{z}^{\left(L\right)}(t)+\delta_{z}^{\left(L\right)}+\omega_{z}^{\left(L\right)}(t),\\ \;\mathrm{for}\;z=1,2,\ldots ,Z,\;-b_{zr}^{\left(L\right)}\leq 0,\;-e_{zq}^{\left(L\right)}\leq 0,\;\mathrm{and}\;-\mu_{z}^{\left(L\right)}\leq 0 \end{array}$$ (5)

where $g_{z}^{\left(L\right)}(t)$ stands for the expression level of human-lncRNA *z* at time *t*; $a_{zi}^{\left(L\right)}$, $\zeta_{z(I_{z}^{''}(i'-1)+i'')}^{\left(L\right)}$, $‑b_{zr}^{\left(L\right)}$, $c_{zl}^{\left(L\right)}$, $d_{zj}^{\left(L\right)}$, and $‑e_{zq}^{\left(L\right)}$ indicate the regulatory abilities of human-TF *i* regulation, human-TF complex *i*'::*i*'' regulation, human-miRNA *r* repression, human-lncRNA *l* regulation, EBV-TF *j* regulation, and EBV-miRNA *q* repression on human-lncRNA *z*, respectively; $‑\mu_{z}^{\left(L\right)}$ and $\delta_{z}^{\left(L\right)}$ denote the degradation rate and the basal level of human-lncRNA *z*, respectively; and $\omega_{z}^{\left(L\right)}(t)$ is the stochastic noise of human-lncRNA *z* owing to model uncertainty or other uncertain factors at time *t*. Note that the biological regulatory mechanism of human lncRNAs in (5) involves human-TF transcription regulations represented by $\sum_{i=1}^{I_{z}}a_{zi}^{\left(L\right)}\;p_{i}^{\left(h\right)}\;(t)$, human-TF complex transcription regulations represented by ∑i’=1Iz’∑i’’=1Iz'' $\zeta_{z(I_{z}^{''}(i'-1)+i'')}^{\left(L\right)}$ $p_{i’}^{\left(h\right)}\;(t)p_{i’’}^{\left(h\right)}\;(t)$, human-miRNA repressions represented by $-\sum_{r=1}^{R_{z}}b_{zr}^{\left(L\right)}w_{r}^{\left(h\right)}\;(t)g_{z}^{\left(L\right)}\;(t)$, human-lncRNA regulations represented by $\sum_{l=1}^{L_{z}}c_{zl}^{\left(L\right)}\;o_{l}^{\left(h\right)}\;(t)$, EBV-TF transcription regulations represented by $\sum_{j=1}^{J_{z}}d_{zj}^{\left(L\right)}\;p_{j}^{\left(v\right)}\;(t)$, and EBV-miRNA repressions represented by $-\sum_{q=1}^{Q_{z}}e_{zq}^{\left(L\right)}w_{q}^{\left(v\right)}(t)g_{z}^{\left(L\right)}(t)$.

The GRN of human-miRNA *f* in the candidate GRN can be described by the following stochastic dynamic equation:

$$\begin{array}{l} x_{f}^{\left(h\right)}(t+1)=x_{f}^{\left(h\right)}(t)+\sum_{i=1}^{I_{f}}{\bar{a}}_{fi}^{\left(h\right)}p_{i}^{\left(h\right)}(t)+\sum_{i'=1}^{I_{f}^{'}}\sum_{i''=1}^{I_{f}^{''}}{\bar{\zeta }}_{f(I_{f}^{''}(i'-1)+i'')}^{\left(h\right)}p_{i'}^{\left(h\right)}(t)p_{i''}^{\left(h\right)}(t)\\ \;-\sum_{r=1}^{R_{f}}{\bar{b}}_{fr}^{\left(h\right)}w_{r}^{\left(h\right)}(t)x_{f}^{\left(h\right)}(t)+\sum_{j=1}^{J_{f}}{\bar{d}}_{fj}^{\left(h\right)}p_{j}^{\left(v\right)}(t)\\ \;-\sum_{q=1}^{Q_{f}}{\bar{e}}_{fq}^{\left(h\right)}w_{q}^{\left(v\right)}(t)x_{f}^{\left(h\right)}(t)-\rho_{f}^{\left(h\right)}x_{f}^{\left(h\right)}(t)+\eta_{f}^{\left(h\right)}+\psi_{f}^{\left(h\right)}(t),\\ \;\mathrm{for}\;f=1,2,\ldots ,F,\;-{\bar{b}}_{fr}^{\left(h\right)}\leq 0,\;-{\bar{e}}_{fq}^{\left(h\right)}\leq 0,\;\mathrm{and}\;-\rho_{f}^{\left(h\right)}\leq 0 \end{array}$$ (6)

where $x_{f}^{\left(h\right)}\;(t)$ represents the expression level of human-miRNA *f* at time *t*; ${\bar{a}}_{fi}^{\left(h\right)}$, ${\bar{\zeta }}_{f(I_{f}^{''}(i'-1)+i'')}^{\left(h\right)}$, $-{\bar{b}}_{fr}^{\left(h\right)}$, ${\bar{d}}_{fj}^{\left(h\right)}$, and $-{\bar{e}}_{fq}^{\left(h\right)}$ mean the regulatory abilities of human-TF *i* regulation, human-TF complex *i*'::*i*'' regulation, human-miRNA *r* repression, EBV-TF *j* regulation, and EBV-miRNA *q* repression on human-miRNA *f*, respectively; $‑\rho_{f}^{\left(h\right)}$ and $\eta_{f}^{\left(h\right)}$ signify the degradation rate and the basal level of human-miRNA *f*, respectively; and $\psi_{f}^{\left(h\right)}\;(t)$ is the stochastic noise of human-miRNA *f* owing to model uncertainty or other uncertain factors at time *t*. Note that the biological regulatory mechanism of human miRNAs in (6) involves human-TF transcription regulations represented by $\sum_{i=1}^{I_{f}}{\bar{a}}_{fi}^{\left(h\right)}p_{i}^{\left(h\right)}(t)$, human-TF complex transcription regulations represented by $\sum_{i'=1}^{I_{f}^{'}}\sum_{i''=1}^{I_{f}^{''}}{\bar{\zeta }}_{f(I_{f}^{''}(i'-1)+i'')}^{\left(h\right)}p_{i'}^{\left(h\right)}(t)p_{i''}^{\left(h\right)}(t)$, human-miRNA repressions represented by $-\sum_{r=1}^{R_{f}}{\bar{b}}_{fr}^{\left(h\right)}w_{r}^{\left(h\right)}(t)x_{f}^{\left(h\right)}(t)$, EBV-TF transcription regulations represented by $\sum_{j=1}^{J_{f}}{\bar{d}}_{fj}^{\left(h\right)}p_{j}^{\left(v\right)}(t)$, and EBV-miRNA repressions represented by $-\sum_{q=1}^{Q_{f}}{\bar{e}}_{fq}^{\left(h\right)}w_{q}^{\left(v\right)}(t)x_{f}^{\left(h\right)}(t)$.

The GRN of EBV-miRNA *u* in the candidate GRN can be described by the following stochastic dynamic equation:

$$\begin{array}{l} x_{u}^{\left(v\right)}(t+1)=x_{u}^{\left(v\right)}(t)+\sum_{i=1}^{I_{u}}{\bar{a}}_{ui}^{\left(v\right)}p_{i}^{\left(h\right)}(t)+\sum_{i'=1}^{I_{u}^{'}}\sum_{i''=1}^{I_{u}^{''}}{\bar{\zeta }}_{u(I_{u}^{''}(i'-1)+i'')}^{\left(v\right)}p_{i'}^{\left(h\right)}(t)p_{i''}^{\left(h\right)}(t)\\ \;-\sum_{r=1}^{R_{u}}{\bar{b}}_{ur}^{\left(v\right)}w_{r}^{\left(h\right)}(t)x_{u}^{\left(v\right)}(t)+\sum_{j=1}^{J_{u}}{\bar{d}}_{uj}^{\left(v\right)}p_{j}^{\left(v\right)}(t)\\ \;-\sum_{q=1}^{Q_{u}}{\bar{e}}_{uq}^{\left(v\right)}w_{q}^{\left(v\right)}(t)x_{u}^{\left(v\right)}(t)-\rho_{u}^{\left(v\right)}x_{u}^{\left(v\right)}(t)+\eta_{u}^{\left(v\right)}+\psi_{u}^{\left(v\right)}(t),\\ \;\mathrm{for}\;u=1,2,\ldots ,U,\;-{\bar{b}}_{ur}^{\left(v\right)}\leq 0,\;-{\bar{e}}_{uq}^{\left(v\right)}\leq 0,\;\mathrm{and}-\rho_{u}^{\left(v\right)}\leq 0 \end{array}$$ (7)

where $x_{u}^{\left(v\right)}(t)$ shows the expression level of EBV-miRNA *u* at time *t*; ${\bar{a}}_{ui}^{\left(v\right)}$, ${\bar{\zeta }}_{u(I_{u}^{''}(i'-1)+i'')}^{\left(v\right)}$, $-{\bar{b}}_{ur}^{\left(v\right)}$, ${\bar{d}}_{uj}^{\left(v\right)}$, and $-{\bar{e}}_{uq}^{\left(v\right)}$ correspond to the regulatory abilities of human-TF *i* regulation, human-TF complex *i*'::*i*'' regulation, human-miRNA *r* repression, EBV-TF *j* regulation, and EBV-miRNA *q* repression on EBV-miRNA *u*, respectively; $‑\rho_{u}^{\left(v\right)}$ and $\eta_{u}^{\left(v\right)}$ stand for the degradation rate and the basal level of EBV-miRNA *u*, respectively; and $\psi_{u}^{\left(v\right)}(t)$ is the stochastic noise of EBV-miRNA *u* owing to model uncertainty or other uncertain factors at time *t*. Note that the biological regulatory mechanism of EBV miRNAs in (7) involves human-TF transcription regulations represented by $\sum_{i=1}^{I_{u}}{\bar{a}}_{ui}^{\left(v\right)}p_{i}^{\left(h\right)}(t)$, human-TF complex transcription regulations represented by $\sum_{i'=1}^{I_{u}^{'}}\sum_{i''=1}^{I_{u}^{''}}{\bar{\zeta }}_{u(I_{u}^{''}(i'-1)+i'')}^{\left(v\right)}p_{i'}^{\left(h\right)}(t)p_{i''}^{\left(h\right)}(t)$, human-miRNA repressions represented by $-\sum_{r=1}^{R_{u}}{\bar{b}}_{ur}^{\left(v\right)}w_{r}^{\left(h\right)}(t)x_{u}^{\left(v\right)}(t)$, EBV-TF transcription regulations represented by $\sum_{j=1}^{J_{u}}{\bar{d}}_{uj}^{\left(v\right)}p_{j}^{\left(v\right)}(t)$, and EBV-miRNA repressions represented by $-\sum_{q=1}^{Q_{u}}{\bar{e}}_{uq}^{\left(v\right)}w_{q}^{\left(v\right)}(t)x_{u}^{\left(v\right)}(t)$.

### System identification approach for the dynamic models of GIGEN

After establishing the stochastic dynamic model Eqs (1)–(7) for the characterization of the molecular mechanism in the GIGEN, we identified the interactive parameters of PPIN in (1) and (2), and the regulatory parameters of GRN in (3)–(7) using the system identification approach to solve the parameter estimation problems for the purpose of pruning the false positives under infection conditions. Thus, we rewrote human PPIN Eq (1) as the following linear regression:

$$\begin{array}{r} p_{i}^{\left(h\right)}(t+1)=[\begin{array}{cccc} p_{1}^{\left(h\right)}(t)p_{i}^{\left(h\right)}(t) & \cdots & p_{N_{i}}^{\left(h\right)}(t)p_{i}^{\left(h\right)}(t) & p_{1}^{\left(v\right)}(t)p_{i}^{\left(h\right)}(t) \end{array}\\ \begin{array}{ccccc} \cdots & p_{J_{i}}^{\left(v\right)}(t)p_{i}^{\left(h\right)}(t) & g_{i}^{\left(h\right)}(t) & p_{i}^{\left(h\right)}(t) & 1 \end{array}]\left[\begin{array}{c} \alpha_{i1}^{\left(h\right)}\\ \vdots \\ \alpha_{iN_{i}}^{\left(h\right)}\\ \gamma_{i1}^{\left(h\right)}\\ \vdots \\ \gamma_{iJ_{i}}^{\left(h\right)}\\ \lambda_{i}^{\left(h\right)}\\ 1-\sigma_{i}^{\left(h\right)}\\ \beta_{i}^{\left(h\right)} \end{array}\right]\\ +\varepsilon_{i}^{\left(h\right)}(t),\mathrm{for}\;i=1,2,\ldots ,I,\;-\sigma_{i}^{\left(h\right)}\leq 0,\;\mathrm{and}\lambda_{i}^{\left(h\right)}\geq 0 \end{array}$$ (8)

which can be simplified to:

$$p_{i}^{\left(h\right)}(t+1)=\phi_{i}^{HP}(t)\theta_{i}^{HP}+\varepsilon_{i}^{\left(h\right)}(t),\;\mathrm{for}\;i=1,2,\ldots ,I,\;-\sigma_{i}^{\left(h\right)}\leq 0,\;\mathrm{and}\lambda_{i}^{\left(h\right)}\geq 0$$ (9)

where $\phi_{i}^{HP}\;(t)$ indicates the regression vector obtained from the corresponding expression data, and $\theta_{i}^{HP}$ denotes the unknown interaction parameter vector of human-protein *i* in the human PPIN to be estimated. Eq (9) could be augmented for *Y*_*i*_ data points of human-protein *i* as follows:

$$\left[\begin{array}{c} p_{i}^{\left(h\right)}(t_{2})\\ p_{i}^{\left(h\right)}(t_{3})\\ \vdots \\ p_{i}^{\left(h\right)}(t_{Y_{i}}+1) \end{array}\right]=\left[\begin{array}{c} \phi_{i}^{HP}(t_{1})\\ \phi_{i}^{HP}(t_{2})\\ \vdots \\ \phi_{i}^{HP}(t_{Y_{i}}) \end{array}\right]\theta_{i}^{HP}+\left[\begin{array}{c} \varepsilon_{i}^{\left(h\right)}(t_{1})\\ \varepsilon_{i}^{\left(h\right)}(t_{2})\\ \vdots \\ \varepsilon_{i}^{\left(h\right)}(t_{Y_{i}}) \end{array}\right],\;\mathrm{for}\;i=1,2,\ldots ,I,\;-\sigma_{i}^{\left(h\right)}\leq 0,\;\mathrm{and}\;\lambda_{i}^{\left(h\right)}\geq 0$$ (10)

where *Y*_*i*_ is the number of data points of protein expression. Thus, we defined the notations $P_{i}^{\left(h\right)}$, $\Phi_{i}^{HP}$, and $\Xi_{i}^{HP}$ to represent Eq (10) as follows:

$$P_{i}^{\left(h\right)}=\Phi_{i}^{HP}\theta_{i}^{HP}+\Xi_{i}^{HP},\;\mathrm{for}\;i=1,2,\ldots ,I,\;-\sigma_{i}^{\left(h\right)}\leq 0,\;\mathrm{and}\;\lambda_{i}^{\left(h\right)}\geq 0$$ (11)

where $P_{i}^{\left(h\right)}=\left[\begin{array}{c} p_{i}^{\left(h\right)}(t_{2})\\ p_{i}^{\left(h\right)}(t_{3})\\ \vdots \\ p_{i}^{\left(h\right)}(t_{Y_{i}}+1) \end{array}\right],\;\Phi_{i}^{HP}=\left[\begin{array}{c} \phi_{i}^{HP}(t_{1})\\ \phi_{i}^{HP}(t_{2})\\ \vdots \\ \phi_{i}^{HP}(t_{Y_{i}}) \end{array}\right],\;\Xi_{i}^{HP}=\left[\begin{array}{c} \varepsilon_{i}^{\left(h\right)}(t_{1})\\ \varepsilon_{i}^{\left(h\right)}(t_{2})\\ \vdots \\ \varepsilon_{i}^{\left(h\right)}(t_{Y_{i}}) \end{array}\right]$.

Next, we formulated the parameter estimation of $\theta_{i}^{HP}$ as the following constrained least square equation:

$$\underset{\theta_{i}^{HP}}{\min}\frac{1}{2}{\left\|\Phi_{i}^{HP}\theta_{i}^{HP}-P_{i}^{\left(h\right)}\right\|}_{2}^{2}\;\mathrm{subject}\;\mathrm{to}\;A\theta_{i}^{HP}\leq b$$ (12)

where $A=\overset{N_{i}+J_{i}}{\overset{︷}{[\begin{array}{ccc} 0 & \cdots & 0\\ 0 & \cdots & 0 \end{array}}}\begin{array}{ccc} -1 & 0 & 0\\ 0 & 1 & 0 \end{array}],\;b=\left[\begin{array}{c} 0\\ 1 \end{array}\right]$.

By solving the parameter estimation in (12) with the help of the *lsqlin* function in the MATLAB optimization toolbox, we acquired the interaction parameters in human PPIN Eq (1), and concurrently ensured that the human-protein translation rate $\lambda_{i}^{\left(h\right)}$ was a non-negative value and the human-protein degradation rate $‑\sigma_{i}^{\left(h\right)}$ was a non-positive value; that is to say $\lambda_{i}^{\left(h\right)}\geq 0$ and $‑\sigma_{i}^{\left(h\right)}\leq 0$. Similarly, system identification approach for the other Eqs (2)–(7) in the GIGEN is shown in S1 Text.

To obtain accurate results in the system identification approach, it is necessary to interpolate some extra data points (5 times the number of parameters in the corresponding parameter vectors: $\theta_{i}^{HP}$ in human PPIN, $\theta_{j}^{VP}$ in EBV PPIN, $\theta_{k}^{HG}$ in human-gene GRN, $\theta_{s}^{VG}$ in EBV-gene GRN, $\theta_{z}^{HL}$ in human-lncRNA GRN, $\tau_{f}^{HM}$ in human-miRNA GRN, and $\tau_{u}^{VM}$ in the EBV-miRNA GRN to be estimated) via the cubic spline method mentioned above, which solves the parameter estimation problem by preventing overfitting owing to insufficient data points. Therefore, the solutions to constrained least square parameter estimation Eq (12), (S5), (S10), (S15), (S20), (S25), and (S30) can be obtained with NGS expression data using the *lsqlin* function in the MATLAB optimization toolbox for the optimal estimation of the parameters in these estimation equations. There remains an unsettled question: the genome-wide mRNA microarray expression measurement cannot describe the protein behavior in human B cells and EBV, but the corresponding mRNA abundance can explain over 73% of the variance in protein abundance [126]; that is to say, protein behavior can be described by the corresponding gene expression. As a result, the NGS gene expression data can replace the protein expression data, thereby contributing to the solution of constrained least square parameter estimation Eq (12), (S5), (S10), (S15), (S20), (S25), and (S30).

### System order detection scheme for the dynamic models of GIGEN

The candidate GIGEN constructed by database mining with computational and experimental predictions contained many false positives for the interactive and regulatory parameters. Therefore, we applied the system order detection scheme to the human PPIN model in (11), the EBV PPIN model in (S4), the human-gene GRN model in (S9), the EBV-gene GRN model in (S14), the human-lncRNA GRN model in (S19), the human-miRNA GRN model in (S24), and the EBV-miRNA GRN model in (S29) to prune the false positives in the candidate GIGEN using the NGS human B cell and EBV data at the first and second infection stages. According to the Akaike information criterion (AIC), the insignificant parameters in the models of GIGEN were deleted so that we ultimately acquired the real GIGENs at the first and second infection stages during the EBV lytic phase. In the human PPIN model (11), the AIC of human-protein *i* can be defined as follows [121]:

$$\begin{array}{l} AIC_{i}^{HP}\;\left(N_{i},J_{i}\right)=\log\left(\frac{1}{Y_{i}}{\left(P_{i}^{\left(h\right)}-\Phi_{i}^{HP}{\hat{\theta }}_{i}^{HP}\right)}^{T}\left(P_{i}^{\left(h\right)}-\Phi_{i}^{HP}{\hat{\theta }}_{i}^{HP}\right)\right)\\ \;+\frac{2\left(N_{i}+J_{i}\right)}{Y_{i}} \end{array}$$ (13)

()where ${\hat{\theta }}_{i}^{HP}$ indicates the estimated parameters of human-protein *i* obtained from the solution of parameter estimation Eq (12), and the estimated residual error is ${\hat{\kappa }}_{HP,i}^{2}=\frac{1}{Y_{i}}{\left(P_{i}^{\left(h\right)}-\Phi_{i}^{HP}{\hat{\theta }}_{i}^{HP}\right)}^{T}\left(P_{i}^{\left(h\right)}-\Phi_{i}^{HP}{\hat{\theta }}_{i}^{HP}\right)$.

Because the parameter estimation in (12) has less overfit for large enough data points, we applied cubic spline interpolation to the time-course data to increase the number of data points *Y*_*i*_. In (13), the first quantity measures the model fit, and the second quantity penalizes for overfit. Philosophically, AIC in (13) is an estimate of the expected relative distance between the fitted model and the unknown true mechanism that actually generated the observed data [127]. Therefore, the true number (or order) of regulations and interactions is obtained by minimizing *AIC* in (13). Those insignificant regulations and interactions are considered as false positives to be deleted from the candidate network. According to system identification theory, the AIC is a tradeoff between estimated residual error and parameter-associated error, and will be minimal at the real system order (i.e., the number of parameters). The minimum $AIC_{i}^{HP}$ in (13) can be solved for number $N_{i}^{*}+J_{i}^{*}$ of the real PPIs of protein *i* in the human PPIN. The insignificant interactions of $N_{i}^{*}$ and $J_{i}^{*}$ should be deleted as false positives from PPIs of protein *i*. We then obtained the real human PPIN by applying a similar procedure one protein at a time. Similarly, system order detection scheme for the other equations (S4), (S9), (S14), (S19), (S24), and (S29) in the GIGEN is shown in S2 Text. Furthermore, in order to evaluate statistical significance of real human PPIN, student’s *t*-test was used to calculate the statistical significance (*p* value) of the parameters in (1) in the real human PPI network under the null hypothesis *H*_0_: $\alpha_{in}^{\left(h\right)}=0$ and $\gamma_{ij}^{\left(h\right)}=0$ in (1) [128].

Therefore, we were able to identify the real GIGENs at the first and second infection stages (Figs 3 and 4, respectively) during EBV lytic infection; once we had applied the system identification approach and the system order detection scheme, we obtained the GIGENs by pruning the false positives of the candidate GIGEN using NGS human B cell and EBV data. Information concerning the number of nodes and edges of the candidate GIGEN from databases, and the number of nodes and edges of the real GIGENs at the first and second infection stages are presented in Table 1A and 1B, respectively. However, the real GIGENs shown in Figs 3 and 4 were too complicated to allow us to investigate the lytic replication, production, and cytolytic mechanisms in humans and EBV during lytic infection. Therefore, we extracted the HVCNs, which contain the principal network structures of the real networks, from the real GIGENs at both infection stages in the EBV lytic phase using the PNP method.

### Extracting the core network from the real GIGEN using the PNP method

It is essential to establish an integrated system network matrix *H* of a real GIGEN before applying the PNP method to extract the core GIGEN from the real GIGEN. Furthermore, the system network matrix *H* includes the whole estimated system parameters in the real GIGEN as follows:

$$H=\left[\begin{array}{cccccc} 0 & H_{vp,vp} & 0 & H_{vp,hp} & 0 & 0\\ 0 & H_{hp,vp} & 0 & H_{hp,hp} & 0 & 0\\ H_{vm,vm} & H_{vm,vp} & H_{vm,hm} & H_{vm,hp} & H_{vm,hc} & 0\\ H_{vg,vm} & H_{vg,vp} & H_{vg,hm} & H_{vg,hp} & H_{vg,hc} & H_{vg,hl}\\ H_{hm,vm} & H_{hm,vp} & H_{hm,hm} & H_{hm,hp} & H_{hm,hc} & 0\\ H_{hg,vm} & H_{hg,vp} & H_{hg,hm} & H_{hg,hp} & H_{hg,hc} & H_{hg,hl}\\ H_{hl,vm} & H_{hl,vp} & H_{hl,hm} & H_{hl,hp} & H_{hl,hc} & H_{hl,hl} \end{array}\right]\in {\mathbb{R}}^{\left(2J+2I+Q+R+L\right)\times \left(J+I+Q+R+L+I'I''\right)}$$

$$\begin{array}{l} \mathrm{where}\;H_{vp,vp}=\left[\begin{array}{ccc} {\hat{\alpha }}_{11}^{\left(v\right)} & \cdots & {\hat{\alpha }}_{1J}^{\left(v\right)}\\ \vdots & {\hat{\alpha }}_{jm}^{\left(v\right)} & \vdots \\ {\hat{\alpha }}_{J1}^{\left(v\right)} & \cdots & {\hat{\alpha }}_{JJ}^{\left(v\right)} \end{array}\right],H_{vp,hp}=\left[\begin{array}{ccc} {\hat{\gamma }}_{11}^{\left(v\right)} & \cdots & {\hat{\gamma }}_{1I}^{\left(v\right)}\\ \vdots & {\hat{\gamma }}_{ji}^{\left(v\right)} & \vdots \\ {\hat{\gamma }}_{J1}^{\left(v\right)} & \cdots & {\hat{\gamma }}_{JI}^{\left(v\right)} \end{array}\right],H_{hp,vp}=\left[\begin{array}{ccc} {\hat{\gamma }}_{11}^{\left(h\right)} & \cdots & {\hat{\gamma }}_{1J}^{\left(h\right)}\\ \vdots & {\hat{\gamma }}_{ij}^{\left(h\right)} & \vdots \\ {\hat{\gamma }}_{I1}^{\left(h\right)} & \cdots & {\hat{\gamma }}_{IJ}^{\left(h\right)} \end{array}\right],\\ H_{hp,hp}=\left[\begin{array}{ccc} {\hat{\alpha }}_{11}^{\left(h\right)} & \cdots & {\hat{\alpha }}_{1I}^{\left(h\right)}\\ \vdots & {\hat{\alpha }}_{in}^{\left(h\right)} & \vdots \\ {\hat{\alpha }}_{I1}^{\left(h\right)} & \cdots & {\hat{\alpha }}_{II}^{\left(h\right)} \end{array}\right],H_{vm,vm}=\left[\begin{array}{ccc} -{\hat{\bar{e}}}_{11}^{\left(v\right)} & \cdots & -{\hat{\bar{e}}}_{1Q}^{\left(v\right)}\\ \vdots & -{\hat{\bar{e}}}_{uq}^{\left(v\right)} & \vdots \\ -{\hat{\bar{e}}}_{Q1}^{\left(v\right)} & \cdots & -{\hat{\bar{e}}}_{QQ}^{\left(v\right)} \end{array}\right],H_{vm,vp}=\left[\begin{array}{ccc} {\hat{\bar{d}}}_{11}^{\left(v\right)} & \cdots & {\hat{\bar{d}}}_{1J}^{\left(v\right)}\\ \vdots & {\hat{\bar{d}}}_{uj}^{\left(v\right)} & \vdots \\ {\hat{\bar{d}}}_{Q1}^{\left(v\right)} & \cdots & {\hat{\bar{d}}}_{QJ}^{\left(v\right)} \end{array}\right],\\ H_{vm,hm}=\left[\begin{array}{ccc} -{\hat{\bar{b}}}_{11}^{\left(v\right)} & \cdots & -{\hat{\bar{b}}}_{1R}^{\left(v\right)}\\ \vdots & -{\hat{\bar{b}}}_{ur}^{\left(v\right)} & \vdots \\ -{\hat{\bar{b}}}_{Q1}^{\left(v\right)} & \cdots & -{\hat{\bar{b}}}_{QR}^{\left(v\right)} \end{array}\right],H_{vm,hp}=\left[\begin{array}{ccc} {\hat{\bar{a}}}_{11}^{\left(v\right)} & \cdots & {\hat{\bar{a}}}_{1I}^{\left(v\right)}\\ \vdots & {\hat{\bar{a}}}_{ui}^{\left(v\right)} & \vdots \\ {\hat{\bar{a}}}_{Q1}^{\left(v\right)} & \cdots & {\hat{\bar{a}}}_{QI}^{\left(v\right)} \end{array}\right],H_{vm,hc}=\left[\begin{array}{ccc} {\hat{\bar{\zeta }}}_{11}^{\left(v\right)} & \cdots & {\hat{\bar{\zeta }}}_{1I'I''}^{\left(v\right)}\\ \vdots & {\hat{\bar{\zeta }}}_{u(I''(i'-1)+i'')}^{\left(v\right)} & \vdots \\ {\hat{\bar{\zeta }}}_{Q1}^{\left(v\right)} & \cdots & {\hat{\bar{\zeta }}}_{QI'I''}^{\left(v\right)} \end{array}\right],\\ H_{vg,vm}=\left[\begin{array}{ccc} -{\hat{e}}_{11}^{\left(v\right)} & \cdots & -{\hat{e}}_{1Q}^{\left(v\right)}\\ \vdots & -{\hat{e}}_{sq}^{\left(v\right)} & \vdots \\ -{\hat{e}}_{J1}^{\left(v\right)} & \cdots & -{\hat{e}}_{JQ}^{\left(v\right)} \end{array}\right],H_{vg,vp}=\left[\begin{array}{ccc} {\hat{d}}_{11}^{\left(v\right)} & \cdots & {\hat{d}}_{1J}^{\left(v\right)}\\ \vdots & {\hat{d}}_{sj}^{\left(v\right)} & \vdots \\ {\hat{d}}_{J1}^{\left(v\right)} & \cdots & {\hat{d}}_{JJ}^{\left(v\right)} \end{array}\right],H_{vg,hm}=\left[\begin{array}{ccc} -{\hat{b}}_{11}^{\left(v\right)} & \cdots & -{\hat{b}}_{1R}^{\left(v\right)}\\ \vdots & -{\hat{b}}_{sr}^{\left(v\right)} & \vdots \\ -{\hat{b}}_{J1}^{\left(v\right)} & \cdots & -{\hat{b}}_{JR}^{\left(v\right)} \end{array}\right],\\ H_{vg,hp}=\left[\begin{array}{ccc} {\hat{a}}_{11}^{\left(v\right)} & \cdots & {\hat{a}}_{1I}^{\left(v\right)}\\ \vdots & {\hat{a}}_{si}^{\left(v\right)} & \vdots \\ {\hat{a}}_{J1}^{\left(v\right)} & \cdots & {\hat{a}}_{JI}^{\left(v\right)} \end{array}\right],H_{vg,hc}=\left[\begin{array}{ccc} {\hat{\zeta }}_{11}^{\left(v\right)} & \cdots & {\hat{\zeta }}_{1I'I''}^{\left(v\right)}\\ \vdots & {\hat{\zeta }}_{s(I''(i'-1)+i'')}^{\left(v\right)} & \vdots \\ {\hat{\zeta }}_{J1}^{\left(v\right)} & \cdots & {\hat{\zeta }}_{JI'I''}^{\left(v\right)} \end{array}\right],H_{vg,hl}=\left[\begin{array}{ccc} {\hat{c}}_{11}^{\left(v\right)} & \cdots & {\hat{c}}_{1L}^{\left(v\right)}\\ \vdots & {\hat{c}}_{sl}^{\left(v\right)} & \vdots \\ {\hat{c}}_{J1}^{\left(v\right)} & \cdots & {\hat{c}}_{JL}^{\left(v\right)} \end{array}\right],\\ H_{hm,vm}=\left[\begin{array}{ccc} -{\hat{\bar{e}}}_{11}^{\left(h\right)} & \cdots & -{\hat{\bar{e}}}_{1Q}^{\left(h\right)}\\ \vdots & -{\hat{\bar{e}}}_{fq}^{\left(h\right)} & \vdots \\ -{\hat{\bar{e}}}_{R1}^{\left(h\right)} & \cdots & -{\hat{\bar{e}}}_{RQ}^{\left(h\right)} \end{array}\right],H_{hm,vp}=\left[\begin{array}{ccc} {\hat{\bar{d}}}_{11}^{\left(h\right)} & \cdots & {\hat{\bar{d}}}_{1J}^{\left(h\right)}\\ \vdots & {\hat{\bar{d}}}_{fj}^{\left(h\right)} & \vdots \\ {\hat{\bar{d}}}_{R1}^{\left(h\right)} & \cdots & {\hat{\bar{d}}}_{RJ}^{\left(h\right)} \end{array}\right],H_{hm,hm}=\left[\begin{array}{ccc} -{\hat{\bar{b}}}_{11}^{\left(h\right)} & \cdots & -{\hat{\bar{b}}}_{1R}^{\left(h\right)}\\ \vdots & -{\hat{\bar{b}}}_{fr}^{\left(h\right)} & \vdots \\ -{\hat{\bar{b}}}_{R1}^{\left(h\right)} & \cdots & -{\hat{\bar{b}}}_{RR}^{\left(h\right)} \end{array}\right], \end{array}$$

$$\begin{array}{l} H_{hm,hp}=\left[\begin{array}{ccc} {\hat{\bar{a}}}_{11}^{\left(h\right)} & \cdots & {\hat{\bar{a}}}_{1I}^{\left(h\right)}\\ \vdots & {\hat{\bar{a}}}_{fi}^{\left(h\right)} & \vdots \\ {\hat{\bar{a}}}_{R1}^{\left(h\right)} & \cdots & {\hat{\bar{a}}}_{RI}^{\left(h\right)} \end{array}\right],H_{hm,hc}=\left[\begin{array}{ccc} {\hat{\bar{\zeta }}}_{11}^{\left(h\right)} & \cdots & {\hat{\bar{\zeta }}}_{1I'I''}^{\left(h\right)}\\ \vdots & {\hat{\bar{\zeta }}}_{f(I''(i'-1)+i'')}^{\left(h\right)} & \vdots \\ {\hat{\bar{\zeta }}}_{R1}^{\left(h\right)} & \cdots & {\hat{\bar{\zeta }}}_{RI'I''}^{\left(h\right)} \end{array}\right],H_{hg,vm}=\left[\begin{array}{ccc} -{\hat{e}}_{11}^{\left(h\right)} & \cdots & -{\hat{e}}_{1Q}^{\left(h\right)}\\ \vdots & -{\hat{e}}_{kq}^{\left(h\right)} & \vdots \\ -{\hat{e}}_{I1}^{\left(h\right)} & \cdots & -{\hat{e}}_{IQ}^{\left(h\right)} \end{array}\right],\\ H_{hg,vp}=\left[\begin{array}{ccc} {\hat{d}}_{11}^{\left(h\right)} & \cdots & {\hat{d}}_{1J}^{\left(h\right)}\\ \vdots & {\hat{d}}_{kj}^{\left(h\right)} & \vdots \\ {\hat{d}}_{I1}^{\left(h\right)} & \cdots & {\hat{d}}_{IJ}^{\left(h\right)} \end{array}\right],H_{hg,hm}=\left[\begin{array}{ccc} -{\hat{b}}_{11}^{\left(h\right)} & \cdots & -{\hat{b}}_{1R}^{\left(h\right)}\\ \vdots & -{\hat{b}}_{kr}^{\left(h\right)} & \vdots \\ -{\hat{b}}_{I1}^{\left(h\right)} & \cdots & -{\hat{b}}_{IR}^{\left(h\right)} \end{array}\right],H_{hg,hp}=\left[\begin{array}{ccc} {\hat{a}}_{11}^{\left(h\right)} & \cdots & {\hat{a}}_{1I}^{\left(h\right)}\\ \vdots & {\hat{a}}_{ki}^{\left(h\right)} & \vdots \\ {\hat{a}}_{I1}^{\left(h\right)} & \cdots & {\hat{a}}_{II}^{\left(h\right)} \end{array}\right],\\ H_{hg,hc}=\left[\begin{array}{ccc} {\hat{\zeta }}_{11}^{\left(h\right)} & \cdots & {\hat{\zeta }}_{1I'I''}^{\left(h\right)}\\ \vdots & {\hat{\zeta }}_{k(I''(i'-1)+i'')}^{\left(h\right)} & \vdots \\ {\hat{\zeta }}_{I1}^{\left(h\right)} & \cdots & {\hat{\zeta }}_{II'I''}^{\left(h\right)} \end{array}\right],H_{hg,hl}=\left[\begin{array}{ccc} {\hat{c}}_{11}^{\left(h\right)} & \cdots & {\hat{c}}_{1L}^{\left(h\right)}\\ \vdots & {\hat{c}}_{kl}^{\left(h\right)} & \vdots \\ {\hat{c}}_{I1}^{\left(h\right)} & \cdots & {\hat{c}}_{IL}^{\left(h\right)} \end{array}\right],H_{hl,vm}=\left[\begin{array}{ccc} -{\hat{e}}_{11}^{\left(L\right)} & \cdots & -{\hat{e}}_{1Q}^{\left(L\right)}\\ \vdots & -{\hat{e}}_{zq}^{\left(L\right)} & \vdots \\ -{\hat{e}}_{L1}^{\left(L\right)} & \cdots & -{\hat{e}}_{LQ}^{\left(L\right)} \end{array}\right],\\ H_{hl,vp}=\left[\begin{array}{ccc} {\hat{d}}_{11}^{\left(L\right)} & \cdots & {\hat{d}}_{1J}^{\left(L\right)}\\ \vdots & {\hat{d}}_{zj}^{\left(L\right)} & \vdots \\ {\hat{d}}_{L1}^{\left(L\right)} & \cdots & {\hat{d}}_{LJ}^{\left(L\right)} \end{array}\right],H_{hl,hm}=\left[\begin{array}{ccc} -{\hat{b}}_{11}^{\left(L\right)} & \cdots & -{\hat{b}}_{1R}^{\left(L\right)}\\ \vdots & -{\hat{b}}_{zr}^{\left(L\right)} & \vdots \\ -{\hat{b}}_{L1}^{\left(L\right)} & \cdots & -{\hat{b}}_{LR}^{\left(L\right)} \end{array}\right],H_{hl,hp}=\left[\begin{array}{ccc} {\hat{a}}_{11}^{\left(L\right)} & \cdots & {\hat{a}}_{1I}^{\left(L\right)}\\ \vdots & {\hat{a}}_{zi}^{\left(L\right)} & \vdots \\ {\hat{a}}_{L1}^{\left(L\right)} & \cdots & {\hat{a}}_{LI}^{\left(L\right)} \end{array}\right],\\ H_{hl,hc}=\left[\begin{array}{ccc} {\hat{\zeta }}_{11}^{\left(L\right)} & \cdots & {\hat{\zeta }}_{1I'I''}^{\left(L\right)}\\ \vdots & {\hat{\zeta }}_{z(I''(i'-1)+i'')}^{\left(L\right)} & \vdots \\ {\hat{\zeta }}_{L1}^{\left(L\right)} & \cdots & {\hat{\zeta }}_{LI'I''}^{\left(L\right)} \end{array}\right],H_{hl,hl}=\left[\begin{array}{ccc} {\hat{c}}_{11}^{\left(L\right)} & \cdots & {\hat{c}}_{1L}^{\left(L\right)}\\ \vdots & {\hat{c}}_{zl}^{\left(L\right)} & \vdots \\ {\hat{c}}_{L1}^{\left(L\right)} & \cdots & {\hat{c}}_{LL}^{\left(L\right)} \end{array}\right] \end{array}$$

where ${\hat{\alpha }}_{in}^{\left(h\right)}$ and ${\hat{\gamma }}_{ij}^{\left(h\right)}$ are the corresponding components in ${\hat{\theta }}_{i}^{HP}$ obtained by solving parameter estimation Eq (12) and system order detection Eq (13); ${\hat{\alpha }}_{jm}^{\left(v\right)}$ and ${\hat{\gamma }}_{ji}^{\left(v\right)}$ are the corresponding components in ${\hat{\theta }}_{j}^{VP}$ obtained by solving parameter estimation equation (S5) and system order detection equation (S31); ${\hat{a}}_{ki}^{\left(h\right)},\;{\hat{\zeta }}_{k(I''(i'-1)+i'')}^{\left(h\right)},\;-{\hat{b}}_{kr}^{\left(h\right)},\;{\hat{c}}_{kl}^{\left(h\right)},\;{\hat{d}}_{kj}^{\left(h\right)},\;\mathrm{and}\;-{\hat{e}}_{kq}^{\left(h\right)}$ are the corresponding components in ${\hat{\theta }}_{k}^{HG}$ obtained by solving parameter estimation equation (S10) and system order detection equation (S32); ${\hat{a}}_{si}^{\left(v\right)},\;{\hat{\zeta }}_{s(I''(i'-1)+i'')}^{\left(v\right)},\;-{\hat{b}}_{sr}^{\left(v\right)},\;{\hat{c}}_{sl}^{\left(v\right)},\;{\hat{d}}_{sj}^{\left(v\right)},\;\mathrm{and}\;-{\hat{e}}_{sq}^{\left(v\right)}$ are the corresponding components in ${\hat{\theta }}_{s}^{VG}$ obtained by solving parameter estimation equation (S15) and system order detection equation (S33); ${\hat{a}}_{zi}^{\left(L\right)},\;{\hat{\zeta }}_{z(I''(i'-1)+i'')}^{\left(L\right)},\;-{\hat{b}}_{zr}^{\left(L\right)},\;{\hat{c}}_{zl}^{\left(L\right)},\;{\hat{d}}_{zj}^{\left(L\right)},\;\mathrm{and}\;-{\hat{e}}_{zq}^{\left(L\right)}$ are the corresponding components in ${\hat{\theta }}_{z}^{HL}$ obtained by solving parameter estimation equation (S20) and system order detection equation (S34); ${\hat{\bar{a}}}_{fi}^{\left(h\right)},{\hat{\bar{\zeta }}}_{f(I''(i'-1)+i'')}^{\left(h\right)},-{\hat{\bar{b}}}_{fr}^{\left(h\right)},{\hat{\bar{d}}}_{fj}^{\left(h\right)},\mathrm{and}-{\hat{\bar{e}}}_{fq}^{\left(h\right)}$ are the corresponding components in ${\hat{\tau }}_{f}^{HM}$ obtained by solving parameter estimation equation (S25) and system order detection equation (S35); and ${\hat{\bar{a}}}_{ui}^{\left(v\right)},\;{\hat{\bar{\zeta }}}_{u(I''(i'-1)+i'')}^{\left(v\right)},\;-{\hat{\bar{b}}}_{ur}^{\left(v\right)},\;{\hat{\bar{d}}}_{uj}^{\left(v\right)},\;\mathrm{and}\;-{\hat{\bar{e}}}_{uq}^{\left(v\right)}$ are the corresponding components in ${\hat{\tau }}_{u}^{VM}$ obtained by solving parameter estimation equation (S30) and system order detection equation (S36). ${\hat{\alpha }}_{in}^{\left(h\right)}$ and ${\hat{\alpha }}_{jm}^{\left(v\right)}$ indicate the intraspecies interactive abilities of human and EBV PPINs during EBV infection, respectively; ${\hat{\gamma }}_{ij}^{\left(h\right)}$ and ${\hat{\gamma }}_{ji}^{\left(v\right)}$ denote the intraspecies interactive abilities of human-protein *i* and EBV-protein *j* in human and EBV PPINs; ${\hat{a}}_{ki}^{\left(h\right)},\;{\hat{a}}_{si}^{\left(v\right)},\;{\hat{a}}_{zi}^{\left(L\right)},\;{\hat{\bar{a}}}_{fi}^{\left(h\right)},\;\mathrm{and}\;{\hat{\bar{a}}}_{ui}^{\left(v\right)}$ represent the abilities of human-TF *i* to regulate the transcription of human-gene *k*, EBV-gene *s*, human-lncRNA *z*, human-miRNA *f*, and EBV-miRNA *u*, respectively, in human-gene GRN, EBV-gene GRN, human-lncRNA GRN, human-miRNA GRN, and EBV-miRNA GRN during EBV infection, respectively; ${\hat{\zeta }}_{k(I''(i'-1)+i'')}^{\left(h\right)},\;{\hat{\zeta }}_{s(I''(i'-1)+i'')}^{\left(v\right)},\;{\hat{\zeta }}_{z(I''(i'-1)+i'')}^{\left(L\right)},\;{\hat{\bar{\zeta }}}_{f(I''(i'-1)+i'')}^{\left(h\right)},\;\mathrm{and}\;{\hat{\bar{\zeta }}}_{u(I''(i'-1)+i'')}^{\left(v\right)}$ represent the abilities of human-TF complex *i*'::*i*'' to regulate the transcription of human-gene *k*, EBV-gene *s*, human-lncRNA *z*, human-miRNA *f*, and EBV-miRNA *u*, respectively, in human-gene GRN, EBV-gene GRN, human-lncRNA GRN, human-miRNA GRN, and EBV-miRNA GRN during EBV infection, respectively; ${\hat{d}}_{kj}^{\left(h\right)},\;{\hat{d}}_{sj}^{\left(v\right)},\;{\hat{d}}_{zj}^{\left(L\right)},\;{\hat{\bar{d}}}_{fj}^{\left(h\right)},\;\mathrm{and}\;{\hat{\bar{d}}}_{uj}^{\left(v\right)}$ represent the abilities of EBV-TF *s* to regulate the transcription of human-gene *k*, EBV-gene *s*, human-lncRNA *z*, human-miRNA *f*, and EBV-miRNA *u*, respectively, in human-gene GRN, EBV-gene GRN, human-lncRNA GRN, human-miRNA GRN, and EBV-miRNA GRN during EBV infection, respectively; ${\hat{c}}_{kl}^{\left(h\right)},\;{\hat{c}}_{sl}^{\left(v\right)},\;\mathrm{and}\;{\hat{c}}_{zl}^{\left(L\right)}$ represent the abilities of human-lncRNA *z* to regulate the transcription of human-gene *k*, EBV-gene *s*, and human-lncRNA *z*, respectively, in human-gene GRN, EBV-gene GRN, and human-lncRNA GRN during EBV infection, respectively; $‑{\hat{b}}_{kr}^{\left(h\right)},-{\hat{b}}_{sr}^{\left(v\right)},-{\hat{b}}_{zr}^{\left(L\right)},-{\hat{\bar{b}}}_{fr}^{\left(h\right)},\;\mathrm{and}-{\hat{\bar{b}}}_{ur}^{\left(v\right)}$ correspond to the abilities of human-miRNA *r* to inhibit human-gene *k*, EBV-gene *s*, human-lncRNA *z*, human-miRNA *f*, and EBV-miRNA *u*, respectively, in human-gene GRN, EBV-gene GRN, human-lncRNA GRN, human-miRNA GRN, and EBV-miRNA GRN during EBV infection, respectively; and $‑{\hat{e}}_{kq}^{\left(h\right)},-{\hat{e}}_{sq}^{\left(v\right)},-{\hat{e}}_{zq}^{\left(L\right)},-{\hat{\bar{e}}}_{fq}^{\left(h\right)},\;\mathrm{and}-{\hat{\bar{e}}}_{uq}^{\left(v\right)}$ represent the abilities of EBV-miRNA *q* to inhibit human-gene *k*, EBV-gene *s*, human-lncRNA *z*, human-miRNA *f*, and EBV-miRNA *u*, respectively, in human-gene GRN, EBV-gene GRN, human-lncRNA GRN, human-miRNA GRN, and EBV-miRNA GRN during EBV infection, respectively.

The estimated weights (i.e., parameters) of the network links in intraspecies PPINs, intraspecies GRNs, interspecies PPINs, and interspecies GRNs therefore constitute the system network matrix *H* of the real GIGEN. In the network matrix *H*, the corresponding parameter is zero if a link does not appear in the candidate GIGEN or has been pruned via the AIC. We then extracted the core network of the real GIGEN by applying PNP to network matrix *H* [129]. PNP is achieved on the basis of the singular value decomposition of *H* in the following equation:

$$H=UDV^{T}$$ (14)

where $U\in {\mathbb{R}}^{\left(2J+2I+Q+R+L\right)\times \left(J+I+Q+R+L+I'I''\right)}$, $V\in {\mathbb{R}}^{\left(J+I+Q+R+L+I'I''\right)\times \left(J+I+Q+R+L+I'I''\right)}$, and *D* = **diag** (*d*_1_,…,*d*_*s*_,…,*d*_*J*+*I*+*Q*+*R*+*L*+*I'I''*_), in which the *J*+*I*+*Q*+*R*+*L*+*I'I''* singular values of *H* are in descending order, i.e., *d*_1_ ≥…≥ *d*_*s*_ ≥…≥ *d*_*J*+*I*+*Q*+*R*+*L*+*I'I''*_. Notably, **diag** (*d*_1_, *d*_2_) indicates the diagonal matrix of *d*_1_ and *d*_2_. Moreover, we defined the eigenexpression fraction (*E*_*s*_) for the normalization of singular values as follows:

$$E_{s}=\frac{d_{s}{}^{2}}{\sum_{s=1}^{J+I+Q+R+L+I'I''}d_{s}{}^{2}}$$ (15)

It is necessary to maintain the system energy of the whole network structure. Therefore, we chose the top *K* singular vectors of network matrix *H* with the minimum *K* resulting in $\sum_{s=1}^{K}E_{s}\geq 85\%$ to represent at least 85% of the energy of the core network structure of the GIGEN, which constituted the top *K* principal components. Next, we defined the projection (*T*) of network matrix *H* for the top *K* singular vectors of *U* and *V* as follows:

$$\begin{array}{l} T_{R}(r,s)=h_{r,:}\times v_{:,s}\;\mathrm{and}\;T_{L}(l,s)=h_{:,l}^{T}\times u_{:,s},\;\mathrm{for}\;s=1,\cdots ,K\\ r=1,\cdots ,(2J+2I+Q+R+L),\;\mathrm{and}\;l=1,\cdots ,(J+I+Q+R+L+I'I'') \end{array}$$ (16)

where *h*_*r*,:_, *v*_:,*s*_, *h*_:,*l*_, and *u*_:,*s*_ denote the *r*th row of *H*, the *s*th column of *V*, the *l*th column of *H*, and the *s*th column of *U*, respectively. Finally, we defined and applied the 2-norm projection value of each node, including gene, miRNA, lncRNA, protein, and protein complex in the real GIGEN for the top *K* right singular vectors and the top *K* left singular vectors as follows:

DR(r)=[∑s=1KTR(r,s)2]12andDL(l)=[∑s=1KTL(l,s)2]12forr=1,…,(2J+2I+Q+R+L)andl=1,…,(J+I+Q+R+L+I'I'') (17)

It is implied that if the *D*_*R*_(*r*) or *D*_*L*_(*l*) projection values approach zero, the contributions of the corresponding *r*th or *l*th nodes, respectively, are insignificant and almost independent of the core network structure composed of the top *K* singular vectors. Consequently, we built core networks comprising the core proteins, genes, and miRNAs by selecting the proteins, genes and miRNAs with the top projection values in (17) from the TF receptors and their associated genes and miRNAs. The human and EBV HVCNs extracted at the first and second infection stage from the real GIGENs by the above PNP method are presented in Figs 5 and 6, respectively, and the information concerning the number of nodes and edges of the HVCNs at the first and second infection stage are exhibited in Table 3A and 3B, respectively.

## Supporting information

S1 Text System identification approach for the other Eqs (2)–(7) in GIGEN. (DOCX)

S2 Text System order detection scheme for the other Eqations (S4), (S9), (S14), (S19), (S24), and (S29) in GIGEN. (DOCX)

S1 Table Each edge in the real GIGENs and HVCNs and virus-interacting host pathways. S1A Table and S1B Table show each edge of the real GIGENs and HVCNs. S1C Table reveals the *p*-value of each edge in the PPINs of the HVCNs. S1D Table is the identified virus-interacting host pathways at the first and second infection stages.
(XLSX)
