# Supplementary material for: Investigating genetic-and-epigenetic networks, and the cellular mechanisms occurring in Epstein–Barr virus-infected human B lymphocytes via big data mining and genome-wide two-sided NGS data identification
Source: PLoS One. 2018 Aug 22;13(8):e0202537. doi: 10.1371/journal.pone.0202537 (PMC6105016; doi:10.1371/journal.pone.0202537)
Supplement: S1 Text — (DOCX) [file pone.0202537.s001.docx]

**S1 Text:**

Similarly, EBV PPIN equation (2) was rewritten as the following linear regression:

| $\begin{matrix} p_{j}^{(v)}(t+1)=\left[\begin{matrix} p_{1}^{(v)}(t)p_{j}^{(v)}(t) & \cdots& p_{M_{j}}^{(v)}(t)p_{j}^{(v)}(t) & p_{1}^{(h)}(t)p_{j}^{(v)}(t) \end{matrix}\right. \\ \text{ }\left.\begin{matrix} \cdots& p_{I_{j}}^{(h)}(t)p_{j}^{(v)}(t) & g_{j}^{(v)}(t) & p_{j}^{(v)}(t) & 1 \end{matrix}\right]\left[\begin{matrix} \alpha_{j1}^{(v)} \\ \vdots\\ \alpha_{jM_{j}}^{(v)} \\ \gamma_{j1}^{(v)} \\ \vdots\\ \gamma_{jI_{j}}^{(v)} \\ \lambda_{j}^{(v)} \\ 1-\sigma_{j}^{(v)} \\ \beta_{j}^{(v)} \end{matrix}\right] \\ \text{ }+\varepsilon_{j}^{(v)}(t),\text{ for }j=1,2,\ldots,J,\text{ }-\sigma_{j}^{(v)}\leq0,\text{ and }\lambda_{j}^{(v)}\geq0 \end{matrix}$ | (S1) |
| --- | --- |

which can be simplified to:

|  | (S2) |
| --- | --- |

where *ϕVP j*(*t*) indicates the regression vector obtained from the corresponding expression data, and *θVP j* denotes the unknown interaction parameter vector of EBV-protein *j* in the EBV PPIN to be estimated. Equation (S2) could be augmented for *Y_j_* data points of EBV-protein *j* as follows:

|  | (S3) |
| --- | --- |

where *Y_j_* is the number of data points of protein expression. Thus, we defined the notations *P(v) j*, Φ*VP j*, and Ξ*VP j* to represent equation (S3) as follows:

| $P_{j}^{(v)}=\Phi_{j}^{VP}\theta_{j}^{VP}+\Xi_{j}^{VP},\text{ for }j=1,2,\ldots,J,\text{ }-\sigma_{j}^{(v)}\leq0,\text{ and }\lambda_{j}^{(v)}\geq0$ | (S4) |
| --- | --- |

where .

Next, we formulated the parameter estimation of *θVP j* as the following constrained least square equation:

| $\min_{\theta_{j}^{VP}} \frac{1}{2}\left\Vert\Phi_{j}^{VP}\theta_{j}^{VP}-P_{j}^{(v)}\right\Vert_{2}^{2}\text{ subject to }A\theta_{j}^{VP}\leq b$ | (S5) |
| --- | --- |

where $A=\overbrace{\left[\begin{matrix} 0 & \cdots& 0 \\ 0 & \cdots& 0 \end{matrix}\right.}^{M_{j}+I_{j}}\left.\begin{matrix} -1 & 0 & 0 \\ 0 & 1 & 0 \end{matrix}\right],\text{ }b=\left[\begin{matrix} 0 \\ 1 \end{matrix}\right]$.

By solving equation (S5), we acquired the interaction parameters in EBV PPIN equation (2), and concurrently ensured that the EBV-protein translation rate *λ(v) j* was a non-negative value and the EBV-protein degradation rate -*σ(v) j* was a non-positive value; that is to say *λ(v) j*≥ 0 and -*σ(v) j*≤ 0.

Because the same process occurs in PPIN, human-gene GRN equation (3) can be rewritten as:

| $\begin{matrix} g_{k}^{(h)}(t+1)=\left[\begin{matrix} p_{1}^{(h)}(t) & \cdots& p_{I_{k}}^{(h)}(t) & p_{1}^{(h)}(t)p_{1}^{(h)}(t) & \cdots& p_{I_{k}^{'}}^{(h)}(t)p_{I_{k}^{''}}^{(h)}(t) & \end{matrix}\right. \\ \text{ }w_{1}^{(h)}(t)g_{k}^{(h)}(t)\begin{matrix} \cdots& w_{R_{k}}^{(h)}(t)g_{k}^{(h)}(t) & o_{1}^{(h)}(t) & \cdots& o_{L_{k}}^{(h)}(t) & & & & \end{matrix} \\ \left.\begin{matrix} \text{ }p_{1}^{(v)}(t)\text{ }\cdots\text{ }p_{J_{k}}^{(v)}(t)\text{ }w_{1}^{(v)}(t)g_{k}^{(h)}(t)\text{ }\cdots& w_{Q_{k}}^{(v)}(t)g_{k}^{(h)}(t) & g_{k}^{(h)}(t) & 1 \end{matrix}\right]\left[\begin{matrix} a_{k1}^{(h)} \\ \vdots\\ a_{kI_{k}}^{(h)} \\ \zeta_{k11}^{(h)} \\ \vdots\\ \zeta_{kI_{k}^{'}I_{k}^{''}}^{(h)} \\ -b_{k1}^{(h)} \\ \vdots\\ -b_{kR_{k}}^{(h)} \\ c_{k1}^{(h)} \\ \vdots\\ c_{kL_{k}}^{(h)} \\ d_{k1}^{(h)} \\ \vdots\\ d_{kJ_{k}}^{(h)} \\ -e_{k1}^{(h)} \\ \vdots\\ -e_{kQ_{k}}^{(h)} \\ 1-\mu_{k}^{(h)} \\ \delta_{k}^{(h)} \end{matrix}\right] \\ \text{ }+\omega_{k}^{(h)}(t),\text{ for }k=1,2,\ldots,K,\text{ }-b_{kr}^{(h)}\leq0,\text{ }-e_{kq}^{(h)}\leq0,\text{ and }-\mu_{k}^{(h)}\leq0 \end{matrix}$ | (S6) |
| --- | --- |

which can be simplified to:

|  | (S7) |
| --- | --- |

where *ϕHG k*(*t*) indicates the regression vector obtained from the corresponding expression data, and *θHG k* denotes the unknown parameter vector of human-gene *k* in the human-gene GRN to be estimated. Equation (S7) can be augmented for *Y_k_* data points of human-gene *k* as follows:

|  | (S8) |
| --- | --- |

where *Y_k_* is the number of data points of gene expression. Thus, we defined the notations *G(h) k*, Φ*HG k*, and Ξ*HG k* to represent equation (S8) as follows:

| $G_{k}^{(h)}=\Phi_{k}^{HG}\theta_{k}^{HG}+\Xi_{k}^{HG},\text{ for }k=1,2,\ldots,K,\text{ }-b_{kr}^{(h)}\leq0,\text{ }-e_{kq}^{(h)}\leq0,\text{ and }-\mu_{k}^{(h)}\leq0$ | (S9) |
| --- | --- |

where .

Next, we formulated the parameter estimation of *θHG k* as the following constrained least square equation:

| $\min_{\theta_{k}^{HG}} \frac{1}{2}\left\Vert\Phi_{k}^{HG}\theta_{k}^{HG}-G_{k}^{(h)}\right\Vert_{2}^{2}\text{ subject to }A\theta_{k}^{HG}\leq b$ | (S10) |
| --- | --- |

where $A=\overbrace{\left[\begin{matrix} 0 & \cdots& 0 \\ \vdots& \ddots& \vdots\\ 0 & \cdots& 0 \\ 0 & \cdots& 0 \\ \vdots& \ddots& \vdots\\ 0 & \cdots& 0 \\ 0 & \cdots& 0 \end{matrix}\right.}^{I_{k}+I_{k}^{'}I_{k}^{''}}\overbrace{\begin{matrix} 1 & \cdots& 0 \\ \vdots& \ddots& \vdots\\ 0 & \cdots& 1 \\ 0 & \cdots& 0 \\ \vdots& \ddots& \vdots\\ 0 & \cdots& 0 \\ 0 & \cdots& 0 \end{matrix}}^{R_{k}}\overbrace{\begin{matrix} 0 & \cdots& 0 \\ \vdots& \ddots& \vdots\\ 0 & \cdots& 0 \\ 0 & \cdots& 0 \\ \vdots& \ddots& \vdots\\ 0 & \cdots& 0 \\ 0 & \cdots& 0 \end{matrix}}^{L_{k}+J_{k}}\overbrace{\begin{matrix} 0 & \cdots& 0 \\ \vdots& \ddots& \vdots\\ 0 & \cdots& 0 \\ 1 & \cdots& 0 \\ \vdots& \ddots& \vdots\\ 0 & \cdots& 1 \\ 0 & \cdots& 0 \end{matrix}}^{Q_{k}}\left.\begin{matrix} 0 & 0 \\ \vdots& \vdots\\ 0 & 0 \\ 0 & 0 \\ \vdots& \vdots\\ 0 & 0 \\ 1 & 0 \end{matrix}\right],\text{ }b=\left[\begin{matrix} 0 \\ \vdots\\ 0 \\ 0 \\ \vdots\\ 0 \\ 1 \end{matrix}\right]$.

By solving equation (S10), we acquired the regulatory parameters in human-gene GRN equation (3), and concurrently ensured that the human-miRNA repression ability -*b(h) kr* was a non-positive value, the EBV-miRNA repression ability -*e(h) kq* was a non-positive value, and the human-gene degradation rate -*μ(h) k* was a non-positive value; that is to say -*b(h) kr*≤ 0, -*e(h) kq*≤ 0, and -*μ(h) k*≤ 0.

Similarly, EBV-gene GRN equation (4) is revealed in the following linear regression:

| $\begin{matrix} g_{s}^{(v)}(t+1)=\left[\begin{matrix} p_{1}^{(h)}(t) & \cdots& p_{I_{s}}^{(h)}(t) & p_{1}^{(h)}(t)p_{1}^{(h)}(t) & \cdots& p_{I_{s}^{'}}^{(h)}(t)p_{I_{s}^{''}}^{(h)}(t) & \end{matrix}\right. \\ \text{ }w_{1}^{(h)}(t)g_{s}^{(v)}(t)\text{ }\begin{matrix} \cdots& w_{R_{s}}^{(h)}(t)g_{s}^{(v)}(t) & o_{1}^{(h)}(t) & \cdots& o_{L_{s}}^{(h)}(t) & & & & \end{matrix} \\ \text{ }p_{1}^{(v)}(t)\text{ }\cdots\text{ }p_{J_{s}}^{(v)}(t)\text{ }w_{1}^{(v)}(t)g_{s}^{(v)}(t)\left.\begin{matrix} \text{ }\cdots& w_{Q_{s}}^{(v)}(t)g_{s}^{(v)}(t) & g_{s}^{(v)}(t) & 1 \end{matrix}\right]\left[\begin{matrix} a_{s1}^{(v)} \\ \vdots\\ a_{sI_{s}}^{(v)} \\ \zeta_{s11}^{(v)} \\ \vdots\\ \zeta_{sI_{s}^{'}I_{s}^{''}}^{(v)} \\ -b_{s1}^{(v)} \\ \vdots\\ -b_{sR_{s}}^{(v)} \\ c_{s1}^{(v)} \\ \vdots\\ c_{sL_{s}}^{(v)} \\ d_{s1}^{(v)} \\ \vdots\\ d_{sJ_{s}}^{(v)} \\ -e_{s1}^{(v)} \\ \vdots\\ -e_{sQ_{s}}^{(v)} \\ 1-\mu_{s}^{(v)} \\ \delta_{s}^{(v)} \end{matrix}\right]\text{ } \\ \text{ }+\omega_{s}^{(v)}(t),\text{for }s=1,2,\ldots,S,\text{ }-b_{sr}^{(v)}\leq0,\text{ }-e_{sq}^{(v)}\leq0,\text{ and}-\mu_{s}^{(v)}\leq0 \end{matrix}$ | (S11) |
| --- | --- |

which can be simplified to:

|  | (S12) |
| --- | --- |

where *ϕVG s*(*t*) indicates the regression vector obtained from the corresponding expression data, and *θVG s* denotes the unknown parameter vector of EBV-gene *s* in the EBV-gene GRN to be estimated. Equation (S12) could be augmented for *Y_s_* data points of EBV-gene *s* as follows:

|  | (S13) |
| --- | --- |

where *Y_s_* is the number of data points of gene expression. Thus, we defined the notations *G(v) s*, Φ*VG s*, and Ξ*VG s* to represent equation (S13) as follows:

| $G_{s}^{(v)}=\Phi_{s}^{VG}\theta_{s}^{VG}+\Xi_{s}^{VG},\text{ for }s=1,2,\ldots,S,\text{ }-b_{sr}^{(v)}\leq0,\text{ }-e_{sq}^{(v)}\leq0,\text{ and}-\mu_{s}^{(v)}\leq0$ | (S14) |
| --- | --- |

where .

Next, we formulated the parameter estimation of *θVG s* as the following constrained least square equation:

| $\min_{\theta_{s}^{VG}} \frac{1}{2}\left\Vert\Phi_{s}^{VG}\theta_{s}^{VG}-G_{s}^{(v)}\right\Vert_{2}^{2}\text{ subject to }A\theta_{s}^{VG}\leq b$ | (S15) |
| --- | --- |

where $A=\overbrace{\left[\begin{matrix} 0 & \cdots& 0 \\ \vdots& \ddots& \vdots\\ 0 & \cdots& 0 \\ 0 & \cdots& 0 \\ \vdots& \ddots& \vdots\\ 0 & \cdots& 0 \\ 0 & \cdots& 0 \end{matrix}\right.}^{I_{s}+I_{s}^{'}I_{s}^{''}}\overbrace{\begin{matrix} 1 & \cdots& 0 \\ \vdots& \ddots& \vdots\\ 0 & \cdots& 1 \\ 0 & \cdots& 0 \\ \vdots& \ddots& \vdots\\ 0 & \cdots& 0 \\ 0 & \cdots& 0 \end{matrix}}^{R_{s}}\overbrace{\begin{matrix} 0 & \cdots& 0 \\ \vdots& \ddots& \vdots\\ 0 & \cdots& 0 \\ 0 & \cdots& 0 \\ \vdots& \ddots& \vdots\\ 0 & \cdots& 0 \\ 0 & \cdots& 0 \end{matrix}}^{L_{s}+J_{s}}\overbrace{\begin{matrix} 0 & \cdots& 0 \\ \vdots& \ddots& \vdots\\ 0 & \cdots& 0 \\ 1 & \cdots& 0 \\ \vdots& \ddots& \vdots\\ 0 & \cdots& 1 \\ 0 & \cdots& 0 \end{matrix}}^{Q_{s}}\left.\begin{matrix} 0 & 0 \\ \vdots& \vdots\\ 0 & 0 \\ 0 & 0 \\ \vdots& \vdots\\ 0 & 0 \\ 1 & 0 \end{matrix}\right],\text{ }b=\left[\begin{matrix} 0 \\ \vdots\\ 0 \\ 0 \\ \vdots\\ 0 \\ 1 \end{matrix}\right]$.

By solving equation (S15), we acquired the regulatory parameters in EBV-gene GRN equation (4), and concurrently ensured that the human-miRNA repression ability -*b(v) sr* was a non-positive value, the EBV-miRNA repression ability -*e(v) sq* was a non-positive value, and the EBV-gene degradation rate -*μ(v) s* was a non-positive value; that is to say -*b(v) sr*≤ 0, -*e(v) sq*≤ 0, and -*μ(v) s*≤ 0.

Similarly, human-lncRNA GRN equation (5) can be rewritten as:

| $\begin{matrix} g_{z}^{(L)}(t+1)=\left[\begin{matrix} p_{1}^{(h)}(t) & \cdots& p_{I_{z}}^{(h)}(t) & p_{1}^{(h)}(t)p_{1}^{(h)}(t) & \cdots& p_{I_{z}^{'}}^{(h)}(t)p_{I_{z}^{''}}^{(h)}(t) & \end{matrix}\right. \\ \text{ }w_{1}^{(h)}(t)g_{z}^{(L)}(t)\text{ }\begin{matrix} \cdots& w_{R_{z}}^{(h)}(t)g_{z}^{(L)}(t) & o_{1}^{(h)}(t) & \cdots& o_{L_{z}}^{(h)}(t) & & & & \end{matrix} \\ p_{1}^{(v)}(t)\text{ }\cdots\text{ }p_{J_{z}}^{(v)}(t)\text{ }w_{1}^{(v)}(t)g_{z}^{(L)}(t)\text{ }\left.\begin{matrix} \cdots& w_{Q_{z}}^{(v)}(t)g_{z}^{(L)}(t) & g_{z}^{(L)}(t) & 1 \end{matrix}\right]\left[\begin{matrix} a_{z1}^{(L)} \\ \vdots\\ a_{zI_{z}}^{(L)} \\ \zeta_{z11}^{(L)} \\ \vdots\\ \zeta_{zI_{z}^{'}I_{z}^{''}}^{(L)} \\ -b_{z1}^{(L)} \\ \vdots\\ -b_{zR_{z}}^{(L)} \\ c_{z1}^{(L)} \\ \vdots\\ c_{zL_{z}}^{(L)} \\ d_{z1}^{(L)} \\ \vdots\\ d_{zJ_{z}}^{(L)} \\ -e_{z1}^{(L)} \\ \vdots\\ -e_{zQ_{z}}^{(L)} \\ 1-\mu_{z}^{(L)} \\ \delta_{z}^{(L)} \end{matrix}\right]\text{ } \\ \text{ }+\omega_{z}^{(L)}(t),\text{ for }z=1,2,\ldots,Z,\text{ }-b_{zr}^{(L)}\leq0,\text{ }-e_{zq}^{(L)}\leq0,\text{ and}-\mu_{z}^{(L)}\leq0 \end{matrix}$ | (S16) |
| --- | --- |

which can be simplified to:

|  | (S17) |
| --- | --- |

where *ϕHL z*(*t*) indicates the regression vector obtained from the corresponding expression data, and *θHL z* denotes the unknown parameter vector of human-lncRNA *z* in the human-lncRNA GRN to be estimated. Equation (S17) could be augmented for *Y_z_* data points of human-lncRNA *z* as follows:

|  | (S18) |
| --- | --- |

where *Y_z_* is the number of data points of gene expression. Thus, we defined the notations *G(L) z*, Φ*HL z*, and Ξ*HL z* to represent equation (S18) as follows:

| $G_{z}^{(L)}=\Phi_{z}^{HL}\theta_{z}^{HL}+\Xi_{z}^{HL},\text{ for }z=1,2,\ldots,Z,\text{ }-b_{zr}^{(L)}\leq0,\text{ }-e_{zq}^{(L)}\leq0,\text{ and}-\mu_{z}^{(L)}\leq0$ | (S19) |
| --- | --- |

where .

Next, we formulated the parameter estimation of *θHL z* as the following constrained least square equation:

| $\min_{\theta_{z}^{HL}} \frac{1}{2}\left\Vert\Phi_{z}^{HL}\theta_{z}^{HL}-G_{z}^{(L)}\right\Vert_{2}^{2}\text{ subject to }A\theta_{z}^{HL}\leq b$ | (S20) |
| --- | --- |

where $A=\overbrace{\left[\begin{matrix} 0 & \cdots& 0 \\ \vdots& \ddots& \vdots\\ 0 & \cdots& 0 \\ 0 & \cdots& 0 \\ \vdots& \ddots& \vdots\\ 0 & \cdots& 0 \\ 0 & \cdots& 0 \end{matrix}\right.}^{I_{z}+I_{z}^{'}I_{z}^{''}}\overbrace{\begin{matrix} 1 & \cdots& 0 \\ \vdots& \ddots& \vdots\\ 0 & \cdots& 1 \\ 0 & \cdots& 0 \\ \vdots& \ddots& \vdots\\ 0 & \cdots& 0 \\ 0 & \cdots& 0 \end{matrix}}^{R_{z}}\overbrace{\begin{matrix} 0 & \cdots& 0 \\ \vdots& \ddots& \vdots\\ 0 & \cdots& 0 \\ 0 & \cdots& 0 \\ \vdots& \ddots& \vdots\\ 0 & \cdots& 0 \\ 0 & \cdots& 0 \end{matrix}}^{L_{z}+J_{z}}\overbrace{\begin{matrix} 0 & \cdots& 0 \\ \vdots& \ddots& \vdots\\ 0 & \cdots& 0 \\ 1 & \cdots& 0 \\ \vdots& \ddots& \vdots\\ 0 & \cdots& 1 \\ 0 & \cdots& 0 \end{matrix}}^{Q_{z}}\left.\begin{matrix} 0 & 0 \\ \vdots& \vdots\\ 0 & 0 \\ 0 & 0 \\ \vdots& \vdots\\ 0 & 0 \\ 1 & 0 \end{matrix}\right],\text{ }b=\left[\begin{matrix} 0 \\ \vdots\\ 0 \\ 0 \\ \vdots\\ 0 \\ 1 \end{matrix}\right]$.

By solving equation (S20), we acquired the regulatory parameters in human-lncRNA GRN equation (5), and concurrently ensured that the human-miRNA repression ability -*b(L) zr* was a non-positive value, the EBV-miRNA repression ability -*e(L) zq* was a non-positive value, and the human-lncRNA degradation rate -*μ(L) z* was a non-positive value; that is to say -*b(L) zr*≤ 0, -*e(L) zq*≤ 0, and -*μ(L) z*≤ 0.

Because the same process occurs in human-gene GRN equation (3), human-miRNA GRN equation (6) can be written as:

| $\begin{matrix} x_{f}^{(h)}(t+1)=\left[\begin{matrix} p_{1}^{(h)}(t) & \cdots& p_{I_{f}}^{(h)}(t) & p_{1}^{(h)}(t)p_{1}^{(h)}(t) & \cdots& p_{I_{f}^{'}}^{(h)}(t)p_{I_{f}^{''}}^{(h)}(t) & \end{matrix}\right. \\ \text{ }w_{1}^{(h)}(t)x_{f}^{(h)}(t)\text{ }\begin{matrix} \cdots& w_{R_{f}}^{(h)}(t)x_{f}^{(h)}(t) & p_{1}^{(v)}(t) & \cdots& p_{J_{f}}^{(v)}(t) & \end{matrix} \\ \left.\text{ }w_{1}^{(v)}(t)x_{f}^{(h)}(t)\text{ }\begin{matrix} \cdots& w_{Q_{f}}^{(v)}(t)x_{f}^{(h)}(t) & x_{f}^{(h)}(t) & 1 \end{matrix}\right]\left[\begin{matrix} \bar{a}_{f1}^{(h)} \\ \vdots\\ \bar{a}_{fI_{f}}^{(h)} \\ \bar{\zeta}_{f11}^{(h)} \\ \vdots\\ \bar{\zeta}_{fI_{f}^{'}I_{f}^{''}}^{(h)} \\ -\bar{b}_{f1}^{(h)} \\ \vdots\\ -\bar{b}_{fR_{f}}^{(h)} \\ \bar{d}_{f1}^{(h)} \\ \vdots\\ \bar{d}_{fJ_{f}}^{(h)} \\ -\bar{e}_{f1}^{(h)} \\ \vdots\\ -\bar{e}_{fQ_{f}}^{(h)} \\ 1-\rho_{f}^{(h)} \\ \eta_{f}^{(h)} \end{matrix}\right]+\psi_{f}^{(h)}(t),\text{ } \\ \text{ for }f=1,2,\ldots,F,\text{ }-\bar{b}_{fr}^{(h)}\leq0,\text{ }-\bar{e}_{fq}^{(h)}\leq0,\text{ and}-\rho_{f}^{(h)}\leq0 \end{matrix}$ | (S21) |
| --- | --- |

which can be simplified to:

|  | (S22) |
| --- | --- |

where *φHM f*(*t*) indicates the regression vector obtained from the corresponding expression data, and *τHM f* denotes the unknown parameter vector of human-miRNA *f* in the human-miRNA GRN to be estimated. Equation (S22) could be augmented for *Y_f_* data points of human-miRNA *f* as follows:

|  | (S23) |
| --- | --- |

where *Y_f_* is the number of data points of gene expression. Thus, we defined the notations *X(h) f*, Θ*HM f*, and Γ*HM f* to represent equation (S23) as follows:

| $\begin{matrix} X_{f}^{(h)}=\Theta_{f}^{HM}\tau_{f}^{HM}+\Gamma_{f}^{HM},\text{ for }f=1,2,\ldots,F,\text{ }-\bar{b}_{fr}^{(h)}\leq0,\text{ }-\bar{e}_{fq}^{(h)}\leq0,\text{ } \\ \text{and}-\rho_{f}^{(h)}\leq0 \end{matrix}$ | (S24) |
| --- | --- |

where .

Next, we formulated the parameter estimation of *τHM f* as the following constrained least square equation:

| $\min_{\tau_{f}^{HM}} \frac{1}{2}\left\Vert\Theta_{f}^{HM}\tau_{f}^{HM}-X_{f}^{(h)}\right\Vert_{2}^{2}\text{ subject to }A\tau_{f}^{HM}\leq b$ | (S25) |
| --- | --- |

where $A=\overbrace{\left[\begin{matrix} 0 & \cdots& 0 \\ \vdots& \ddots& \vdots\\ 0 & \cdots& 0 \\ 0 & \cdots& 0 \\ \vdots& \ddots& \vdots\\ 0 & \cdots& 0 \\ 0 & \cdots& 0 \end{matrix}\right.}^{I_{f}+I_{f}^{'}I_{f}^{''}}\overbrace{\begin{matrix} 1 & \cdots& 0 \\ \vdots& \ddots& \vdots\\ 0 & \cdots& 1 \\ 0 & \cdots& 0 \\ \vdots& \ddots& \vdots\\ 0 & \cdots& 0 \\ 0 & \cdots& 0 \end{matrix}}^{R_{f}}\overbrace{\begin{matrix} 0 & \cdots& 0 \\ \vdots& \ddots& \vdots\\ 0 & \cdots& 0 \\ 0 & \cdots& 0 \\ \vdots& \ddots& \vdots\\ 0 & \cdots& 0 \\ 0 & \cdots& 0 \end{matrix}}^{J_{f}}\overbrace{\begin{matrix} 0 & \cdots& 0 \\ \vdots& \ddots& \vdots\\ 0 & \cdots& 0 \\ 1 & \cdots& 0 \\ \vdots& \ddots& \vdots\\ 0 & \cdots& 1 \\ 0 & \cdots& 0 \end{matrix}}^{Q_{f}}\left.\begin{matrix} 0 & 0 \\ \vdots& \vdots\\ 0 & 0 \\ 0 & 0 \\ \vdots& \vdots\\ 0 & 0 \\ 1 & 0 \end{matrix}\right],\text{ }b=\left[\begin{matrix} 0 \\ \vdots\\ 0 \\ 0 \\ \vdots\\ 0 \\ 1 \end{matrix}\right]$.

By solving equation (S25), we acquired the regulatory parameters in human-miRNA GRN equation (6), and concurrently ensured that the human-miRNA repression ability $-\bar{b}_{fr}^{(h)}$ was a non-positive value, the EBV-miRNA repression ability $-\bar{e}_{fq}^{(h)}$ was a non-positive value, and the human-miRNA degradation rate -*ρ(h) f* was a non-positive value; that is to say $-\bar{b}_{fr}^{(h)}$≤ 0, $-\bar{e}_{fq}^{(h)}$≤ 0, and -*ρ(h) f*≤ 0.

Similarly, we can rewrite EBV-miRNA GRN equation (7) as the following linear regression:

| $\begin{matrix} x_{u}^{(v)}(t+1)=\left[\begin{matrix} p_{1}^{(h)}(t) & \cdots& p_{I_{u}}^{(h)}(t) & p_{1}^{(h)}(t)p_{1}^{(h)}(t) & \cdots& p_{I_{u}^{'}}^{(h)}(t)p_{I_{u}^{''}}^{(h)}(t) & \end{matrix}\right. \\ \text{ }w_{1}^{(h)}(t)x_{u}^{(v)}(t)\begin{matrix} \cdots& w_{R_{u}}^{(h)}(t)x_{u}^{(v)}(t) & p_{1}^{(v)}(t) & \cdots& p_{J_{u}}^{(v)}(t) & \end{matrix} \\ \left.\text{ }w_{1}^{(v)}(t)x_{u}^{(v)}(t)\text{ }\begin{matrix} \cdots& w_{Q_{u}}^{(v)}(t)x_{u}^{(v)}(t) & x_{u}^{(v)}(t) & 1 \end{matrix}\right]\left[\begin{matrix} \bar{a}_{u1}^{(v)} \\ \vdots\\ \bar{a}_{uI_{u}}^{(v)} \\ \bar{\zeta}_{u11}^{(v)} \\ \vdots\\ \bar{\zeta}_{uI_{u}^{'}I_{u}^{''}}^{(v)} \\ -\bar{b}_{u1}^{(v)} \\ \vdots\\ -\bar{b}_{uR_{u}}^{(v)} \\ \bar{d}_{u1}^{(v)} \\ \vdots\\ \bar{d}_{uJ_{u}}^{(v)} \\ -\bar{e}_{u1}^{(v)} \\ \vdots\\ -\bar{e}_{uQ_{u}}^{(v)} \\ 1-\rho_{u}^{(v)} \\ \eta_{u}^{(v)} \end{matrix}\right]+\psi_{u}^{(v)}(t),\text{ } \\ \text{ for }u=1,2,\ldots,U,\text{ }-\bar{b}_{ur}^{(v)}\leq0,\text{ }-\bar{e}_{uq}^{(v)}\leq0,\text{ and}-\rho_{u}^{(v)}\leq0 \end{matrix}$ | (S26) |
| --- | --- |

which can be simplified to:

|  | (S27) |
| --- | --- |

where *φVM u*(*t*) indicates the regression vector obtained from the corresponding expression data, and *τVM u* denotes the unknown parameter vector of EBV-miRNA *u* in the EBV-miRNA GRN to be estimated. Equation (S27) could be augmented for *Y_u_* data points of EBV-miRNA *u* as follows:

|  | (S28) |
| --- | --- |

where *Y_u_* is the number of data points of gene expression. Thus, we defined the notations *X(v) u*, Θ*VM u*, and Γ*VM u* to represent equation (S28) as follows:

| $X_{u}^{(v)}=\Theta_{u}^{VM}\tau_{u}^{VM}+\Gamma_{u}^{VM},\text{ for }u=1,2,\ldots,U,\text{ }-\bar{b}_{ur}^{(v)}\leq0,\text{ }-\bar{e}_{uq}^{(v)}\leq0,\text{ and}-\rho_{u}^{(v)}\leq0$ | (S29) |
| --- | --- |

where .

Next, we formulated the parameter estimation of *τVM u* as the following constrained least square equation:

| $\min_{\tau_{u}^{VM}} \frac{1}{2}\left\Vert\Theta_{u}^{VM}\tau_{u}^{VM}-X_{u}^{(v)}\right\Vert_{2}^{2}\text{ subject to }A\tau_{u}^{VM}\leq b$ | (S30) |
| --- | --- |

where $A=\overbrace{\left[\begin{matrix} 0 & \cdots& 0 \\ \vdots& \ddots& \vdots\\ 0 & \cdots& 0 \\ 0 & \cdots& 0 \\ \vdots& \ddots& \vdots\\ 0 & \cdots& 0 \\ 0 & \cdots& 0 \end{matrix}\right.}^{I_{u}+I_{u}^{'}I_{u}^{''}}\overbrace{\begin{matrix} 1 & \cdots& 0 \\ \vdots& \ddots& \vdots\\ 0 & \cdots& 1 \\ 0 & \cdots& 0 \\ \vdots& \ddots& \vdots\\ 0 & \cdots& 0 \\ 0 & \cdots& 0 \end{matrix}}^{R_{u}}\overbrace{\begin{matrix} 0 & \cdots& 0 \\ \vdots& \ddots& \vdots\\ 0 & \cdots& 0 \\ 0 & \cdots& 0 \\ \vdots& \ddots& \vdots\\ 0 & \cdots& 0 \\ 0 & \cdots& 0 \end{matrix}}^{J_{u}}\overbrace{\begin{matrix} 0 & \cdots& 0 \\ \vdots& \ddots& \vdots\\ 0 & \cdots& 0 \\ 1 & \cdots& 0 \\ \vdots& \ddots& \vdots\\ 0 & \cdots& 1 \\ 0 & \cdots& 0 \end{matrix}}^{Q_{u}}\left.\begin{matrix} 0 & 0 \\ \vdots& \vdots\\ 0 & 0 \\ 0 & 0 \\ \vdots& \vdots\\ 0 & 0 \\ 1 & 0 \end{matrix}\right],\text{ }b=\left[\begin{matrix} 0 \\ \vdots\\ 0 \\ 0 \\ \vdots\\ 0 \\ 1 \end{matrix}\right]$.

By solving equation (S30), we acquired the regulatory parameters in EBV-miRNA GRN equation (7), and concurrently ensured that the human-miRNA repression ability $-\bar{b}_{ur}^{(v)}$ was a non-positive value, the EBV-miRNA repression ability $-\bar{e}_{uq}^{(v)}$ was a non-positive value, and the EBV-miRNA degradation rate -*ρ(v) u* was a non-positive value; that is to say $-\bar{b}_{ur}^{(v)}$≤ 0, $-\bar{e}_{uq}^{(v)}$≤ 0, and -*ρ(v) u*≤ 0.
