# Supplementary material for: Investigating genetic-and-epigenetic networks, and the cellular mechanisms occurring in Epstein–Barr virus-infected human B lymphocytes via big data mining and genome-wide two-sided NGS data identification
Source: PLoS One. 2018 Aug 22;13(8):e0202537. doi: 10.1371/journal.pone.0202537 (PMC6105016; doi:10.1371/journal.pone.0202537)
Supplement: S2 Text — (DOCX) [file pone.0202537.s002.docx]

**S2 Text:**

Similarly, in the EBV PPIN model (S4), the AIC of EBV-protein *j* can be defined as:

| $\begin{matrix} AIC_{j}^{VP}\left(M_{j},I_{j}\right)=\log\left(\frac{1}{Y_{j}}\left(P_{j}^{(v)}-\Phi_{j}^{VP}\hat{\theta}_{j}^{VP}\right)^{T}\left(P_{j}^{(v)}-\Phi_{j}^{VP}\hat{\theta}_{j}^{VP}\right)\right) \\ \text{ }+\frac{2\left(M_{j}+I_{j}\right)}{Y_{j}} \end{matrix}$ | (S31) |
| --- | --- |

where $\hat{\theta}_{j}^{VP}$ denotes the estimated parameters of EBV-protein *j* obtained from the solution of parameter estimation equation (S5), and the estimated residual error is $\hat{\kappa}_{VP,j}^{2}=\frac{1}{Y_{j}}\left(P_{j}^{(v)}-\Phi_{j}^{VP}\hat{\theta}_{j}^{VP}\right)^{T}\left(P_{j}^{(v)}-\Phi_{j}^{VP}\hat{\theta}_{j}^{VP}\right)$.

The minimum *AICVP j* in (S31) occurred at the real interaction number *M* j*+ *I* j* of the real PPIs of protein *j* in the EBV PPIN. The insignificant PPIs of *M* j* and *I* j* were all pruned away one protein at a time to obtain the real EBV PPIN.

As with the procedure for PPIN, the AIC of human-gene *k* in the human-gene GRN model (S9) can be defined as:

| $\begin{matrix} AIC_{k}^{HG}\left(I_{k},I_{k}^{'}I_{k}^{''},R_{k},L_{k},J_{k},Q_{k}\right)=\log\left(\frac{1}{Y_{k}}\left(G_{k}^{(h)}-\Phi_{k}^{HG}\hat{\theta}_{k}^{HG}\right)^{T}\left(G_{k}^{(h)}-\Phi_{k}^{HG}\hat{\theta}_{k}^{HG}\right)\right) \\ \text{ }+\frac{2\left(I_{k}+I_{k}^{'}I_{k}^{''}+R_{k}+L_{k}+J_{k}+Q_{k}\right)}{Y_{k}} \end{matrix}$ | (S32) |
| --- | --- |

where $\hat{\theta}_{k}^{HG}$ represents the estimated parameters of human-gene *k* obtained from the solution of parameter estimation equation (S10), and the estimated residual error is $\hat{\kappa}_{HG,k}^{2}=\frac{1}{Y_{k}}\left(G_{k}^{(h)}-\Phi_{k}^{HG}\hat{\theta}_{k}^{HG}\right)^{T}\left(G_{k}^{(h)}-\Phi_{k}^{HG}\hat{\theta}_{k}^{HG}\right)$.

The minimum *AICHG k* in (S32) occurred at number *I* k*+*I’ kI’’* k*+*R* k*+*L* k*+*J* k*+*Q* k* of the real gene/miRNA/lncRNA regulations of gene *k* in the human-gene GRN. The insignificant regulations of *I* k*, *I’ kI’’* k*, *R* k*, *L* k*, *J* k*, and *Q* k* were pruned one gene at a time to obtain the real human-gene GRN.

Similarly, the AIC of EBV-gene *s* in EBV-gene GRN model (S14) can be defined as:

| $\begin{matrix} AIC_{s}^{VG}\left(I_{s},I_{s}^{'}I_{s}^{''},R_{s},L_{s},J_{s},Q_{s}\right)=\log\left(\frac{1}{Y_{s}}\left(G_{s}^{(v)}-\Phi_{s}^{VG}\hat{\theta}_{s}^{VG}\right)^{T}\left(G_{s}^{(v)}-\Phi_{s}^{VG}\hat{\theta}_{s}^{VG}\right)\right) \\ \text{ }+\frac{2\left(I_{s}+I_{s}^{'}I_{s}^{''}+R_{s}+L_{s}+J_{s}+Q_{s}\right)}{Y_{s}} \end{matrix}$ | (S33) |
| --- | --- |

where $\hat{\theta}_{s}^{VG}$ means the estimated parameters of EBV-gene *s* obtained from the solution of parameter estimation equation (S15), and the estimated residual error is $\hat{\kappa}_{VG,s}^{2}=\frac{1}{Y_{s}}\left(G_{s}^{(v)}-\Phi_{s}^{VG}\hat{\theta}_{s}^{VG}\right)^{T}\left(G_{s}^{(v)}-\Phi_{s}^{VG}\hat{\theta}_{s}^{VG}\right)$.

The minimum *AICVG s* in (S33) occurred at number *I* s*+*I’ sI’’* s*+*R* s*+*L* s*+*J* s*+*Q* s* of the real gene/miRNA/lncRNA regulations of EBV-gene *s* in the EBV-gene GRN. Therefore, the insignificant regulations in the real regulation orders *I* s*, *I’ sI’’* s*, *R* s*, *L* s*, *J* s*, and *Q* s* were pruned away one gene at a time to obtain the real EBV-gene GRN.

Likewise, the AIC of human-lncRNA *z* in the human-lncRNA GRN model (S19) can be represented by the following equation:

| $\begin{matrix} AIC_{z}^{HL}\left(I_{z},I_{z}^{'}I_{z}^{''},R_{z},L_{z},J_{z},Q_{z}\right)=\log\left(\frac{1}{Y_{z}}\left(G_{z}^{(L)}-\Phi_{z}^{HL}\hat{\theta}_{z}^{HL}\right)^{T}\left(G_{z}^{(L)}-\Phi_{z}^{HL}\hat{\theta}_{z}^{HL}\right)\right) \\ \text{ }+\frac{2\left(I_{z}+I_{z}^{'}I_{z}^{''}+R_{z}+L_{z}+J_{z}+Q_{z}\right)}{Y_{z}} \end{matrix}$ | (S34) |
| --- | --- |

where $\hat{\theta}_{z}^{HL}$ signifies the estimated parameters of human-lncRNA *z* obtained from the solution of parameter estimation equation (S20), and the estimated residual error is $\hat{\kappa}_{HL,z}^{2}=\frac{1}{Y_{z}}\left(G_{z}^{(L)}-\Phi_{z}^{HL}\hat{\theta}_{z}^{HL}\right)^{T}\left(G_{z}^{(L)}-\Phi_{z}^{HL}\hat{\theta}_{z}^{HL}\right)$.

The minimum *AICHL z* in (S34) occurred at number *I* z*+*I’ zI’’* z*+*R* z*+*L* z*+*J* z*+*Q* z* of the real gene/miRNA/lncRNA regulations of human-lncRNA *z* in the human-lncRNA GRN. Therefore, the insignificant regulations in the real regulation orders *I* z*, *I’ zI’’* z*, *R* z*, *L* z*, *J* z*, and *Q* z* were pruned one human-lncRNA at a time to obtain the real human-lncRNA GRN.

Similar to the procedure for human-gene GRN model (S9), the AIC of human-miRNA *f* in human-miRNA GRN model (S24) can be represented as:

| $\begin{matrix} AIC_{f}^{HM}\left(I_{f},I_{f}^{'}I_{f}^{''},R_{f},J_{f},Q_{f}\right)=\log\left(\frac{1}{Y_{f}}\left(X_{f}^{(h)}-\Theta_{f}^{HM}\hat{\tau}_{f}^{HM}\right)^{T}\left(X_{f}^{(h)}-\Theta_{f}^{HM}\hat{\tau}_{f}^{HM}\right)\right) \\ \text{ }+\frac{2\left(I_{f}+I_{f}^{'}I_{f}^{''}+R_{f}+J_{f}+Q_{f}\right)}{Y_{f}} \end{matrix}$ | (S35) |
| --- | --- |

where $\hat{\tau}_{f}^{HM}$ shows the estimated parameters of human-miRNA *f* obtained from the solution of parameter estimation equation (S25), and the estimated residual error is $\hat{\kappa}_{HM,f}^{2}=\frac{1}{Y_{f}}\left(X_{f}^{(h)}-\Theta_{f}^{HM}\hat{\tau}_{f}^{HM}\right)^{T}\left(X_{f}^{(h)}-\Theta_{f}^{HM}\hat{\tau}_{f}^{HM}\right)$.

The minimum *AICHM f* in (S35) occurred at number *I* f*+*I’ fI’’* f*+*R* f*+*J* f*+*Q* f* of the real gene/miRNA regulations of human-miRNA *f* in the human-miRNA GRN. Therefore, the insignificant regulations in the real regulation orders *I* f*, *I’ fI’’* f*, *R* f*, *J* f*, and *Q* f* were pruned one human-miRNA at a time to obtain the real human-miRNA GRN.

Similarly, we can define the AIC of EBV-miRNA *u* in EBV-miRNA GRN model (S29) as follows:

| $\begin{matrix} AIC_{u}^{VM}\left(I_{u},I_{u}^{'}I_{u}^{''},R_{u},J_{u},Q_{u}\right)=\log\left(\frac{1}{Y_{u}}\left(X_{u}^{(v)}-\Theta_{u}^{VM}\hat{\tau}_{u}^{VM}\right)^{T}\left(X_{u}^{(v)}-\Theta_{u}^{VM}\hat{\tau}_{u}^{VM}\right)\right) \\ \text{ }+\frac{2\left(I_{u}+I_{u}^{'}I_{u}^{''}+R_{u}+J_{u}+Q_{u}\right)}{Y_{u}} \end{matrix}$ | (S36) |
| --- | --- |

where $\hat{\tau}_{u}^{VM}$ stands for the estimated parameters of EBV-miRNA *u* obtained from the solution of parameter estimation equation (S30), and the estimated residual error is $\hat{\kappa}_{VM,u}^{2}=\frac{1}{Y_{u}}\left(X_{u}^{(v)}-\Theta_{u}^{VM}\hat{\tau}_{u}^{VM}\right)^{T}\left(X_{u}^{(v)}-\Theta_{u}^{VM}\hat{\tau}_{u}^{VM}\right)$.

The minimum *AICVM u* in (S36) occurred at number *I* u*+*I’ uI’’* u*+*R* u*+*J* u*+*Q* u* of the real gene/miRNA regulations of EBV-miRNA *u* in the EBV-miRNA GRN. Therefore, the insignificant regulations in the real system orders *I* u*, *I’ uI’’* u*, *R* u*, *J* u*, and *Q* u* were pruned away one EBV-miRNA at a time to obtain the real EBV-miRNA GRN.
